# InbR, a TetR family regulator, binds with isoniazid and influences multidrug resistance in *Mycobacterium bovis* BCG

**DOI:** 10.1038/srep13969

**Published:** 2015-09-10

**Authors:** Min Yang, Chun-Hui Gao, Jialing Hu, Lei Zhao, Qiaoyun Huang, Zheng-Guo He

**Affiliations:** 1 National Key Laboratory of Agricultural Microbiology, Center for Proteomics Research, College of Life Science and Technology, Huazhong Agricultural University, Wuhan 430070, China; 2 School of Life Sciences and CAS Key Laboratory of Innate Immunity and Chronic Disease, University of Science and Technology of China, Hefei, Anhui 230027, China

## Abstract

Isoniazid (INH), an anti-tuberculosis (TB) drug, has been widely used for nearly 60 years. However, the pathway through which *Mycobacterium tuberculosis* responds INH remain largely unclear. In this study, we characterized a novel transcriptional factor, InbR, which is encoded by Rv0275c and belongs to the TetR family, that is directly responsive to INH. Disrupting *inbR* made mycobacteria more sensitive to INH, whereas overexpressing *inbR* decreased bacterial susceptibility to the drug. InbR could bind specifically to the upstream region of its own operon at two inverted repeats and act as an auto-repressor. Furthermore, InbR directly bind with INH, and the binding reduced InbR’s DNA-binding ability. Interestingly, susceptibilities were also changed by InbR for other anti-TB drugs, such as rifampin, implying that InbR may play a role in multi-drug resistance. Additionally, microarray analyses revealed a portion genes of the *inbR* regulon have similar expression patterns in *inbR*-overexpressing strain and INH-treated wild type strain, suggesting that these genes, for example *iniBAC*, may be responsible to the drug resistance of *inbR*-overexpressing strain. The regulation of these genes by InbR were further assessed by ChIP-seq assay. InbR may regulate multiple drug resistance of mycobacteria through the regulation of these genes.

*Mycobacterium tuberculosis* is the causative agent of tuberculosis (TB), one of the deadliest diseases worldwide. The emergence of multidrug-resistant (MDR) TB and extensive drug-resistant (XDR) TB is a large challenge to TB treatment^1^. Isoniazid (INH) is the most widely used first-line anti-TB drug. INH is structurally simple and activated by KatG. It forms an adduct with NAD, which inhibits the *inhA*-encoded NADH-dependent enoyl-ACP reductase^2^. Acquired INH resistance in *M. tuberculosis* is mainly caused by sequential accumulation of mutations in bacterial target genes^3^. However, additional regulatory mechanisms underlying drug resistance in *M. tuberculosis* are largely unclear, and the regulators involved in drug resistance remain unidentified.

Recent studies have found that an important mechanism by which bacteria acquire drug resistance is the active efflux of drugs by multidrug transporters^4^. For example, the operon *iniBAC* encodes transport-related genes in mycobacteria and confers tolerance against multiple anti-TB drugs to |*M. tuberculosis* and *M. bovis* BCG^5^. A two-component regulator named MtrA can recognize a motif within the upstream promoter region of *iniBAC* and can regulate mycobacterial sensitivity to multiple anti-TB drugs^6^. Several transcription factors are reportedly involved in the regulation of drug transporters or resistance genes in other bacteria, such as TetR and EmrR in *Escherichia coli*^7^,^8^, QacR in *Staphylococcus aureus*^9^, and BmrR in *Bacillus subtilis*^10^. These regulators inhibit or stimulate the expression of their target genes to contribute to bacterial drug resistance.

TetR is a large family of transcriptional regulators that contains a conserved helix-turn-helix DNA-binding domain and a C-terminal ligand regulatory domain. These regulators usually serve as repressors and are widely distributed among bacteria^11^. The most frequently characterized function of TetR proteins is regulation of efflux pumps and transporters, which are involved in antibiotic resistance and toxic chemical compound tolerance^12^,^13^,^14^,^15^. For example, the TetR of *E. coli* controls expression of the gene encoding a tetracycline efflux pump responsible for drug resistance^16^. TetR binds to the promoters of efflux pump genes and is regulated by a plethora of ligands that can cause protein conformational changes and eradicate protein binding, thereby relieving its repression of transcription^17^. In *M. tuberculosis*, the transcription factor Rv3066, which belongs to the TetR family, was recently found to bind specific co-activator drug molecules (ethidium) and to regulate bacterial drug resistance^18^. Rv3066 can directly bind ethidium and can de-repress the expression of a multidrug transporter operon, *mmr*^19^. The genomes of both *M. tuberculosis* and *M. bovis* BCG encode a large group of TetR family regulators^20^,^21^. However, transcription factors that can directly bind the first-line anti-TB drugs remain uncharacterized, and the molecular network through which the bacteria respond to the drugs remain largely unclear in *M. tuberculosis* and related mycobacterial species.

*M. bovis* BCG is a vaccine strain^21^ that has been used as a model strain for studying gene regulatory mechanisms in mycobacterial species, including the pathogenic strain *M. tuberculosis*. In this study, using *M. bovis* BCG as a model strain, we screened and characterized InbR, the first INH-binding transcriptional factor that regulates mycobacterial susceptibility to multiple drugs. The results showed that InbR functions as a repressor, and while its overexpression decreased bacterial susceptibility to INH, and its disruption led to supersensitivity of *M. bovis* BCG to INH. InbR was found to regulate the expression of multiple genes, including the *iniBAC* operon. Furthermore, we proposed an INH-inducible sequential signal cascade, in which InbR functions as a master regulator and plays an important role in the regulation of mycobacterial susceptibility to multiple anti-TB drugs.

## Results

### InbR positively regulates INH resistance in *M. bovis* BCG

Only a few transcription factors have been reported to contribute to mycobacterial drug resistance to date. To identify potential regulators that contribute to *M. tuberculosis* INH resistance, we screened a transcriptional factor library by spotting recombinant *M. bovis* BCG strains, in which the corresponding transcriptional regulator was overexpressed by the constitutive strong promoter *hsp60*^21^, on plates containing INH (2 μg/ml). First, all the annotated putative transcriptional regulators (approximately 300 ORFs) of *M. tuberculosis* were cloned in a batch into the overexpressing plasmid pMV261. Secondly, each recombinant strain was spotted onto 7H10 agar plates that contained 2 μg/ml INH. As a result, those strains that were more resistant to INH were able to grow and thus were identified as primary candidates. To avoid eventual random mutations that may confer drug resistance, the assays were repeated three times for primary candidates, and finally the drug susceptibility of recombinant strains were attributed to the overexpression of the candidate genes.

**Figure 1:**
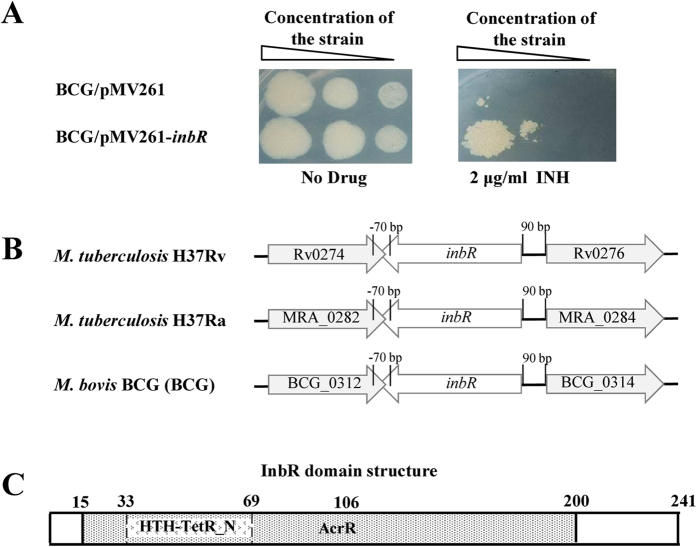
Effect of InbR on INH resistance of *M. bovis* BCG and its domain structure. **(A)** INH resistance of the *inbR-*overexpressing strain. Freshly grown recombinant strains of *M. bovis* BCG /pMV261 and *M. bovis* BCG/pMV261-*InbR* were two-fold diluted to three different concentrations, and equal amounts of culture were spotted on 7H10 plates with Kan (30 μg/ml) in the absence (left panel) or presence of 2 μg/ml INH (right panel). (**B)** The InbR orthologs in *M. tuberculosis* H37Rv, *M. tuberculosis* H37Ra, and *M. bovis* BCG. **(C)** Analysis of the structural characteristics of InbR. It contained a TetR_N superfamily domain within an AcrR domain.

A TetR family transcription factor encoded by Rv0275c, designated as InbR, was isolated. As shown in Fig. 1A, we measured the growth of *inbR*-overexpressing and pMV261 empty vector *M. bovis* BCG strains on the surface of a solid agar medium with or without INH. When a gradient of different concentrations of mycobacterial strains was spotted on the surface of a solid agar medium without INH, similar bacterial lawns were observed for both the *inbR*-overexpressing and pMV261 empty vector strains (Fig. 1A, left panel). By contrast, while the same concentration gradient of mycobacterial strains were spotted on a plate containing 2 μg/ml INH, the bacterial lawn for the pMV261 empty vector BCG strain was smaller than that for the *inbR*-overexpressing strain, indicating that the strain overexpressing *inbR* was more resistant to INH than the wild-type strain (Fig. 1A, right panel). This finding suggested that InbR was potentially involved in the regulation of INH-drug resistance in *M. bovis* BCG.

Orthologs of Rv0275c (InbR) were identified based on sequence similarity and the conservation of adjacent genes. Strikingly, InbR and its orthologs were found to be transcribed divergently from a hypothetical protein (Fig. 1B). The Rv0275c region is highly conserved within *M. tuberculosis* H37Rv, *M. tuberculosis* H37Ra, and *M. bovis* BCG (100% amino acid identity over the entire length of the protein), but not in *Mycobacterium smegmatis* (53% amino acid identity). The gene *inbR* encodes a 241-residue protein containing a typical TetR_N superfamily domain within an AcrR domain (Fig. 1C), which suggests that InbR belongs to the TetR/AcrR family of transcription factors.

**Figure 2:**
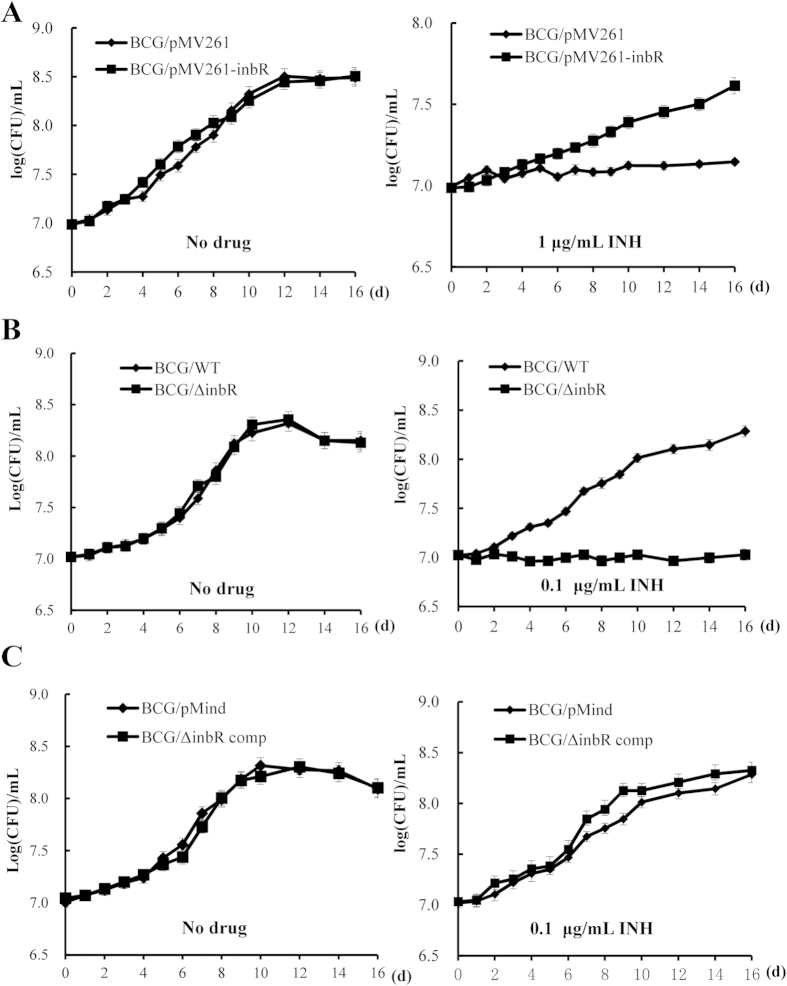
Assays for the effects of InbR on INH resistance in *M. bovis* BCG. Wild-type, *inbR*-overexpressing (**A**), *inbR*-deleted (**B**) and complementary (**C**) mycobacterial strains were grown in 7H9 media with or without INH. Growth curves of the recombinant strains were determined as described in the Materials and Methods section. Representative data are shown. Error bars represent the standard deviation across three biological replicates.

We further assayed the regulatory effect of InbR on the growth of *M. bovis* BCG in response to INH by determining mycobacterial growth curves. Prior to this assay, the *M. bovis* BCG *inbR*-deleted mutant strain (BCG/Δ*inbR*) was obtained (Fig. S1), together with the complementary strain (BCG/Δ*inbR* comp). As shown in Fig. 2, no obvious difference was observed in the growth of the pMV261 empty plasmid and *inbR*-overexpressed BCG strains in 7H9 medium in the absence of drugs (Fig. 2A, left panel). However, compared with the pMV261 empty plasmid strain, the *inbR*-overexpressed BCG strain grew significantly better than the pMV261 empty plasmid strain in 7H9 medium containing 1 μg/ml of INH (Fig. 2A, right panel; *p* < 0.05). Without INH, the growth of the *inbR*-deleted strain (BCG/Δ*inbR*) and wild type (BCG/WT) have similar growth curves (Fig. 2B, left panel). With 0.1 μg/ml of INH, the growth of the *inbR*-deleted strain was significantly inhibited compared with that of the wild type (Fig. 2B, right panel). Additionally, this type of inhibition can be complemented in the complemented strain (Fig. 2C). Moreover, overexpression of *inbR* decreased the INH susceptibility of the *M. tuberculosis* H37Ra strain as well (Fig. S2). These results are consistent and indicated that InbR positively regulates INH resistance in *M. bovis* BCG.

### InbR recognizes a palindromic motif and specifically binds to its promoter as an auto-repressor

**Figure 3:**
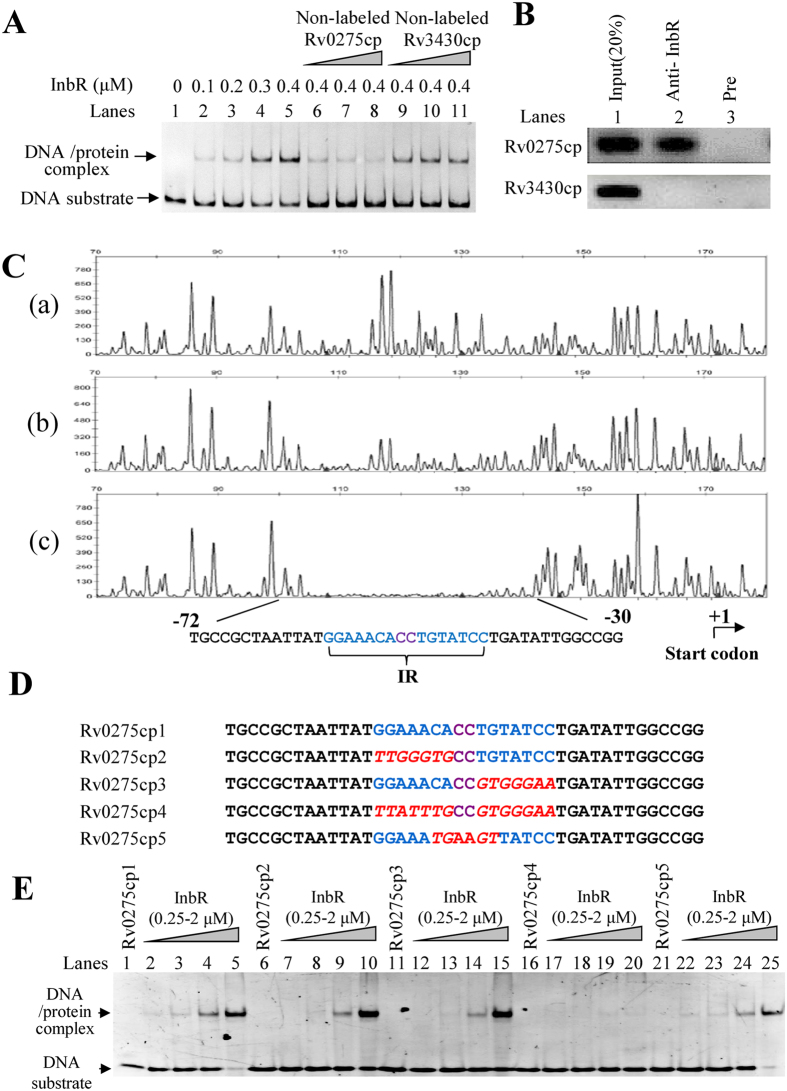
Assays for auto-regulation of InbR. (**A**) EMSA assays for specific DNA-binding activity of InbR on the upstream region of the *inbR* operon (Rv0275cp). FITC-labeled Rv0275cp DNA substrate was co-incubated with InbR (lane 1 to lane 5). Unlabeled upstream region DNA (lane 6 to lane 8), but not an unrelated Rv3430c promoter (lane 9 to lane 12), could compete with the labeled upstream region DNA for binding with InbR. (**B**) ChIP assays. ChIP using preimmune or immune sera raised against InbR. The mycobacterial promoter Rv3430cp was used as a negative control. (**C**) Dye primer-based DNaseI footprinting experiments. Protection of the InbR promoter DNA against DNaseI digestion by increasing amounts of InbR (0 μM, 1.5 μM, and 3.0 μM) was evaluated. The sequences of the protected regions on the coding strand are underlined. (**D**) Sequence of the short DNA substrates used in the following EMSA assays. (**E**) EMSA assays on DNA substrates with or without the IR sequence. Each DNA substrate was co-incubated with 0.25–2 μM InbR protein.

In mycobacteria, many TetR family transcriptional factors possess an auto-regulating function. We used an electrophoretic mobility shift assay (EMSA) to examine the binding of the InbR (Rv0275c) protein to the upstream region of its own operon *in vitro*. As shown in Fig. 3A, when 3 nM upstream DNA substrates (Rv0275cp) were co-incubated with increasing amounts of InbR (0 μM, 0.1 μM, 0.2 μM, 0.3 μM, and 0.4 μM), clear shift bands were observed (Fig. 3A, lane 2 to lane 5). A competition assay confirmed the specificity of InbR binding to its promoter DNA. Unlabeled specific Rv0275cp or unspecific Rv3430c promoter DNA substrate (Rv3430cp) was used to compete with the labeled Rv0275cp. As shown in Fig. 3A, unlabeled DNA substrate (Fig. 3A, lane 6 to lane 8), but not Rv3430c promoter DNA (Fig. 3A, lane 9 to lane 11), could competitively inhibit the binding of InbR to the labeled upstream DNA of *inbR* operon. Further chromatin immunoprecipitation (ChIP) assays confirmed the binding of InbR to the upstream DNA of *inbR* operon *in vivo*. As shown in Fig. 3B, InbR could be crosslinked with the upstream DNA Rv0275cp in *M. tuberculosis*. The promoter DNA could be recovered by immunoprecipitation with InbR antiserum (Fig. 3B, lane 2). By contrast, the pre-immune serum failed to precipitate significant amounts of DNA (Fig. 3B, lane 3). In addition, Rv3430cp, the promoter of an unrelated gene, used as negative control, could not be recovered with InbR antiserum. These findings strongly suggested that InbR could bind with its own promoter region. By using β-galactosidase assays, we further characterized that InbR functions as an auto-repressor (Fig. S3).

We characterized the DNA binding motif of InbR by Dye primer-based DNase I footprinting assays. As shown in Fig. 3C, when increasing amounts of InbR protein (0–2 μM) were co-incubated with DNaseI, the region around TGCCGCTAATTATGGAAACACCTGTATCCTGATATTGGCCGG was obviously protected on the coding strand. The protected DNA region extended from position −72 to −30 in the DNA strand (Fig. 3C). A palindromic motif formed by two inverted repeats partially matched, which was separated from each other by two nucleotides, was found in this region. Further EMSA assays confirmed the significance of the motif for specific recognition by InbR. DNA substrate mutants were synthesized (Fig. 3C) and EMSA assays were conducted (Fig. 3D). As shown in Fig. 3D (right panel, Lane 6–10), InbR lost the ability to bind with Rv0275cp4 in which the two inverted repeats were replaced by random sequences. By contrast, replacement of either part of the repeat or the interspaced sequence did not abolish their interaction (Fig. 3D, lane 6–15, lane 21–25), although the binding was a little bit weaker compared with that of inherent Rv0275cp1. These results suggested that the binding of InbR may be not very precise and a flexible and partial mismatch is allowed.

In conclusion, InbR is an auto-repressor and the auto-regulation of InbR relies on a palindromic sequence motif.

### InbR directly binds INH and the binding represses its DNA-binding activity

As far as we know, overexpression of *inbR* increase INH resistance. On this basis, we further examined whether INH induced the expression of *inbR* in *M. bovis* BCG by quantitative RT-PCR (qRT-PCR). *M. bovis* BCG strains were grown until the logarithmic growth phase (OD_600_ = approximately 0.6) and various concentrations of INH (0.5 μg/ml, 1 μg/ml, and 2 μg/ml) were added to the medium. Cells were harvested 24 h later and qRT-PCR was performed. We found that *inbR* induction was increased by 1.2-fold, 1.67-fold, and 4.08-fold under INH concentrations of 0.5 μg/ml, 1 μg/ml, and 2 μg/ml, respectively (Fig. S4). The results implied that high concentrations of INH will significantly induce the expression of *inbR in vivo* and interactions between InbR and INH are possibly present.

**Figure 4:**
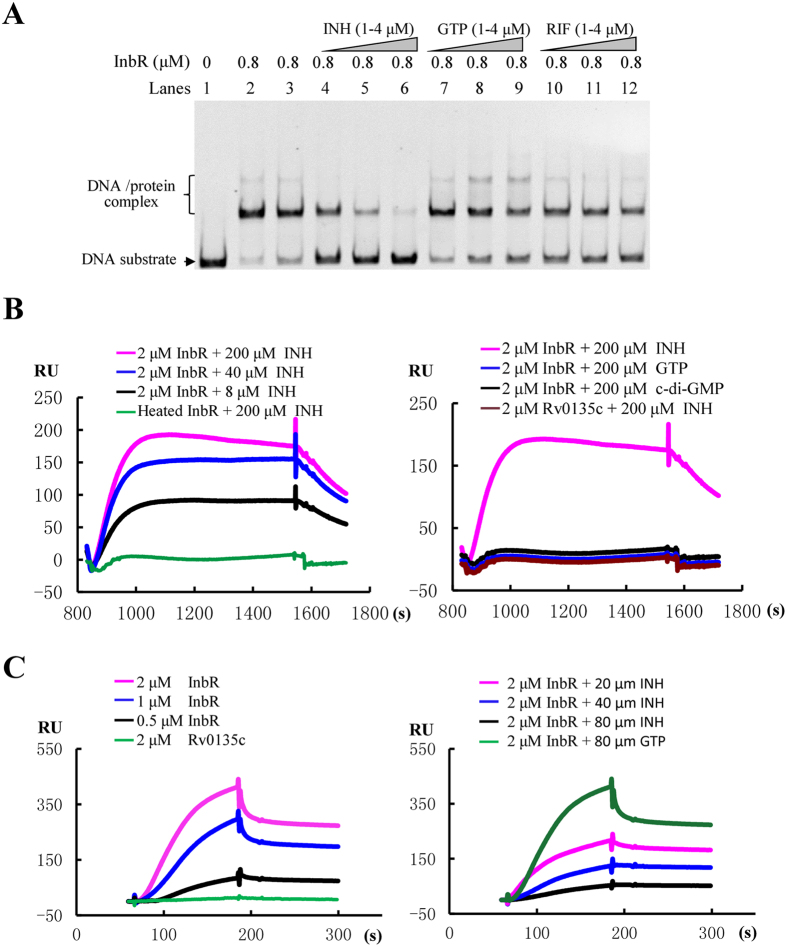
Effects of INH on the DNA-binding activity of InbR. (**A**) EMSA assays. FITC-labeled InbR upstream promoter DNA substrate (Rv0275cp) was co-incubated with InbR in the absence (lane 1 to lane 3) or presence of INH (1 μM to 4 μM; lane 4 to lane 6), GTP (1 μM to 4 μM; lane 7 to lane 9) or RIF (1 μM to 4 μM; lane 10 to lane 12). (**B**) SPR assays. InbR was immobilized on NTA sensor chip and small molecules were flow through. GTP and c-di-GMP were control molecules. (**C**) SPR assays. Biotin-labelled Rv0275cp was immobilized on the SA chip. Different concentrations of InbR (0.5 μM to 2 μM; left panel) or a fixed concentration of InbR (2 μM) along with 20–80 μM INH (right panel) were passed over the chip surface. Rv0135c was used as the negative control protein. GTP was used as negative control molecule.

EMSA assays were subsequently conducted to check the possible interaction between INH and InbR. As shown in Fig. 4A, when 3 nM upstream DNA of the *inbR* operon (Rv0275cp) was co-incubated with 0.8 μM InbR, two clear shifted bands were observed (Fig. 4A, lane 2 to lane 3). Adding increasing amounts of INH (1 μM to 4 μM) led to a corresponding decrease in the amounts of shifted DNA substrates (Fig. 4A, lane 4 to lane 6). This finding indicated that INH inhibited the DNA-binding activity of InbR in a concentration-dependent manner (Fig. 4A, lane 5 to lane 7). By contrast, GTP (lane 7 to lane 9) and rifampicin (RIF; lane 10 to lane 12) slightly affected the DNA-binding activity of InbR. Moreover, the addition of ethambutol (EMB) did not affect InbR as well (Fig. S5). These results indicated that INH inhibited the ability of InbR DNA binding activity.

Furthermore, surface plasmon resonance (SPR) assays were conducted to verify the interaction of InbR and INH. As shown in Fig. 4B, when increasing amounts of INH were passed over the 6 × His-InbR-immobilized NTA chip, a corresponding increase in response was observed. In particular, when 200 μM INH was passed over the chip, a response of approximately 200 RU was observed (Fig. 4B, left panel). Furthermore, K_d_ for the specific interaction between InbR and INH was 0.72 μM, indicating strong binding affinity. No response was obtained when either a heat-denatured InbR protein (Fig. 4B, left panel) or a negative control protein; i.e., Rv0135c, was immobilized on the chip (Fig. 4B, right panel). Consistently, no response was observed when the same amount of unrelated small molecules, such as guanosine-5′-triphosphate (GTP) or cyclic diguanylate monophosphate (c-di-GMP), was passed over the His-InbR-immobilized NTA chip (Fig. 4B, right panel). The results showed that InbR directly binds INH.

In addition, SPR experiments were conducted with an immobilized promoter DNA on a chip and InbR in different conditions. InbR promoter DNA was immobilized on the SA chip, and proteins with or without small molecules were passed over. As shown in Fig. 4C, when increasing concentrations of the InbR protein (0.5 μM to 2 μM) were passed, corresponding increases in response values were observed (Fig. 4C, left panel). By contrast, unrelated Rv0135c protein did not show any response when passed over the chip. In addition, when InbR was treated by increasing concentrations of INH (20 μM to 80 μM INH co-incubated with 0.4 μM InbR) prior to use, corresponding decreases in response values were observed (Fig. 4C, right panel). By contrast, with identical treatment by an unrelated small molecule GTP, the response value did not change (Fig. 4C, right panel).

These results jointly indicated that InbR binds INH and the binding represses its DNA-binding ability.

### The function of InbR is not INH-specific and the mode of action is complicated

Although InbR does not bind either EMB or RIF, relationships between InbR and the drugs still exist. Minimal inhibitory concentrations (MIC) of wild type, *inbR*-overexpressing, *inbR*-deleted and complementary strains were tested with INH, RIF, EMB and mitomycin C (MMC). The MICs of the *inbR*-deleted strain were all lower compared with those of the wild-type strain (Table 1). By contrast, the MICs of the *inbR*–overexpressing strain were all higher than that of the wild-type (Table 1). Additionally, growth of the *inbR*-deleted strain was significantly inhibited by either EMB or RIF at a low concentration in which the wild type strain grew very well (Fig. S6). That is to say, disrupting the *inbR* gene made the *M. bovis* BCG strain more sensitive to multiple drugs, whereas overexpressing *inbR* decreased the susceptibility, and the results suggested that the function of InbR is not INH-specific.

To further elucidate the mechanism by which InbR regulates drug resistance, we performed microarray analyses on *inbR*-overexpressing and INH-treated wild type *M. bovis* strains. While comparing the results with that of the non-treated wild type strain, many genes that had consistent expression profiles were identified (Table 2 and Table S5). On the one hand, ribosomal proteins, including S18, L9, S19, L22, S3, L16, L29, S17 and L30, *iniBAC* and several hypothetical proteins were upregulated in both *inbR*-overexpressing and INH-treated strains. On the other hand, a large number of metabolic enzymes, including *pqqE*, *lldD1*, *echA7*, *gltA1*, *fadE12*, *accA2*, *accD2*, *gltA1*, *narG*, *narH* and *narJ*, and regulatory proteins such as *sigI*, *pfkB*, *devR* were all downregulated. Moreover, there were also many genes with expression profiles that are different in *inbR*-overexpressing and INH-treated strains. For example, *argJ*, *argB*, *argD*, *argE* and *argR* are only upregulated in the former strain. These results suggest InbR uses a complex network to conduct multiple levels of regulation.

**Figure 5:**
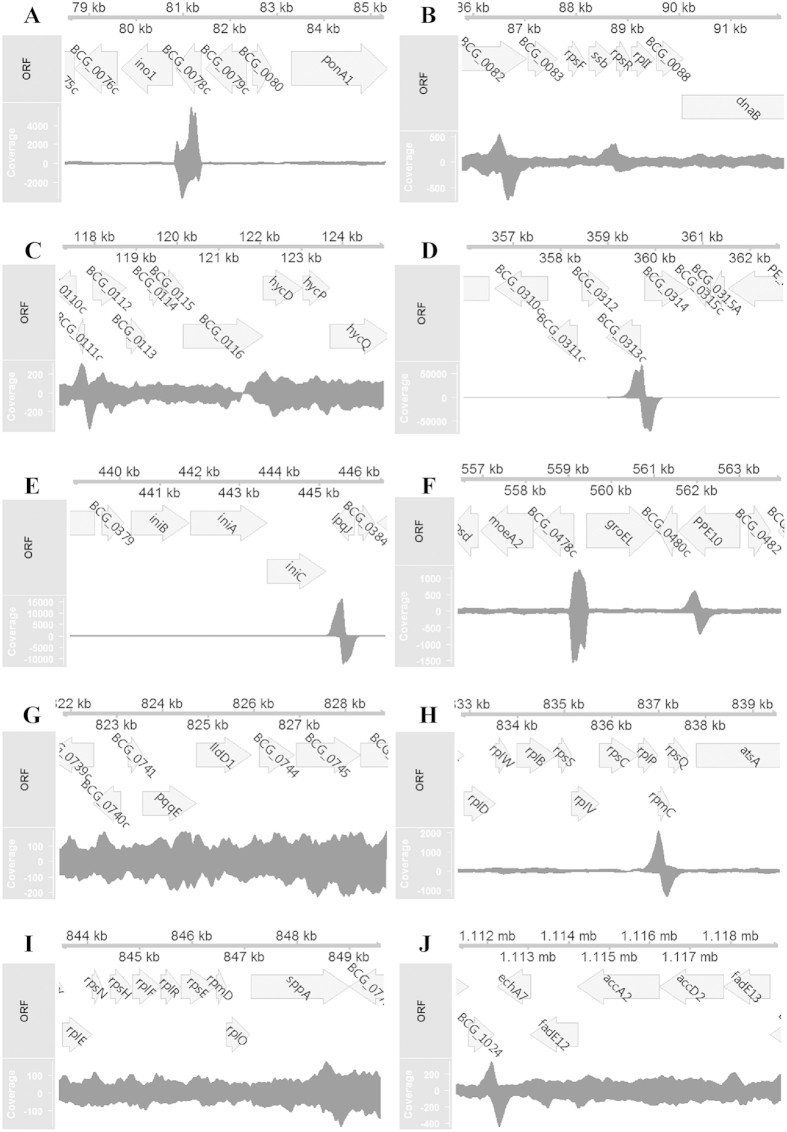
ChIP-seq assay. Genome contexts and short read coverage are shown for the first 10 featured gene cluster/operon ((**A**–**J**) correspond with No. 1–10 in Table 2) that have been listed in Table 2. Coverage were plotted in different strands, and the values are shown on the left. Plots were generated by R tools Gviz.

In addition, a ChIP-seq assay was conducted with an *inbR*-overexpressing strain to determine its direct binding targets. Peaks were found in an upstream region of multiple ribosomal protein genes, chaperonin, and regulatory proteins (Table 2), implying they are direct targets of InbR. For example, BCG_0079c (Fig. 5A), *rpsR*/*rplI* (Fig. 5B), BCG_0114–18 (Fig. 5C), *inbR* (Fig. 5D), *groEL* (Fig. 5F), BCG_0755-60 (Fig. 5H) and BCG_1028c-25c (Fig. 5J) are direct targets of InbR, while BCG_0741-45 (Fig. 5G), and BCG_0772-73 (Fig. 5I) are indirect targets (see also: Table S5). Interestingly, the assay revealed a high quality peak (qvalue = 1.4E5) downstream *iniBAC* (Table 2, Fig. 5E and Table S5). Moreover, gene ontology (GO) analysis revealed peaks that were associated with genes that were enriched at the GO term “small molecule binding” (P = 1.45E-8).

**Figure 6:**
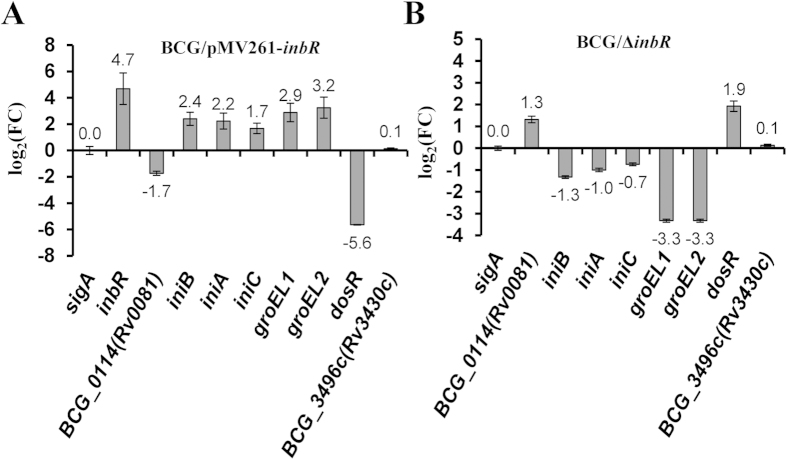
qRT-PCR assays. The expression of several important genes including *rv0081*, *dosR*, *groEL*1, and the *iniBAC* operon was determined in the *inbR*-overexpressing (**A**) and *inbR-*deletion (**B**) *M. bovis* BCG strains.

We performed qRT-PCR assays to verify the differential expression of several important genes in the *inbR*-overexpressing and the *inbR*-deleted strains. On the one hand, expression of *Rv0081* and *dosR* were downregulated (0.3-fold or 0.02-fold) in the *inbR*-overexpressing strain (Fig. 6A), and upregulated (2.5-fold or 3.8-fold) in the *inbR*-deleted strain (Fig. 6B). On the other hand, the expression of the *groEL1*, *groEL2* and *iniBAC* operons was upregulated in the *inbR*-overexpressing strain (Fig. 6A), and downregulated in the *inbR*-deleted strain (Fig. 6B). These qRT-PCR results were consistent with the results of the microarray and showed that whenever a gene is a direct or indirect target, there is regulation by InbR.

In summary, InbR regulates bacterial susceptibility to multiple anti-TB drugs in *M. bovis* BCG, via regulation of a large number of genes.

## Discussion

The molecular network through which *M. tuberculosis* responds to anti-TB drugs and the intrinsic regulatory mechanism underlying mycobacterial INH resistance remain largely unclear. In the present study, we report a TetR family regulator; i.e., InbR, which interacts directly with the first-line anti-TB drug INH in *M. bovis* BCG. Overexpression of *inbR* decreased mycobacterial INH susceptibility, whereas disrupting *inbR* made the mycobacteria supersensitive to multiple anti-TB drugs. Most interestingly, we provide evidence that INH can directly bind to InbR and negatively affects the regulator’s DNA-binding ability. Thus, we have uncovered a novel mechanism underlying regulation of mycobacterial susceptibility to INH.

The TetR/AcrR family regulators usually function as repressors and are widely distributed among many bacteria^11^. Most of these proteins are involved in the regulation of drug resistance, biosynthesis of antibiotics, osmotic stress, and bacterial pathogenicity^11^. The AcrR operon of *E. coli* contains three genes; namely, *acrR*, *acrA*, and *acrB*, the last two of which are multidrug resistant efflux pumps^22^,^23^. By comparison, InbR has a typical AcrR domain but, unlike in *E. coli*, is encoded in a single operon. Targets of InbR were, therefore, going to be elucidated. In the present study, we provided evidence to show that InbR acts as an auto-repressor and regulates the expression of a large number of genes. Among these genes, many overlapping genes of InbR regulon genes and INH responsive genes were identified (Table 2 and Table S5). INH responsive genes such as *iniBAC,* have been shown to be involved in tolerance to multiple anti-TB drugs^5^. Therefore, similar expression profiles for these genes may also give multiple drug resistance to *inbR*-overexpressing strains. In the InbR regulon, some are direct targets, while the others are indirect targets. Many genes are not drug specific genes in mycobacteria but play roles in multiple stress adaptation. In addition, a ChIP-seq assay revealed that direct targets of InbR are enriched in the GO term small molecule binding. This result implied that the binding of small molecules play an important role in InbR’s mode of action. Therefore, other types of small molecules may be preventing targets of InbR regulon genes as well. Additionally, this could be an acceptable explanation for InbR INH-nonspecific functions.

As has been revealed by microarray analysis and qRT-PCR results, InbR could strongly induce the expression of the operon *iniBAC* (Table 2 and Fig. 6). The *iniBAC* operon encodes transport-related genes in mycobacteria and confers multiple anti-TB drug tolerance to *M. tuberculosis* and *M. bovis* BCG^5^. It is believed that the effect of InbR on multidrug resistance in *M. bovis* BCG are, mainly or partially, due to the overexpression of the *iniBAC* operon. Interestingly, a subsequent ChIP-seq assay revealed a high quality peak (qvalue = 1.4E5) downstream of *iniBAC*. Therefore, the regulation of *iniBAC* is distinct; for example, by antisense RNA. Moreover, InbR may also regulate *iniBAC* indirectly. For example, five regulators were reported as regulators for *iniBAC* in TBDB (http://TBDB.org, Rv0081, Rv0967, Rv1353c, Rv1956 and Rv2250c^37^,^38^), in which Rv1956 and Rv1353c were the direct targets of InbR (ChIP-seq peaks found upstream, Table S5), while Rv0081 and Rv0967 were the indirect targets of InbR (down- and upregulated in *inbR*-overexpressing strain, respectively, Table S5). Although the details were not very clear, it is logical to conclude InbR may regulate *iniBAC* expression through direct and/or indirect pathways.

One interesting finding is that InbR could regulate susceptibilities of multiple drugs. As we know, drug resistance in *M. tuberculosis* results primarily from acquisition of chromosomal mutations in genes encoding the drug target proteins, such as *katG* and *inhA*^24^,^25^,^26^. Nonetheless, gene expression changes were also thought to introduce drug resistance. For example, downregulation of *katG* was found to be highly associated with isoniazid resistance in *M. tuberculosis*^27^. Moreover, *whiB7* was believed to be one of the main causes of mycobacterial intrinsic drug resistance^28^. In general, the affection for a transcriptional regulator to drug resistance is quite different from an enzymatic gene such as *katG*. The effect of *katG* follows a very simple rule: the activation of pro-drug INH. Inactivation of *katG* leads to defects in INH activation thus introducing drug resistance. By contrast, the effect of a transcriptional regulator would be much more complex. In living cells, regulators set up a network and work jointly, which is flexible and stable. Omitting a single regulator that is not lethal may not affect the function of such a network. In this study, we found InbR could bind INH and positively regulate drug resistance in mycobacteria. Molecular mechanisms were also investigated and several clues were found; however, the biological role for this novel regulator InbR is still not fully understood and further studies are needed.

In conclusion, this study showed that the TetR-family transcriptional regulator InbR binds isoniazid and influences multidrug resistance in *M. bovis* BCG.

## Experimental Procedures

### Strains, plasmids, enzymes and reagents

*E. coli* BL21 (λDE3) cells and pET28a were purchased from Novagen (Darmstadt, Germany) and were used to express proteins. Restriction enzymes, T4 ligase, modification enzymes, DNA polymerase, dNTPs, and all antibiotics were obtained from TaKaRa Biotech (Shiga, Japan). PCR primers were synthesized by Invitrogen (Carlsbad, USA). Ni-NTA (Ni^2+^-nitrilotriacetate) agarose was purchased from Qiagen (Hilden, Germany). 7H9 and 7H10 broths were purchased from Becton, Dickinson Company (New Jersey, USA). Antibodies were obtained from the Wuhan laboratory animal center of CAS (Wuhan, China).

### Cloning, Expression and Purification of Recombinant Proteins

The regulatory genes were amplified by PCR using specific primers from genomic DNA of *M. tuberculosis* H37Rv and were cloned into pET28a to produce recombinant vectors (Table S1 and S2). *E. coli* BL21 cells, which were transformed with the recombinant plasmid, were grown in 200 ml of LB medium up to OD_600_ of 0.6. Protein expression was induced by the addition of 0.3 mM isopropyl β-D-1-thiogalactopyranoside (TaKaRa). Harvested cells were resuspended and sonicated in binding buffer (20 mM Tris-HCl, pH 8.0; 100 mM NaCl; and 10 mM imidazole), and the lysate was centrifuged at 10,000 × g for 30 min. The cleared supernatant was loaded onto the affinity column. The column-bound protein was washed with buffer (20 mM Tris-HCl, pH 8.0; 100 mM NaCl; and 40 mM imidazole). The protein was then eluted using elution buffer (20 mM Tris-HCl, pH 8.0; 100 mM NaCl; and 250 mM imidazole). The elution was dialyzed overnight and stored at −80 °C. Protein concentration was detected with a Coomassie Brilliant Blue assay.

### Electrophoretic mobility shift assay (EMSA)

DNA substrates for DNA-binding activity assays were amplified by PCR from the genomic DNA of *M. tuberculosis* H37Rv or directly synthesized by Invitrogen (Table S3). The DNA substrates were labeled at the 5′-terminus with fluorescein isothiocyanate (FITC) and were stored at −20 °C until use. For EMSA assays, DNA substrates were incubated at 25 °C for 30 min or 1 h with various amounts of proteins in a total volume of 20 μL of EMSA buffer (50 mM Tris-HCl, pH 7.5; 10 mM MgCl_2_; 1 mM DTT; and 50 mM NaCl). The mixtures were directly subjected to 5% native PAGE containing 0.5× Tris-borate-EDTA buffer. Electrophoresis was performed at 150 V and 25 °C until the bromophenol blue band reached the bottom of the gel. The images were acquired using Typhoon Scanner (GE healthcare).

### ChIP-PCR and ChIP-seq assays

Chromatin immunoprecipitation (ChIP) was performed as described previously^29^ with modifications. *M. bovis* BCG cells were grown in 100 ml 7H9 medium, fixed with 1% formaldehyde, and stopped with 0.125 M glycine. Crosslinked cells were harvested and resuspended. The sample was sonicated on ice and the average DNA fragment size was determined to be approximately 0.5 kb. A 100 μl sample of the extract was saved as the input fraction, whereas the remaining 900 μl was incubated with 10 μl of antibodies against corresponding proteins or preimmune serum under rotation for 3 h at 4 °C. The complexes were immunoprecipitated with 20 μl 50% protein A agarose for 1 h under rotation at 4 °C. The immunocomplex was recovered by centrifugation and resuspension in 100 μl TE (20 mM Tris–HCl, pH 7.8; 10 mM EDTA; and 0.5% SDS). Crosslinking was reversed for 6 h at 65 °C. The DNA samples of the input and ChIP were purified, resuspended in 50 μl TE, and analyzed by PCR with Platinum Taq (Invitrogen). The amplification protocol included one denaturation step of 5 min at 95 °C, then 32 cycles of 1 min at 95 °C, 1 min at 60 °C, and 1 min at 72 °C.

For the ChIP-seq assay, ChIP-enriched DNA was obtained similarly, except that the fragment size was approximately 300 bp, which is the desired size for Illumina short DNA library construction. Sequencing libraries were constructed following the manufacturer’s instruction and then subject to Illumina HiSeq2000/2500 instruments (BGI, Shenzhen, China). Short reads were aligned using Bowtie2^30^ and peaks were called by MACS^31^. Peaks were annotated using Bioconductor toolbox (http://bioconductor.org).

### Dye primer-based DNase I footprinting assay

The DNase I footprinting assay was performed as previously described^32^. A 420-bp fluorescently labeled DNA fragment that encompassed bases −200 to +200 of the translational start site of Rv0275c was generated by PCR amplification. The fluorescently labeled probe was subjected to the same binding reaction as in EMSA. Then, 0.0025 U of DNase I was added and incubated for 5 min at room temperature. The digested DNA fragments were purified. The samples were analyzed with the 3730 DNA analyzer coupled with a G5 dye set using an altered default genotyping module that increased the injection time to 30 s and the injection voltage to 3 kV. The 420-bp fragment was sequenced using special primers in the Thermo Sequenase Dye Primer Manual Cycle Sequencing Kit (USB, Inc., Cleveland, OH, USA) following the manufacturer’s instructions. Electropherograms were analyzed and aligned using the GENEMAPPER software (version 4.0, Applied Biosystems).

### Microarray analysis

Microarrays used in this study consisted of 15,744 60-mer probes, which were synthesized *in situ* by Agilent Technologies. The probes were designed based on the genome sequences of *M. bovis* BCG Pasteur_1173P2_uid58781 (GenBank accession numbers: NC_008769) and covered 3934 ORFs. Each probe was repeated thrice on the array. The *inbR-*overexpressing *M. bovis* BCG strain, *M. bovis* BCG wild-type strain, and INH-treated strain (*M. bovis* BCG wild-type strain grown on exponential phase OD_600_ ≈ 0.8 and treated with 0.5 μg/ml INH for 24 h) grown on exponential phase OD_600_ ≈ 1.2 were harvested. Total RNA was extracted and purified using an RNeasy mini kit (Cat. #74106, QIAGEN, GmBH, Germany) following the manufacturer’s instructions. RNA integrity was determined by utilizing RNA integrity number (RIN) generated using an Agilent Bioanalyzer 2100 (Agilent Technologies, Santa Clara, CA, US). Total RNA was amplified and labeled by Low Input Quick Amp Labeling Kit, One-Color (Cat. #5190-2305, Agilent Technologies) following the manufacturer’s instructions. Labeled cRNA (complementary RNA) were purified using the RNeasy mini kit.

Each slide was hybridized with 600 ng Cy3-labeled cRNA using a Gene Expression Hybridization Kit of Agilent Technologies (Cat. #5188-5242) according to the manufacturer’s instructions. After 17 h of hybridization with 15744 60-mer probes, slides were washed in staining dishes (Cat. #121, Thermo Shandon, Waltham, MA, US) with Gene Expression Wash Buffer Kit (Cat. #5188-5327, Agilent Technologies) following the manufacturer’s instructions. Slides were scanned using an Agilent Microarray Scanner (Cat. #G2565CA) with default settings; Dye channel: Green; Scan resolution = 5 μm; and PMT = 100% and 10%, 16 bit. Data were extracted with Feature Extraction software (ver. 10.7, Agilent Technologies). The raw data were normalized using the Quantile algorithm in the Gene Spring software (ver. 11.0, Agilent Technologies). Normalized microarray expression data deemed significant (P ≤ 0.05) from the *InbR*-overexpression *M. bovis* BCG or BCG exposed to INH were selected, and the genes with fold change >2.0 were selected for further analysis.

### Quantitative real-time PCR

Isolation of mRNA and cDNA from mycobacterial strains was performed as described previously^33^. For real-time PCR analysis, gene-specific primers (Table S4) were used, and first-strand cDNAs were synthesized using SuperScript II reverse transcriptase (Invitrogen) according to the manufacturer’s instructions. Each PCR reaction (20 ml) contained 10 ml of 2× SYBR Green Master Mix Reagent (Applied Biosystems), 1.0 ml of cDNA samples, and 200 nM gene-specific primers. The reactions were performed in a Bio-Rad IQ5 RT-PCR machine. The thermocycling conditions were 95 °C for 5 min; 40 cycles of 95 °C for 30 s, 60 °C for 30 s and 72 °C for 30 s. Amplification specificity was assessed by conducting melting curve analysis. Differential gene expression was normalized to the levels of 16S rRNA gene transcripts. The degrees of expression change were calculated using the 2^−ΔΔCt^ method^34^.

### Surface plasmon resonance (SPR) analysis

SPR analysis was carried out in a Biacore 3000 instrument (GE Healthcare) with nitrilotriacetic acid (NTA) and SA sensor chips as described previously^35^,^36^. The assays were performed at 25 °C. For the binding of INH with proteins, a His-tagged protein was immobilized onto NTA chips at densities of approximately 1,200 response units (RU). INH was used as the ligand and was diluted in HBS buffer (10 mM HEPES, pH 7.4; 150 mM NaCl; 50 μM EDTA; 5 mM ATP; and 0.005% BIAcore surfactant P20) at concentrations of 8 nM, 40 nM, and 200 nM, and injected at 10 μl/min for 5 min. GTP was substituted for INH in the negative control. An overlay plot was produced using BIAevaluation 3.1 software to depict the interaction between INH and proteins. To assess the binding of DNA with proteins, biotinylated Rv0275cp probes were immobilized onto streptavidin (SA) chips at densities of approximately 200 (RU). His-tagged Rv0275c, Rv0135c or His-tagged Rv0275c-INH, and Rv0275c-GTP, were diluted in HBS buffer at concentrations of 1, 2, 4, or 4 μM protein +0.2, 0.4, and 0.8 μM INH and injected at 10 μl/min for 5 min. GTP was substituted for INH in the negative control.

### Determination of the MIC of anti-TB drugs

MIC determination was performed as previously described^2^. Briefly, *M. bovis* BCG/pMV261, *inbR*-deleted mutant strain and *inbR*–overexpression strain were grown to OD_600_ of 1.0 and diluted to approximately 1 × 10^7^ cfu·ml^−1^. Then, 0.05 ml of the dilution was used to inoculate 5 ml of Middlebrook 7H9 media containing various concentrations (0–1.28 μg ml^−1^) of four anti-TB drugs, namely, INH, RIF, EMB, and MMC. The cultures were incubated while shaking at 37 °C for 2 weeks. The MIC was calculated as the concentration of each drug that inhibited bacterial growth.

### Determination of mycobacterial growth curves and the effect of antibiotics

To determine mycobacterial growth curves and the effect of antibiotics, the recombinant strains were grown for a week in Middlebrook 7H9 media (supplemented with 10% albumin dextrose catalase, 0.05% Tween-80, and 0.2% glycerol) containing 30 μg/ml Kan. Cells were cultured to an OD_600_ between 1.5 and 2.0, and each culture was diluted (4:100) in 100 ml of fresh 7H9 broth. The cultures were then allowed to grow further at 37 °C with shaking at 200 rpm. When cells entered a log growth phase (OD_600_ of approximately 0.4), the indicated concentration of each antibiotic was added. The cultures were then allowed to grow further at 37 °C with shaking at 120 rpm. Aliquots were obtained at the indicated times, and the cultures were plated on 7H10 medium (supplemented with 0.2% glycerol) to determine colony-forming units^33^.

## Additional Information

**Accession codes:** The raw data of ChIP-seq and microarray experiments were deposited in NCBI SRA and GEO databases under accession PRJNA284806 and GSE69379, respectively.

**How to cite this article**: Yang, M. *et al.* InbR, a TetR family regulator, binds with isoniazid and influences multidrug resistance in *Mycobacterium bovis* BCG. *Sci. Rep.* **5**, 13969; doi: 10.1038/srep13969 (2015).

## Supplementary Material

Supplementary Information

## Figures and Tables

**Table 1:** Determination of the MIC of four anti-TB drugs.

**Strain**	MIC (μg/ml)*			
	**INH**	**RIF**	**EMB**	**MMC**
BCG/WT	0.04	0.008	0.32	0.064
BCG/ΔinbR	0.01	0.002	0.08	0.016
BCG/pMV261-inbR	0.16	0.016	0.64	0.128
BCG/△inbR comp	0.04	0.008	0.32	0.064

^*^M. bovis BCG was diluted to 1 × 10^5^ cfu ml^−1^ and was used to inoculate 3 ml of Middlebrook 7H9 media containing various concentrations (0–1.28 μg ml^−1^) of four anti-TB drugs.

**Table 2:** Expression patterns of 20 featured gene clusters in *inbR*-overexpressed and INH induced strains.

*No.*	*BCG ORF*	Log2(FC)*					
		***inbR* over-expressed**	**INH induced**	***MTB ORF***	***Gene***	***Function***	Peaks**
1	BCG_0079c	1.01	1.24	Rv0048c		hypothetical protein	In
2	BCG_0086	1.91	2.14	Rv0055	*rpsR*	30S ribosomal protein S18	Up
	BCG_0087	1.52	1.53	Rv0056	*rplI*	50S ribosomal protein L9	
3	BCG_0114	−1.89	−1.56	Rv0081		transcriptional regulatory protein	Up
	BCG_0115	−1.36	−1.36	Rv0082		oxidoreductase	
	BCG_0116	−1.69	−1.25	Rv0083		oxidoreductase	
	BCG_0117	−1.09		Rv0084	*hycD*	formate hydrogenlyase hycD (FHL)	
	BCG_0118	−1.03		Rv0085	*hycP*	hydrogenase hycP	
4	BCG_0313c	7.55		Rv0275c		TetR family transcriptional regulator	Up
5	BCG_0380	1.59	3.65	Rv0341	*iniB*	isoniazid inducible gene protein iniB	
	BCG_0381	2.70	5.41	Rv0342	*iniA*	isoniazid inducible gene protein iniA	
	BCG_0382	1.34	4.04	Rv0343	*iniC*	isoniazid inducible gene protein iniC	Dn
6	BCG_0479	5.50	1.90	Rv0440	*groEL*	chaperonin GroEL	Up
7	BCG_0741	−1.94	−1.36	Rv0692		hypothetical protein	
	BCG_0742	−1.84	−2.06	Rv0693	*pqqE*	coenzyme PQQ synthesis protein E pqqE	
	BCG_0743	−2.18	−2.25	Rv0694	*lldD1*	L-lactate dehydrogenase (cytochrome) lldD1	NA
	BCG_0744	−1.09	−1.25	Rv0695		hypothetical protein	
	BCG_0745	−1.25	−1.43	Rv0696		membrane sugar transferase	
8	BCG_0755	1.24	1.42	Rv0705	*rpsS*	30S ribosomal protein S19	
	BCG_0756	1.31	1.12	Rv0706	*rplV*	50S ribosomal protein L22	
	BCG_0757	1.60	1.72	Rv0707	*rpsC*	30S ribosomal protein S3	
	BCG_0758	1.60	1.83	Rv0708	*rplP*	50S ribosomal protein L16	
	BCG_0759	1.12	1.30	Rv0709	*rpmC*	50S ribosomal protein L29	Up
	BCG_0760	1.79	1.94	Rv0710	*rpsQ*	30S ribosomal protein S17	
9	BCG_0772	1.64	1.52	Rv0722	*rpmD*	50S ribosomal protein L30	NA
	BCG_0773	1.17		Rv0723	*rplO*	50S ribosomal protein L15	
10	BCG_1025c	−1.22	−2.25	Rv0971c	*echA7*	enoyl-CoA hydratase	
	BCG_1026c	−1.64	−2.74	Rv0972c	*fadE12*	acyl-CoA dehydrogenase fadE12	
	BCG_1027c	−1.15	−2.06	Rv0973c	*accA2*	acetyl-/propionyl-coenzyme A carboxylase alpha chain subunit alpha accA2	Up
	BCG_1028c	−1.00	−2.47	Rv0974c	*accD2*	acetyl-/propionyl-coa carboxylase subunit beta accD2	
11	BCG_1191	−6.64	−1.40	Rv1130		hypothetical protein	
	BCG_1192	−5.64	−1.51	Rv1131	*gltA1*	citrate synthase	NA
	BCG_1193	−2.00	−1.36	Rv1132		hypothetical protein	
12	BCG_1214c	1.38	1.06	Rv1153c	*omt*	O-methyltransferase omt	NA
13	BCG_1223	−1.69	−2.32	Rv1161	*narG*	respiratory nitrate reductase (alpha chain) narG	
	BCG_1224	−1.12	−1.64	Rv1162	*narH*	respiratory nitrate reductase subunit beta narH	Up
	BCG_1225	−1.06	−1.22	Rv1163	*narJ*	respiratory nitrate reductase (delta chain) narJ	
14	BCG_1249		−1.06		Rv1187	rocA	
	BCG_1250		−1.40	Rv1188			Up
	BCG_1251	−2.06	−2.25	Rv1189	*sigI*	RNA polymerase sigma factor SigI	
15	BCG_1692	2.18		Rv1653	*argJ*	bifunctional ornithine acetyltransferase/N-acetylglutamate synthase	
	BCG_1693	2.34		Rv1654	*argB*	acetylglutamate kinase	
	BCG_1694	2.34		Rv1655	*argD*	acetylornithine aminotransferase	NA
	BCG_1695	1.32		Rv1656	*argF*	ornithine carbamoyltransferase	
	BCG_1696	1.13		Rv1657	*argR*	arginine repressor	
16	BCG_2047c	−1.94	−2.94	Rv2028c		hypothetical protein	NA
	BCG_2048c	−1.84	−2.64	Rv2029c	*pfkB*	phosphofructokinase pfkB	
	BCG_2049c	−1.18	−2.18	Rv2030c		hypothetical protein	
	BCG_2050c	−1.84	−1.94	Rv2031c	*hspX*	heat shock protein hspX	
17	BCG_2264	1.04	1.94	Rv2247	*accD6*	acetyl/propionyl CoA carboxylase subunit beta	NA
	BCG_2265	1.71	2.98	Rv2248		hypothetical protein	
	BCG_2266	1.79	3.04	Rv2248		hypothetical protein	
	BCG_2267c	2.28		Rv2249c	*glpD1*	glycerol-3-phosphate dehydrogenase glpd1	
	BCG_2268c	2.11		Rv2250c		transcriptional regulatory protein	
	BCG_2269	2.16		Rv2251		flavoprotein	
	BCG_2270	2.20		Rv2252		diacylglycerol kinase	
	BCG_2271	−1.36	−1.47	Rv2253		hypothetical protein	
18	BCG_2651c	−1.69	−2.18	Rv2624c		hypothetical protein	
	BCG_2652c	−2.00	−2.32	Rv2625c		transmembrane alanine and leucine rich protein	
	BCG_2653c	−1.84		Rv2626c		hypothetical protein	Up
	BCG_2654c	−1.84	−1.03	Rv2627c		hypothetical protein	
	BCG_2655	−2.40	−1.36	Rv2628		hypothetical protein	
	BCG_2656	−1.29	−1.56	Rv2629		hypothetical protein	
	BCG_2657	−1.22	−1.74	Rv2630		hypothetical protein	
	BCG_2658	−1.15	−1.36	Rv2631		hypothetical protein	
19	BCG_3155c	−1.69	−2.18	Rv3132c	*devS*	two component sensor histidine kinase devS	Up
	BCG_3156c	−1.56	−1.94	Rv3133c	*devR*	two component transcriptional regulatory protein DevR	
	BCG_3157c	−1.84	−2.32	Rv3134c		hypothetical protein	
20	BCG_3520c	2.08	1.96	Rv3455c	*truA*	tRNA pseudouridine synthase A	Up
	BCG_3521c	1.19		Rv3456c	*rplQ*	50S ribosomal protein L17	
	BCG_3522c	1.44	1.53	Rv3457c	*rpoA*	DNA-directed RNA polymerase subunit alpha	

^*^log 2 transformed expression values in microarray analysis.

^**^ChIP-seq peaks identified in *inbR* overexpressed strain. Up, upstream of the operon or gene; Dn, downstream of the operon/gene; In, inside of a gene; NA, peak is not available. Peaks are visualized in Figure 5 and Figure S7.
