# Supplementary material for: InbR, a TetR family regulator, binds with isoniazid and influences multidrug resistance in Mycobacterium bovis BCG
Source: Sci Rep. 2015 Sep 10;5:13969. doi: 10.1038/srep13969 (PMC4564863; doi:10.1038/srep13969)
Supplement: Supplementary Information [file srep13969-s1.pdf]

InbR, a TetR family regulator, binds with isoniazid and influences multidrug resistance in *Mycobacterium bovis* BCG

Min Yang[1], Chun-Hui Gao[2], Jialing Hu[1], Lei Zhao[1], Qiaoyun Huang[1], Zheng-Guo He[1]*

[1]National Key Laboratory of Agricultural Microbiology, Center for Proteomics Research, College of Life Science and Technology, Huazhong Agricultural University, Wuhan 430070, China

[2]School of Life Sciences and CAS Key Laboratory of Innate Immunity and Chronic Disease, University of Science and Technology of China, Hefei, Anhui 230027, China

*To whom correspondence should be addressed:

College of Life Science and Technology, Huazhong Agricultural University, Wuhan 430070, China

E-mail: he.zhengguo@hotmail.com or hezhengguo@mail.hzau.edu.cn

Tel: +86-27-87284300, Fax: +86-27-87280670

Supplementary method

β-galactosidase assay

The HindIII/NheI fragment containing the *LacZ* gene was amplified from pGOAL17 by PCR using the primers LacZf and LacZr and was cloned into pMV261[34] digested with HindIII/NheI to create pMV261LacZ (YZ0) (Table S4). The DNA fragment containing 730 bp upstream of the annotated start codon of the transcription factor along with the open reading frames (ORFs) was amplified from *M. tuberculosis* H37Rv genomic DNA by PCR using specific primers. The fragments were digested with corresponding restriction enzyme pairs and cloned into pMV261LacZ digested with the same enzymes. The resulting plasmids were transformed into *E. coli* DH5α to obtain the corresponding reporter strains YZ2 and YZ3. β-galactosidase activity of these recombinant *E. coli* strains were examined. All of the strains were grown in LB-Kanamycin (Kan) medium at 37 ° C for 16 h. Subsequently, a part of the cell suspension was inoculated into LB broth containing 30 μg/ml Kan liquid medium and grown at 37 ° C to OD_{600} of 0.5 to 0.8. β-Galactosidase measurements were performed as previously described[36]. The remaining cell suspensions were plated on LB agar containing 30 μg/ml Kan and 50 μg/ml X-gal, and grown at 37 ° C. Images were acquired using a digital camera.

Fig. S1

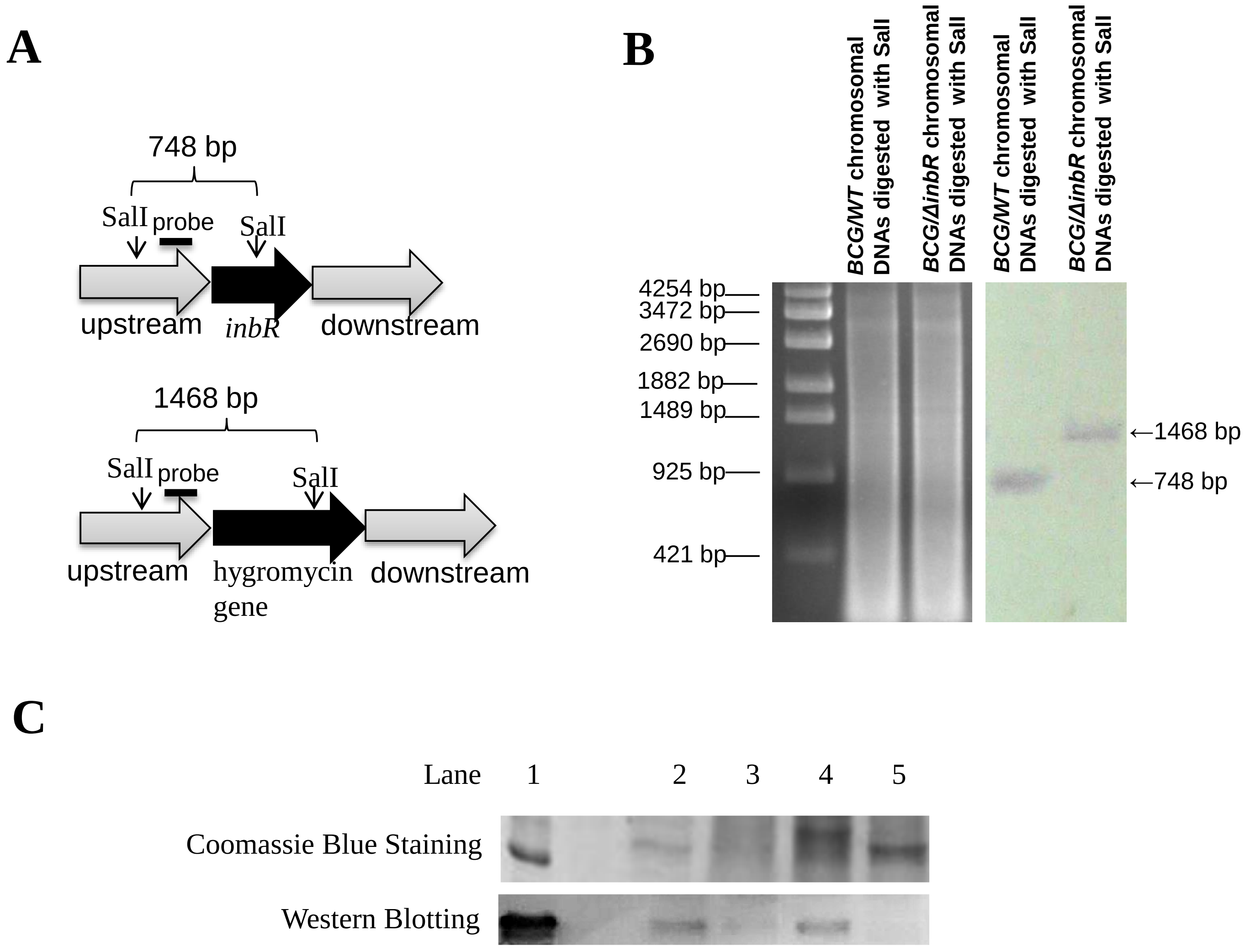

Construction of *inbR*-deleted strain. we used a gene replacement strategy and produced an inbR-deleted mutant *M. bovis* BCG strain (A). Deletion of inbR in the *M. bovis* BCG ΔinbR strain was confirmed by Southern blot assay (B). A single band approximately 1.4 kb in size was detected using a 270 bp probe from the SalI-digested genomic DNA of the mutant BCG strain. By contrast, a single band of only about 0.7 kb was observed in the wild-type strain (B). This finding was consistent with the expected band sizes that could be obtained upon replacement of the InbR gene with the hygromycinr gene, which indicated that the *inbR* gene was successfully deleted in the mutant strain. (C). Western Blotting assays. Lane 1, his-tagged InbR, lane 2, BCG/ΔinbR-complemented, lane 3, BCG/pMV261, lane 4, BCG/pMV-inbR, lane 5, BCG/ΔinbR. Coomassie Blue Staining and Western Blotting results were shown in top panel and bottom panel, respectively.

Fig. S2

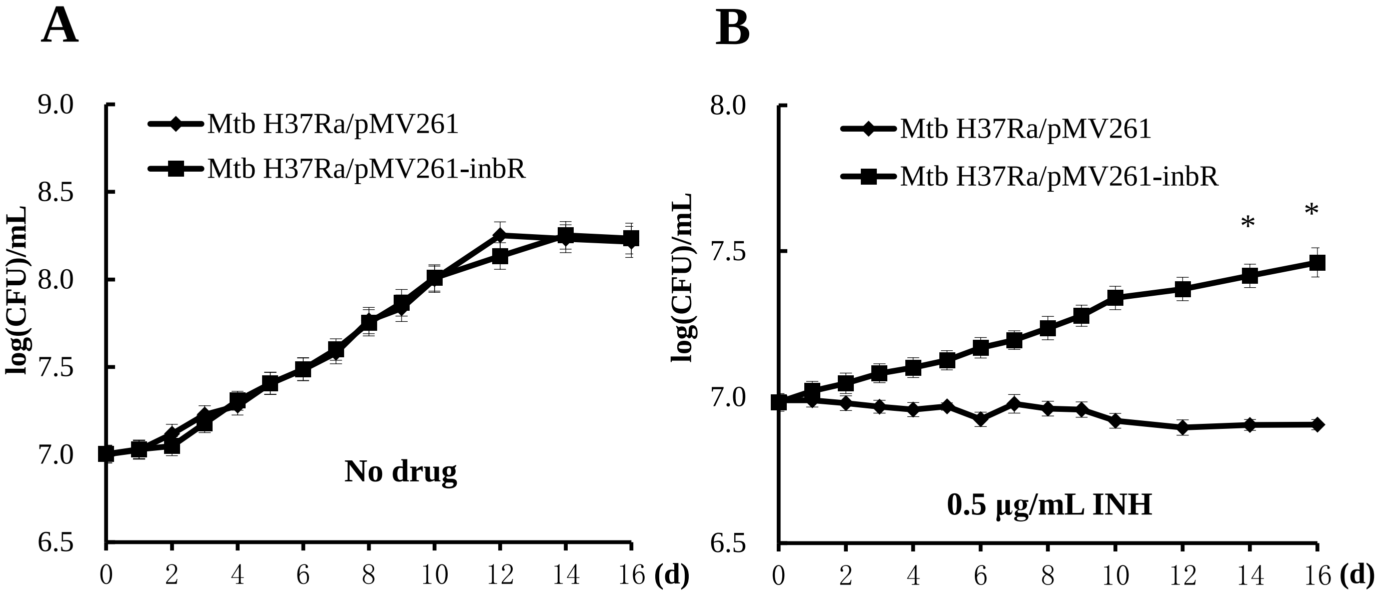

Effect of InbR on the growth of recombinant *M. tuberculosis* H37Ra. Growth curve of pMV261 (control) and pMV261-inbR (inbR overexpressed) strains in the absence (A) or presence (B) of 0.5µg/ml INH. Error bars represent the standard deviation across three biological replicates. The *p* values of the relative growth data were calculated by unpaired two-tailed Student's t-test using GraphPad Prism 5. The *p*-values of the results at two time points, 14 d and 16 d, were <0.05, which are indicated by asterisks (*).

Fig. S3

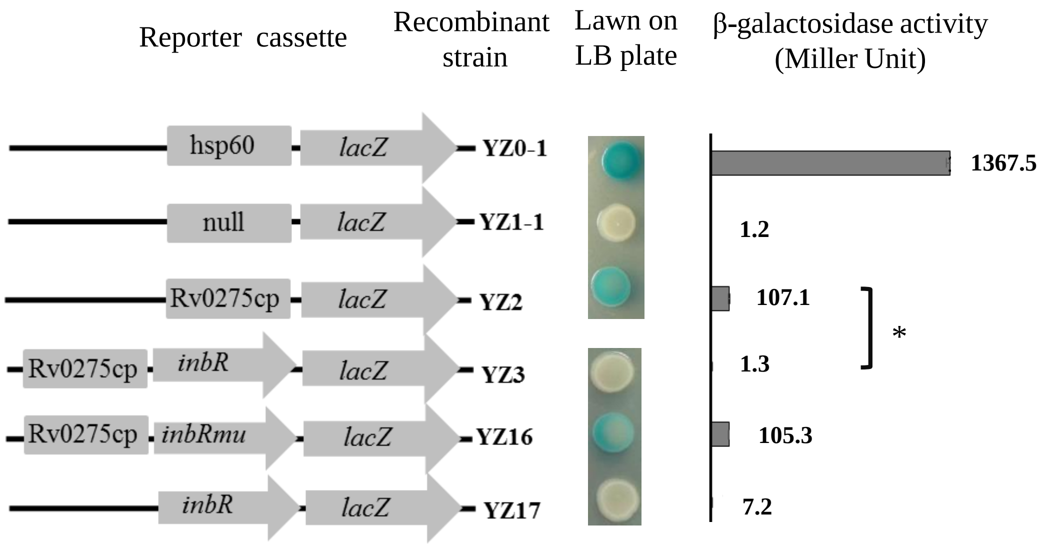

β-Galactosidase activity assays for auto-regulation of InbR. Left column Schematic representation of each clone used to generate reporter strains. Null promoter-*lacZ* and *hsp60-lacZ* were used as controls. Middle column: exponentially growing *E. coli* DH5α cultures of reporter strains were scribed onto LB plates containing 30 μg/ml Kan and 50 μg/ml X-gal. The plates were incubated subsequently for 12 h. Right column: β-galactosidase activity is expressed as Miller units.

Since there is an InbR homolog in *Mycobacterium smegmatis*, we performed the assays in *E. coli* DH5α, to avoid possible unwanted interplays between InbR and/or its homolog. The strain YZ0-1 was deep blue in color and showed high β-galactosidase concentration (~1367 Miller units), while the strain YZ1-1 was light blue in color. Several constructs containing Rv0275cp (YZ2), Rv0275cp + InbR (YZ3), InbR (MY17), or Rv0275cp + InbRmu, which lacks the start codon of InbR coding sequence and therefore is not able to be translated into a functional protein, were also applied to promote the expression of *lacZ*. The promoter Rv0275cp promoted the expression of *lacZ* (YZ2). By contrast, expression was repressed to levels observed in the promoterless *lacZ* construct in the presence of InbR (YZ3). Additionally, the repression was not observed in the presence of InbRmu (YZ16), while coding sequence of InbR lacks promoter activity (YZ17).

Fig. S4

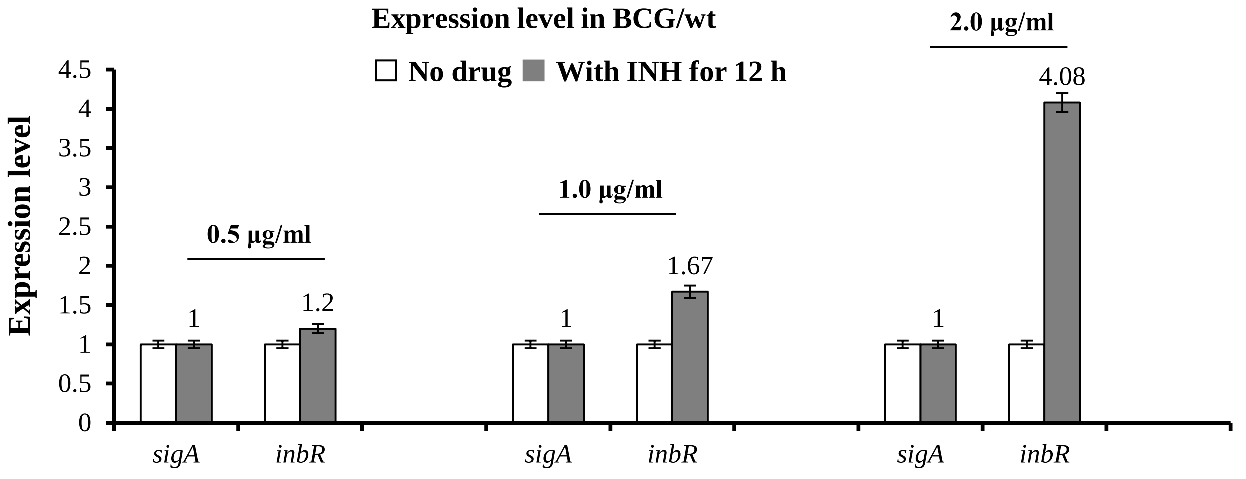

qRT-PCR assays for InbR expression patterns under various INH concentrations. The relative expression levels of the genes were normalized using *sigA* mRNA as an invariant transcript. Data were analyzed using the $2^{\triangle\triangle Ct}$ method. The *p*-values of the relative expression data were calculated by unpaired two-tailed Student's.

Fig. S5

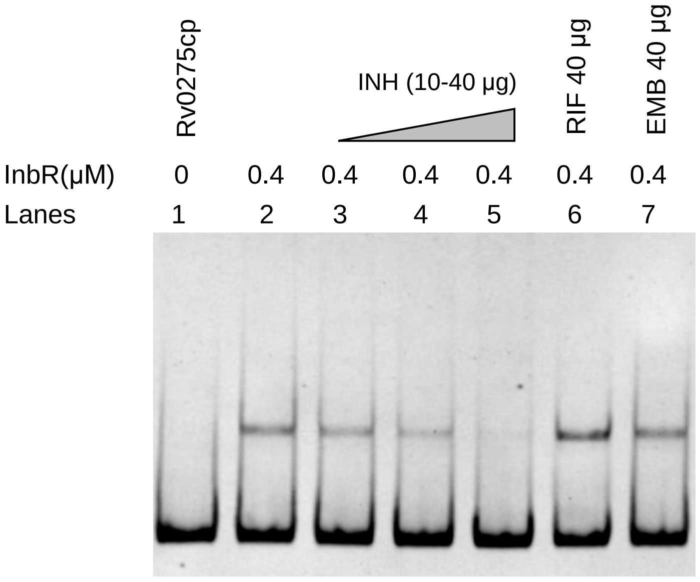

Comparison of INH, RIF and EMB on the DNA-binding activity of InbR.

Fig. S6

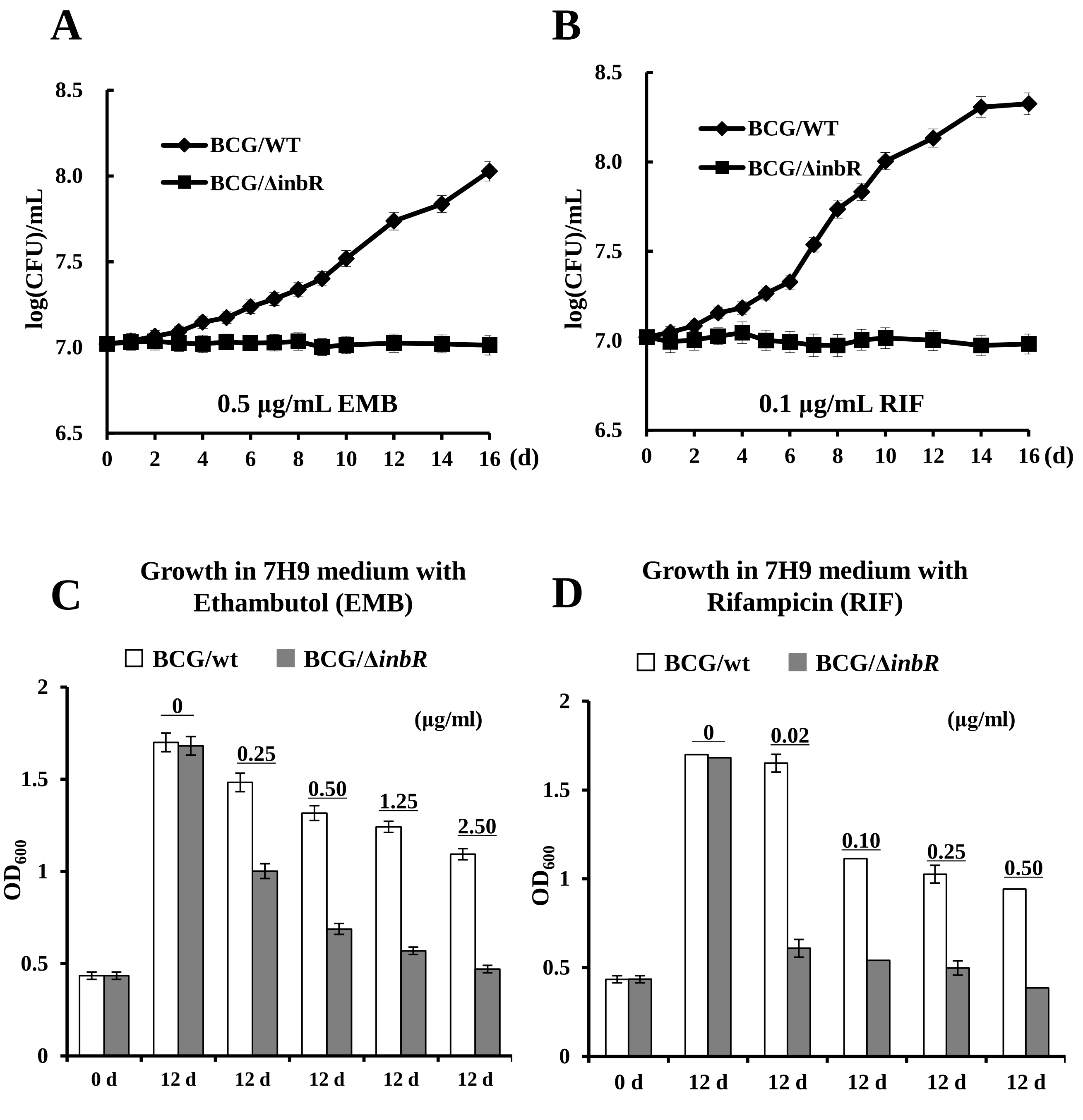

Assays for the effects of InbR on EMB and RIF resistance in *M. bovis* BCG. Growth curves of wild type and *inbR*-deleted strain in the presence of EMB (A) and RIF (B). Growth of strains in different concentrations of EMB (C) and (D). Drug concentrations were indicated in each figure. Error bars represent the standard deviation across three biological replicates.

Fig. S7

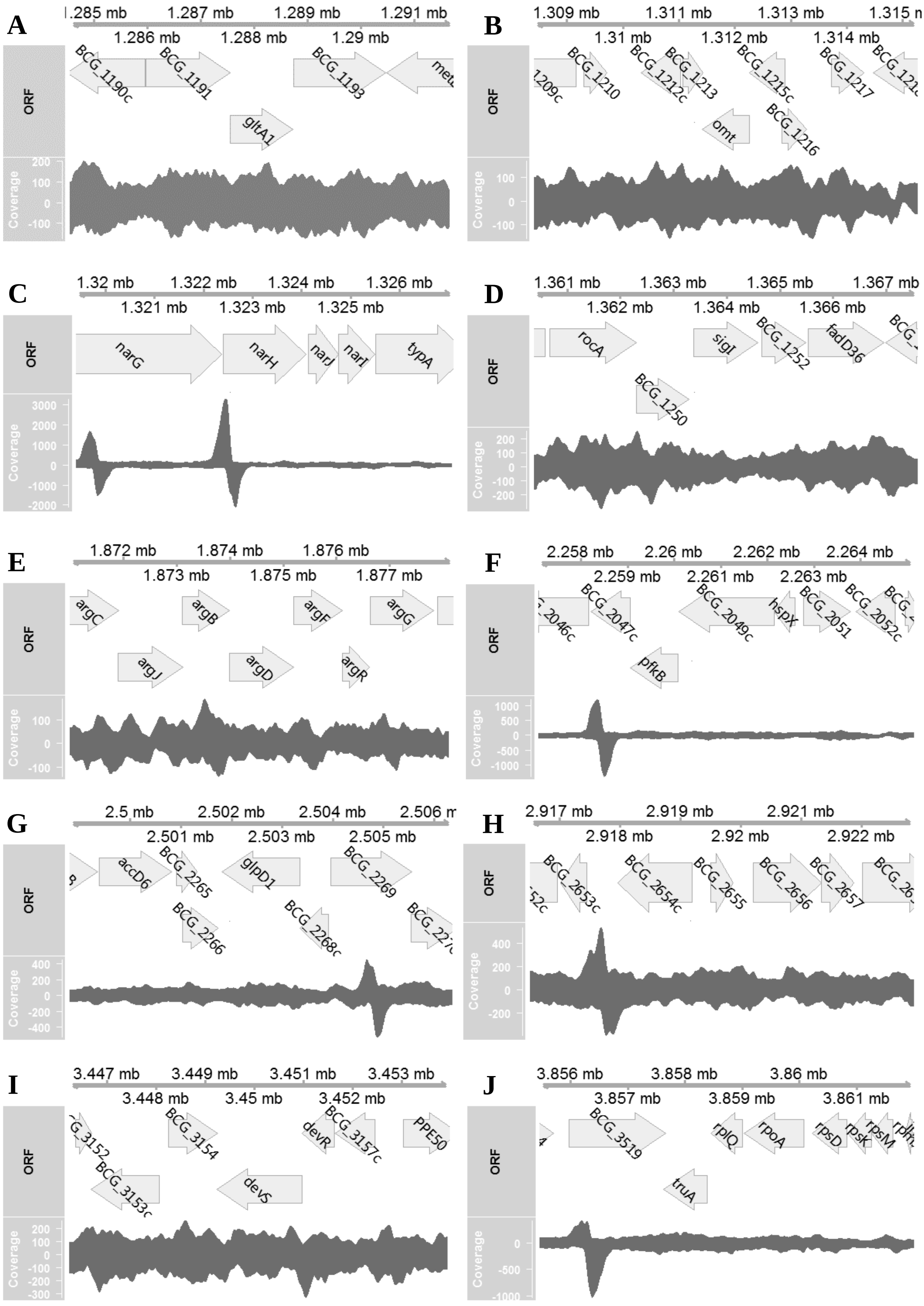

ChIP-seq assay. This figure is a supplement to Fig. 5.

Table S1 Primers used in this study

Name	Sequence 5'–3'	Enzyme	Usage
Rv0275cf	ATAA GCGGCCGC A ATGACGCGCAGTGATCGACC	NotI	Clone and expression
Rv0275cr	CAGA TCTAGA AGA CTACGAGCGACGCGCAGGCC	XbaI	Clone and expression
Rv0275cmuf	ATAA GCGGCCGC A CATACGCGCAGTGATCGACC	NotI	Clone to pMV261
Rv0135cf	ATAAT GCGGCCGC T GTGACAGCAGTCGCCGCCGG	NotI	Clone and expression
Rv0135cr	CGACG TCTAGA CCG CTAGCTGCGGGGACCGAGCA	XbaI	Clone and expression
BCG_0313cUPf	CAGC TTAATTAA TCATCCGCCGGTCCAGGTCC	PacI	Knock out
BCG_0313cUPr	CAGC ACTAGT ACGGATCATCTTGACACTCG	SpeI	Knock out
BCG_0313cDNf	GCGC AAGCTT TGAATTCCTTTGTCCAGCAA	HindIII	Knock out
BCG_0313cDNr	GCGC GCTAGC TATTGGAATCTGGCATATTT	NheI	Knock out
Rv0275cpLf	AAGCGGCCGCGGATACAGGTAGTCGCGAAC	NotI	Clone to pMV261
Rv0275cpLr	CGGGTCTAGAACGGATCATCTTGACACTCG	XbaI	Clone to pMV261
LacZf	ATTCAAGCTTATGAGGATGAGGGAAGCAAG	HindIII	Clone to pMV261
LacZr	ATGCGCTAGCTTATTTTTGACACCAGACCA	NheI	Clone to pMV261
Rv0275cpf	ATATGAATTCGGTCCCAGCGGTGCGACGAT	NotI	Clone and ChIP
Rv0275cpr	GCGCTCTAGAGTCGAGGTCTTTCAGATGGA	XbaI	Clone and ChIP
Rv3430cpf	ATATGAATTCCGGGCGCAGCGGCGTACGTT	EcoRI	Clone and ChIP
Rv3430cpr	AGACTCTAGAGCTCAGCGCTCACTCTCGCA	XbaI	Clone and ChIP

Notes: Restriction enzyme sites are underlined.

Table S2 Strains and plasmids used in this study

Strain or plasmid	Relevant genotype or features	Source or reference
Strains		
E.coli		
DH5a	Host for plasmid construction	TaKaRa
BL21	Host for overexpression	TaKaRa
BCG/WT	M. bovis BCG	ATCC
BCG/pMV261	BCG with pMV261	This study
BCG/pMV261- *inbR*	BCG with pMV261::*inbR*	This study
BCG/△inbR	BCG_0313c replaced by hyg	This study
△*yibR* complementation	BCG/△*inbR* with pMind::*inbR*	This study
Ra/WT	M. tuberculosis H37Ra	ATCC
Ra/pMV261	H37Ra with pMV261	This study
Ra/pMV261- *inbR*	H37Ra with pMV261::*inbR*	This study
YZ0-1	DH5a with pMV261::LacZ	This study
YZ1-1	DH5a with pMV::GroEL1P::LacZ	This study
YZ2	DH5a with pMV::0275cP::LacZ	This study
YZ3	DH5a with pMV::0275cP::0275c::LacZ	This study
YZ16	DH5a with pMV:: 0275cP::0275cmu::LacZ	This study
YZ17	DH5a with pMV::0275c::LacZ	This study
Plasmids		This study
pMV261	Kan^r, pAL5000 replicon	This study
pMV261::*inbR*	*inbR* in NotI-XbaI site of pMV261	This study
pMV261:: LacZ	LacZ in HindIII-NheI site of pMV261	This study
pMV261::GroEL1P::LacZ	GroEL1P in EcoRI-HindIIIsite of pMV261::LacZ	This study
pMV261::0275cP::LacZ	Rv0275cP in NotI-XbaI site of pMV261::LacZ	This study
pMV261:: 0275c::LacZ	Rv0275c in NotI-XbaI site of pMV261::LacZ	This study
pMV261:: 0275c::0275cP::LacZ	Rv0275cP+ORF in NotI -XbaI site of pMV261::LacZ	This study
pMV261:: 0275cmu::0275cP::LacZ	Rv0275cP+ORFmu in NotI -XbaI site of pMV261::LacZ	This study
pMind	Kan^r, pAL5000 replicon	This study
pMind-*inbR*	*inbR* in NotI-XbaI sites of pMind	This study
pET28a(+)	Kanr, T7 lac promoter, N-terminal His6	This study
pET- inbR	*inbR* in NotI-XbaI sites of pET28a	This study
pET- Rv0135c	Rv0135c in NotI-XbaI sites of pET28a	

Table S3 DNA substrate fragment used in this study

Name	Length and Source	Sequence or primers used to amplify long segments 5'–3'
Rv0275p	350bp, PCR	f: ATATGAATTCGGTCCCAGCGGTGCGACGAT
		r: GCGCTCTAGAGTCGAGGTCTTTCAGATGGA
Rv3430cp	350bp, PCR	f: ATATGAATTCCGGGCGCAGCGGCGTACGTT
		r: AGACTCTAGAGCTCAGCGCTCACTCTCGCA
Rv0275cp-$_{FITC}$	420bp, PCR	f: TGGCCAGCTGGAGACGGGGTGGGT labeled with FITC
		r: ACACCAGCGACGGCGAAGCAAGCA
Rv0275cp-$_{Biotin}$	420bp, PCR	f: TGGCCAGCTGGAGACGGGGTGGGT labeled with Biotin
		r: ACACCAGCGACGGCGAAGCAAGCA

Table S4 Primers for Reverse transcription -PCR

Name	Sequence 5'–3'	Usage
RT-*inbR*f	CAAATGACCACGTCAAGGCG	Reverse transcription -PCR
RT-*inbR*r	GACGGGACAAGTTTGGGTCG	Reverse transcription -PCR
RT-*iniB*f	GCATCGCTAGCCAGATCGGT	Reverse transcription -PCR
RT-*iniB*r	AGATGAGGCATAGCAGCGCC	Reverse transcription -PCR
RT-*iniA*f	CCGTCGATGACATCAGCACC	Reverse transcription -PCR
RT-*iniA*r	GAACCACATCTCGGGTTCGG	Reverse transcription -PCR
RT-*iniC*f	CGATTCTGCACGCAACCATC	Reverse transcription -PCR
RT-*iniC*r	GCCGGAACCAGGTCACAATC	Reverse transcription -PCR
RT-groEL2f	GCCGATGCGGTAAAGGTGAC	Reverse transcription -PCR
RT-groEL2r	GGCGACGTCATCGGTCTTCT	Reverse transcription -PCR
RT-BCG_0114f	TCGGTCGGTGAGTTGCTGTC	Reverse transcription -PCR
RT-BCG_0114r	GAGGTCCTCTAGCACCGCCA	Reverse transcription -PCR
RT-*dosR*f	AGGCCTTACCGACCAGGAGC	Reverse transcription -PCR
RT-*dosR*r	GCTTCAACTCCGTCGCGAAT	Reverse transcription -PCR
RT-groEL1f	ATTCCTGACACCGCCAAGGA	Reverse transcription -PCR
RT-groEL1r	CGTGCCGACAGGATCAGGTA	Reverse transcription -PCR
RT-BCG_3496cf	TGAGCACGTACTGGCGGTGC	Reverse transcription -PCR
RT-BCG_3496cr	GCTTGGTGATATCCCACGAC	Reverse transcription -PCR
RT-sigAf	TCGCGCCTACCTCAAACAG	Reverse transcription -PCR
RT-sigAr	CGTACAGGCCAGCCTCGAT	Reverse transcription -PCR

Table S5 Combined result of Microarrays and ChIP-seq

	ORF			Gene expression		ChIP-seq peaks				
No.	feature	Rv_gene	gene	FC_OE	FC_INH	Distance	position	qvalue	fold enrichment	description
1	BCG_0002	Rv0002	*dnaN_1*		1.10					D polymerase III subunit beta
2	BCG_0003	Rv0003	*recF*		1.25					recombination protein F
3	BCG_0004	Rv0004		1.02						hypothetical protein
4	BCG_0009	Rv0009	*ppiA_1*		1.06					iron-regulated peptidyl-prolyl cis-trans isomerase A ppiA
5	BCG_0029c	Rv3923c	*rnpA*		1.32					ribonuclease P
6	BCG_0030c	Rv3924c	*rpmH*		1.08					50S ribosomal protein L34
7	BCG_0031	Rv0001	*dnaA*			192	Up	1.6E+03	4.0	chromosomal replication initiation protein
8	BCG_0044c	Rv0014c	*pknB*			100	Up	1.2E+02	1.7	Ser/Thr protein kinase
9	BCG_0054	Rv0024		1.04						hypothetical protein
10	BCG_0058	Rv0027				103	Up	9.7E+01	1.6	hypothetical protein
11	BCG_0061	Rv0030			-1.03					hypothetical protein
12	BCG_0071c	Rv0040c	*mtc28*		1.44					hypothetical protein
13	BCG_0073c	Rv0042c				139	Up	2.1E+02	1.6	transcriptional regulatory protein (marR-family)
14	BCG_0076c	Rv0045c			1.66					hydrolase
15	BCG_0077c	Rv0046c	*ino1*		2.46	114	Up	1.6E+04	8.9	myo-inositol-1-phosphate synthase INO1
16	BCG_0078c	Rv0047c			1.83	174	Up	1.6E+04	8.9	hypothetical protein
17	BCG_0079c	Rv0048c		1.01	1.24					hypothetical protein
18	BCG_0083	Rv0052			-1.09					hypothetical protein
19	BCG_0084	Rv0053	*rpsF*		1.26					30S ribosomal protein S6
20	BCG_0085	Rv0054	*ssb*	1.23						single-stranded D-binding protein
21	BCG_0086	Rv0055	*rpsR*	1.91	2.14	10	Up	1.9E+02	1.8	30S ribosomal protein S18
22	BCG_0087	Rv0056	*rplI*	1.52	1.53					50S ribosomal protein L9
23	BCG_0088	Rv0057		1.72	1.74					hypothetical protein
24	BCG_0092	Rv0061			1.83					hypothetical protein
25	BCG_0095	Rv0064				428	Up	1.1E+02	1.7	hypothetical protein
26	BCG_0100c	Rv0069c	*sdaA*			82	Up	1.1E+02	1.6	l-serine dehydratase sdaA
27	BCG_0104	Rv0073				181	Up	7.6E+02	2.8	glutamine ABC transporter ATP-binding protein
28	BCG_0105	Rv0074			-1.15					hypothetical protein
29	BCG_0106	Rv0075			-1.00					aminotransferase
30	BCG_0108c	Rv0077c		2.26						oxidoreductase
31	BCG_0112	Rv0079		-1.32		80	Up	2.8E+02	2.1	hypothetical protein
32	BCG_0113	Rv0080			-1.89					hypothetical protein
33	BCG_0114	Rv0081		-1.89	-1.56					transcriptional regulatory protein
34	BCG_0115	Rv0082		-1.36	-1.36					oxidoreductase
35	BCG_0116	Rv0083		-1.69	-1.25					oxidoreductase
36	BCG_0117	Rv0084	*hycD*	-1.09		33	Up	1.1E+02	1.6	formate hydrogenlyase hycD (FHL)
37	BCG_0118	Rv0085	*hycP*	-1.03						hydrogenase hycP
38	BCG_0119	Rv0086	*hycQ*			1	Up	4.7E+01	1.4	hydrogenase hycQ
39	BCG_0120	Rv0087	*hycE*		1.40	26	Up	5.9E+01	1.4	formate hydrogenase hycQ (FHL)
40	BCG_0121	Rv0088			-1.22	313	Up	5.9E+01	1.4	hypothetical protein
41	BCG_0122	Rv0089			-1.79	14	Up	2.1E+02	1.9	methyltransferase/methylase
42	BCG_0123	Rv0090				292	Up	2.1E+02	1.9	hypothetical protein
43	BCG_0132	Rv0099	*fadD10*			198	Up	6.5E+01	1.5	acyl-CoA synthetase
44	BCG_0134	Rv0101	*nrp*		-1.06					peptide synthetase nrp
45	BCG_0139	Rv0106			1.10					hypothetical protein
46	BCG_0140c	Rv0107c	*ctpI*			34	Up	2.4E+02	2.0	cation-transporter atpase I ctpI
47	BCG_0142	Rv0109	*PE_PGRS1*		-1.51					PE-PGRS family protein
48	BCG_0154c	Rv0120c	*fusA2b*		-1.56	81	Up	7.6E+01	1.5	elongation factor G
49	BCG_0155c	Rv0121c				216	Up	7.6E+01	1.5	hypothetical protein
50	BCG_0156	Rv0122			-1.43	119	Up	7.6E+01	1.5	hypothetical protein
51	BCG_0158	Rv0124	*PE_PGR*	-1.09	-1.29					PE-PGRS family protein
52	BCG_0160	Rv0126	*treS*		1.28	95	Up	5.6E+03	7.6	trehalose synthase treS
53	BCG_0163c	Rv0129c	*fbpC*		1.38					hypothetical protein
54	BCG_0174	Rv0138				59	Up	4.8E+02	2.5	hypothetical protein
55	BCG_0176	Rv0140		-1.09						hypothetical protein
56	BCG_0178	Rv0142			-1.15					hypothetical protein
57	BCG_0185	Rv0149				189	Up	1.3E+02	1.7	quinone oxidoreductase
58	BCG_0187c	Rv0151c	*PE1*	-1.00						PE family protein
59	BCG_0191	Rv0155	*pntAa*		1.31					D(p) transhydrogenase (subunit alpha) pntAa
60	BCG_0193	Rv0157	*pntB*	1.20						D(p) transhydrogenase (subunit beta) pntB
61	BCG_0196c	Rv0160c	*PE4*		-2.32					PE family protein
62	BCG_0200	Rv0164	*TB18.5*		1.10					hypothetical protein
63	BCG_0203	Rv0166	*fadD5*		1.04					long-chain-fatty-acid--CoA ligase
64	BCG_0206	Rv0169	*mce1A*		-1.09					MCE-family protein mce1A
65	BCG_0211	Rv0174	*mce1F*	-1.06	-1.51	159	Up	1.9E+03	4.3	MCE-family protein mce1F
66	BCG_0212	Rv0175				123	Up	1.9E+02	1.9	mce associated membrane protein
67	BCG_0216c	Rv0179c	*lprO*		2.21					lipoprotein lprO
68	BCG_0217c	Rv0180c				263	Up	1.0E+02	1.6	hypothetical protein
69	BCG_0222	Rv0185				70	Up	3.4E+01	1.3	hypothetical protein
70	BCG_0225	Rv0188				127	Up	5.2E+03	7.1	hypothetical protein
71	BCG_0226c	Rv0189c	*ilvD*		1.09					dihydroxy-acid dehydratase
72	BCG_0230c	Rv0193c				38	Up	6.6E+02	2.8	hypothetical protein
73	BCG_0231	Rv0194				86	Up	6.6E+02	2.8	drugs-transport transmembrane ATP-binding protein ABC transporter
74	BCG_0245c	Rv0208c	*trmB*		1.48					tR (guanine-N(7)-)-methyltransferase
75	BCG_0247	Rv0210			1.08					hypothetical protein

	ORF			Gene expression		ChIP-seq peaks				
No.	feature	Rv_gene	gene	FC_OE	FC_INH	Distance	position	qvalue	fold enrichment	description
76	BCG_0252c	Rv0215c	*fadE3*		-1.00					acyl-CoA dehydrogenase fadE3
77	BCG_0261c	Rv0224c				234	Up	5.1E+01	1.4	methyltransferase
78	BCG_0262	Rv0225				246	Up	5.1E+01	1.4	hypothetical protein
79	BCG_0264c	Rv0227c			1.64					hypothetical protein
80	BCG_0267c	Rv0230c	*php*			3	Up	2.0E+02	1.9	phosphotriesterase PHP
81	BCG_0268	Rv0231	*fadE4*			97	Up	2.0E+02	1.9	acyl-CoA dehydrogenase fadE4
82	BCG_0271c	Rv0234c	*gabD1*			20	Up	1.6E+02	1.8	succinate-semialdehyde dehydrogenase
83	BCG_0279c	Rv0241c		1.12	1.62	94	Up	2.8E+01	1.3	hypothetical protein
84	BCG_0281	Rv0243	*fadA2*		1.16					acetyl-CoA acetyltransferase
85	BCG_0285c	Rv0247c			1.09					fumarate reductase iron-sulfur subunit
86	BCG_0288c	Rv0250c		1.10						hypothetical protein
87	BCG_0289c	Rv0251c	*hsp*	1.20						heat shock protein hsp
88	BCG_0290	Rv0252	*nirB*	-2.84	-3.64					nitrite reductase D(P)H] large subunit
89	BCG_0291	Rv0253	*nirD*	-2.94	-3.47					nitrite reductase [D(P)H] small subunit nirD
90	BCG_0294c	Rv0256c	*PPE2*		1.08					PPE family protein
91	BCG_0296c	Rv0258c			-1.74	63	Up	1.6E+02	1.7	hypothetical protein
92	BCG_0297c	Rv0259c		-2.00	-3.32	87	Up	1.6E+02	1.7	hypothetical protein
93	BCG_0298c	Rv0260c			-2.18					bifunctional uroporphyrinogen-III synthetase/response regulator domain-containing protein
94	BCG_0299c	Rv0261c	*narK3*	-1.40	-3.06					integral membrane nitrite extrusion protein narK3
95	BCG_0300c	Rv0262c	*aac*		-1.09	46	Up	2.0E+02	1.9	aminoglycoside 2'-n-acetyltransferase aac
96	BCG_0301c	Rv0263c			-1.40					hypothetical protein
97	BCG_0302c	Rv0264c			-1.32					hypothetical protein
98	BCG_0303c	Rv0265c		1.34						periplasmic iron-transport lipoprotein
99	BCG_0305	Rv0267	*narU*	-2.12	-3.84					integral membrane nitrite extrusion protein narU
100	BCG_0307c	Rv0269c			-1.00					hypothetical protein
101	BCG_0309c	Rv0271c	*fadE6*	1.02						acyl-CoA dehydrogenase fadE6
102	BCG_0313c	Rv0275c		7.55		302	Up	7.0E+05	28.5	TetR family transcriptional regulator
103	BCG_0314	Rv0276				276	Up	7.0E+05	28.5	hypothetical protein
104	BCG_0317c	Rv0278c	*PE_PGRS3a*		-1.36					PE-PGRS family protein
105	BCG_0318c	Rv0278c	*PE_PGRS3*		-1.69					PE-PGRS family protein
106	BCG_0321	Rv0281			1.29					hypothetical protein
107	BCG_0322	Rv0282			2.04					hypothetical protein
108	BCG_0323	Rv0283			1.91					hypothetical protein
109	BCG_0324	Rv0284			2.17	12	Up	6.0E+01	1.5	hypothetical protein
110	BCG_0325	Rv0285	*PE5*		1.45					PE family protein
111	BCG_0326	Rv0286	*PPE4*		1.94					PPE family protein
112	BCG_0327	Rv0287	*esxG*		1.73					hypothetical protein
113	BCG_0328	Rv0288	*esxH*		1.69	94	Up	2.5E+02	2.0	low molecular weight protein antigen 7 cfp7
114	BCG_0329	Rv0289			1.34	260	Up	2.5E+02	2.0	hypothetical protein
115	BCG_0332	Rv0292			1.29					hypothetical protein
116	BCG_0335c	Rv0295c				56	Up	1.7E+01	1.2	hypothetical protein
117	BCG_0337	Rv0297	*PE_PGRS5*		-1.12					PE-PGRS family protein
118	BCG_0339	Rv0299			-1.00					hypothetical protein
119	BCG_0340	Rv0300		1.36						hypothetical protein
120	BCG_0343	Rv0303		1.20						dehydrogenase/reductase
121	BCG_0349	Rv0309			1.68	62	Up	5.4E+01	1.4	hypothetical protein
122	BCG_0350c	Rv0310c				124	Up	1.8E+01	1.2	hypothetical protein
123	BCG_0351	Rv0311				143	Up	1.8E+01	1.2	hypothetical protein
124	BCG_0352	Rv0312			2.33	518	Up	1.2E+02	1.7	hypothetical protein
125	BCG_0355	Rv0315			1.12					beta-1,3-glucanase
126	BCG_0356	Rv0316		1.20	1.01					muconolactone isomerase
127	BCG_0360	Rv0320			-2.64					hypothetical protein
128	BCG_0362	Rv0322	*udgA*	-1.25						udp-glucose 6-dehydrogenase udgA
129	BCG_0379	Rv0340			1.42					hypothetical protein
130	BCG_0380	Rv0341	*iniB*	1.59	3.65					isoniazid inductible gene protein iniB
131	BCG_0381	Rv0342	*iniA*	2.70	5.41					isoniazid inductible gene protein iniA
132	BCG_0382	Rv0343	*iniC*	1.34	4.04					isoniazid inductible gene protein iniC
133	BCG_0383c	Rv0344c	*lpqJ*			24	Up	1.4E+05	24.9	lipoprotein lpqJ
134	BCG_0389	Rv0350	*dnaK*	2.19	1.49					molecular chaperone DnaK
135	BCG_0390	Rv0351	*grpE*	2.28						heat shock protein GrpE
136	BCG_0391	Rv0352	*dnaJ1*	1.52						chaperone protein DnaJ
137	BCG_0392	Rv0353	*hspR*	1.38						heat shock protein transcriptional regulator HspR
138	BCG_0404c	Rv0366c			1.08					hypothetical protein
139	BCG_0406c	Rv0368c			-1.25					hypothetical protein
140	BCG_0407c	Rv0369c			-1.32					membrane oxidoreductase
141	BCG_0408c	Rv0370c			-1.64	1	Up	1.1E+02	1.6	oxidoreductase
142	BCG_0409c	Rv0371c			-2.18	224	Up	1.1E+02	1.6	hypothetical protein
143	BCG_0410c	Rv0372c			-2.12	341	Up	1.5E+02	1.7	hypothetical protein
144	BCG_0411c	Rv0373c			-2.32					carbon monoxyde dehydrogenase large chain
145	BCG_0412c	Rv0374c			-1.29					carbon monoxyde dehydrogenase small chain
146	BCG_0413c	Rv0375c			-1.32					carbon monoxyde dehydrogenase medium chain
147	BCG_0415	Rv0377			-1.03					LysR family transcriptional regulator
148	BCG_0423	Rv0385			1.04					monooxygenase
149	BCG_0424	Rv0386		1.10						LuxR family transcriptional regulator
150	BCG_0430	Rv0393				1	Up	1.6E+02	1.8	hypothetical protein
151	BCG_0438	Rv0401			-1.00					hypothetical protein
152	BCG_0440c	Rv0402c	*mmpL1a*			28	Up	1.5E+01	1.2	transmembrane transport protein mmpL1a
153	BCG_0441c	Rv0403c	*mmpS1*	-1.18	-1.09					membrane protein mmpS1

	ORF			Gene expression		ChIP-seq peaks				
**No.**	**feature**	**Rv_gene**	**gene**	**FC_OE**	**FC_INH**	**Distance**	**position**	**qvalue**	**fold enrichment**	**description**
154	BCG_0443	Rv0405	*pks6a*		-1.22					membrane bound polyketide synthase pks6a
155	BCG_0448	Rv0409	*ackA*		-1.22	517	Up	6.2E+01	1.5	acetate kinase
156	BCG_0450c	Rv0411c	*glnH*		1.33					glutamine-binding lipoprotein glnH
157	BCG_0451c	Rv0412c		1.42	2.04					hypothetical protein
158	BCG_0455	Rv0416	*thiS*			51	Up	1.2E+02	1.7	sulfur carrier protein ThiS
159	BCG_0457	Rv0418	*lpqL*	1.01	1.04					lipoprotein aminopeptidase lpqL
160	BCG_0460c	Rv0421c				225	Up	1.2E+02	1.7	hypothetical protein
161	BCG_0461c	Rv0422c	*thiD*			71	Up	6.7E+01	1.5	phosphomethylpyrimidine kinase
162	BCG_0469	Rv0430			-1.09					hypothetical protein
163	BCG_0478c	Rv0439c				314	Up	4.7E+03	6.8	short chain dehydrogenase
164	BCG_0479	Rv0440	*groEL*	5.50	1.90	229	Up	4.7E+03	6.8	chaperonin GroEL
165	BCG_0485c	Rv0446c		-1.15						hypothetical protein
166	BCG_0486c	Rv0447c	*ufaA1*	-1.84	-1.18					cyclopropane-fatty-acyl-phospholipid synthase ufaA1
167	BCG_0487c	Rv0448c		-1.15						hypothetical protein
168	BCG_0499	Rv0459			-1.40					hypothetical protein
169	BCG_0506	Rv0466			1.61					hypothetical protein
170	BCG_0507	Rv0467	*icl*	-2.06						isocitrate lyase icl
171	BCG_0508	Rv0468	*fadB2*		1.53					3-hydroxybutyryl-CoA dehydrogenase
172	BCG_0510c	Rv0470c	*pcaA*			168	Up	2.8E+02	2.1	mycolic acid synthase PcaA
173	BCG_0531	Rv0490	*senX3*			91	Up	6.5E+02	2.7	two component sensor histidine kinase senX3
174	BCG_0544	Rv0501	*galE2*			14	Up	3.7E+01	1.4	udp-glucose 4-epimerase galE2
175	BCG_0549	Rv0506	*mmpS2*		-1.00					membrane protein mmpS2
176	BCG_0559c	Rv0516c		-2.47	-5.06					hypothetical protein
177	BCG_0562c	Rv0519c		-1.32		186	Up	1.8E+05	25.9	hypothetical protein
178	BCG_0563	Rv0520		-2.40	-1.64	128	Up	1.8E+05	25.9	methyltransferase/methylase
179	BCG_0564	Rv0521			-1.15					methyltransferase/methylase
180	BCG_0584	Rv0540		-1.06						hypothetical protein
181	BCG_0592c	Rv0548c	*menB*			43	Up	6.9E+01	1.5	naphthoate synthase
182	BCG_0601	Rv0556				80	Up	2.1E+02	1.9	hypothetical protein
183	BCG_0614	Rv0569		-1.89						hypothetical protein
184	BCG_0615	Rv0570	*nrdZ*	-1.43	-1.60					ribonucleoside-diphosphate reductase large subunit nrdZ
185	BCG_0616c	Rv0571c		-1.40	-1.56					hypothetical protein
186	BCG_0617c	Rv0572c		-1.94	-1.60					hypothetical protein
187	BCG_0618c	Rv0573c			-1.60					nicotinate phosphoribosyltransferase
188	BCG_0619c	Rv0574c			-1.43					hypothetical protein
189	BCG_0623c	Rv0578c	*PE_PGRS7*		-1.18					PE-PGRS family protein
190	BCG_0629	Rv0584			-1.06					hypothetical protein
191	BCG_0633	Rv0588	*yrbE2B*			30	Up	7.2E+01	1.5	integral membrane protein YrbE2b
192	BCG_0634	Rv0589	*mce2A*		1.10					mce-family protein mce2A
193	BCG_0636	Rv0591	*mce2C*			354	Up	5.5E+01	1.4	mce-family protein mce2C
194	BCG_0653	Rv0607				4	Up	2.0E+01	1.3	hypothetical protein
195	BCG_0658c	Rv0611c		-1.51	-1.18					hypothetical protein
196	BCG_0660c	Rv0613c		1.14	1.01					hypothetical protein
197	BCG_0667	Rv0621			-1.51					hypothetical protein
198	BCG_0668	Rv0622			-1.40					hypothetical protein
199	BCG_0675c	Rv0629c	*recD*			449	Up	2.1E+02	1.9	exonuclease V (alpha chain) recD
200	BCG_0681c	Rv0634c		1.41						glyoxalase II
201	BCG_0683	Rv0634B	*rpmG*		1.50					50S ribosomal protein L33
202	BCG_0684	Rv0635			1.30					(3R)-hydroxyacyl-ACP dehydratase subunit HadA
203	BCG_0695c	Rv0646c	*lipG*		1.15					lipase/esterase lipG
204	BCG_0700	Rv0651	*rplJ*		1.40					50S ribosomal protein L10
205	BCG_0701	Rv0652	*rplL*		1.70					50S ribosomal protein L7/L12
206	BCG_0702c	Rv0653c			1.66					TetR family transcriptional regulator
207	BCG_0711c	Rv0662c				153	Up	2.9E+02	2.1	hypothetical protein
208	BCG_0712	Rv0663	*atsD*			152	Up	2.9E+02	2.1	arylsulfatase atsD
209	BCG_0717	Rv0668	*rpoC*	1.45	1.24					D-directed R polymerase subunit beta'
210	BCG_0721	Rv0672	*fadE8*	-1.03						acyl-CoA dehydrogenase fadE8
211	BCG_0722	Rv0673	*echA4*	-1.03	-1.32					enoyl-CoA hydratase
212	BCG_0723	Rv0674				227	Up	8.8E+01	1.6	hypothetical protein
213	BCG_0724	Rv0675	*echA5*		-1.09					enoyl-CoA hydratase
214	BCG_0725c	Rv0676c	*mmpL5*		1.36	35	Up	1.2E+02	1.7	transmembrane transport protein mmpL5
215	BCG_0726c	Rv0677c	*mmpS5*		1.76					membrane protein mmpS5
216	BCG_0727	Rv0678			1.24					hypothetical protein
217	BCG_0728c	Rv0679c			1.58					hypothetical protein
218	BCG_0731	Rv0682	*rpsL*		1.36					30S ribosomal protein S12
219	BCG_0732	Rv0683	*rpsG*		1.58					30S ribosomal protein S7
220	BCG_0734	Rv0685	*tuf*		1.50					elongation factor Tu
221	BCG_0741	Rv0692		-1.94	-1.36					hypothetical protein
222	BCG_0742	Rv0693	*pqqE*	-1.84	-2.06					coenzyme PQQ synthesis protein E pqqE
223	BCG_0743	Rv0694	*lldD1*	-2.18	-2.25					L-lactate dehydrogenase (cytochrome) lldD1
224	BCG_0744	Rv0695		-1.09	-1.25					hypothetical protein
225	BCG_0745	Rv0696		-1.25	-1.43					membrane sugar transferase
226	BCG_0746	Rv0697				367	Up	5.1E+01	1.4	dehydrogenase
227	BCG_0748	Rv0698				99	Up	9.6E+01	1.6	hypothetical protein
228	BCG_0755	Rv0705	*rpsS*	1.24	1.42					30S ribosomal protein S19
229	BCG_0756	Rv0706	*rplV*	1.31	1.12					50S ribosomal protein L22
230	BCG_0757	Rv0707	*rpsC*	1.60	1.72					30S ribosomal protein S3
231	BCG_0758	Rv0708	*rplP*	1.60	1.83					50S ribosomal protein L16
232	BCG_0759	Rv0709	*rpmC*	1.12	1.30	204	Up	8.2E+03	9.3	50S ribosomal protein L29

	ORF			Gene expression		ChIP-seq peaks				
No.	feature	Rv_gene	gene	FC_OE	FC_INH	Distance	position	qvalue	fold enrichment	description
233	BCG_0760	Rv0710	*rpsQ*	1.79	1.94	178	Up	8.2E+03	9.3	30S ribosomal protein S17
234	BCG_0769	Rv0719	*rplF*		1.07					50S ribosomal protein L6
235	BCG_0772	Rv0722	*rpmD*	1.64	1.52					50S ribosomal protein L30
236	BCG_0773	Rv0723	*rplO*	1.17						50S ribosomal protein L15
237	BCG_0776c	Rv0726c			1.49					hypothetical protein
238	BCG_0782	Rv0732	*secY*		1.56					preprotein translocase subunit SecY
239	BCG_0788	Rv0738		-1.47						hypothetical protein
240	BCG_0791	Rv0741			-1.32					transposase
241	BCG_0796	Rv0746	*PE_PGRS9*		-1.43					PE-PGRS family protein
242	BCG_0797	Rv0747	*PE_PGRS10*		-1.22					PE-PGRS family protein
243	BCG_0802c	Rv0751c	*mmsB*			291	Up	2.0E+02	1.9	3-hydroxyisobutyrate dehydrogenase
244	BCG_0804c	Rv0753c	*mmsA*		1.20	72	Up	9.5E+01	1.6	methylmalonate-semialdehyde dehydrogenase mmsA
245	BCG_0813c	Rv0761c	*adhB*	1.23	1.03					zinc-containing alcohol dehydrogenaseAdhB
246	BCG_0816c	Rv0764c	*cyp51*			254	Up	1.0E+02	1.6	cytochrome P450 51 cyp51
247	BCG_0817c	Rv0765c				253	Up	1.0E+02	1.6	short chain dehydrogenase
248	BCG_0828c	Rv0776c		-1.18						hypothetical protein
249	BCG_0834c	Rv0783c	*emrB*		1.10					multidrug resistance integral membrane efflux protein
250	BCG_0835	Rv0784		-1.64						hypothetical protein
251	BCG_0836	Rv0785		-1.64						hypothetical protein
252	BCG_0837	Rv0785				111	Up	8.5E+01	1.5	hypothetical protein
253	BCG_0865c	Rv0813c			1.12					hypothetical protein
254	BCG_0866c	Rv3118	*sseC2*		2.16					hypothetical protein
255	BCG_0867c	Rv3117	*cysA2*		2.58					thiosulfate sulfurtransferase cysA2
256	BCG_0877c	Rv0824c	*desA1*		1.21					acyl-ACP desaturase
257	BCG_0878c	Rv0825c				278	Up	5.7E+05	29.4	hypothetical protein
258	BCG_0879	Rv0826				257	Up	5.7E+05	29.4	hypothetical protein
259	BCG_0880c	Rv0827c		1.24						transcriptional regulatory protein
260	BCG_0883	Rv0830			1.02					hypothetical protein
261	BCG_0886c	Rv0834c	*PE_PGRS14*		-1.25					PE-PGRS family protein
262	BCG_0887	Rv0835	*lpqQ*	-1.03	-1.18					lipoprotein lpqQ
263	BCG_0888c	Rv0836c		1.01						hypothetical protein
264	BCG_0900	Rv0848	*cysK2*		1.37					cysteine synthase a cysK2
265	BCG_0901	Rv0849			1.03	68	Up	1.3E+02	1.7	integral membrane transport protein
266	BCG_0902	Rv0850			1.66					transposase
267	BCG_0903c	Rv0851c				91	Up	3.5E+01	1.3	short chain dehydrogenase
268	BCG_0904	Rv0852	*fadD16*			152	Up	3.5E+01	1.3	fatty-acid-CoA ligase fadD16
269	BCG_0908	Rv0856		-1.18	-1.51					hypothetical protein
270	BCG_0910c	Rv0858c				264	Up	4.1E+02	2.3	aminotransferase
271	BCG_0911	Rv0859	*fadA*		-1.47	286	Up	4.1E+02	2.3	acetyl-CoA acetyltransferase
272	BCG_0912	Rv0860	*fadB*		-1.36					fatty oxidation protein fadB
273	BCG_0917	Rv0865	*mog*	1.22						molybdopterin biosynthesis mog protein
274	BCG_0918	Rv0866	*moaE2*		1.57					molybdenum cofactor biosynthesis protein e2 moaE2
275	BCG_0919c	Rv0867c	*rpfA*	-1.25						resuscitation-promoting factor rpfA
276	BCG_0924c	Rv0872c	*PE_PGRS15*		-1.69					PE-PGRS family protein
277	BCG_0927c	Rv0875c			-1.18					hypothetical protein
278	BCG_0937	Rv0885		-2.84						hypothetical protein
279	BCG_0938	Rv0886	*fprB*	-2.64		39	Up	7.5E+02	2.9	DPH:adrenodoxin oxidoreductase fprB
280	BCG_0939c	Rv0887c				156	Up	2.4E+01	1.3	hypothetical protein
281	BCG_0940	Rv0888		-1.51		318	Up	3.2E+01	1.3	hypothetical protein
282	BCG_0943c	Rv0891c				332	Up	5.3E+01	1.4	transcriptional regulatory protein
283	BCG_0944	Rv0892			1.51	612	Up	5.3E+01	1.4	monooxygenase
284	BCG_0945c	Rv0893c				399	Up	4.5E+02	2.3	hypothetical protein
285	BCG_0946	Rv0894				492	Up	4.5E+02	2.3	LuxR family transcriptional regulator
286	BCG_0949c	Rv0897c		1.12						oxidoreductase
287	BCG_0959	Rv0907				92	Up	9.9E+01	1.6	hypothetical protein
288	BCG_0960	Rv0908	*ctpE*			797	Up	9.9E+01	1.6	metal cation transporter atpase P-type ctpE
289	BCG_0963	Rv0911			-1.06					hypothetical protein
290	BCG_0964	Rv0912			-2.06	103	Up	1.2E+01	1.2	hypothetical protein
291	BCG_0967c	Rv0915c	*PPE14*		-1.40					PPE family protein
292	BCG_0968c	Rv0916c	*PE7*	-1.22	-2.25					PE family protein
293	BCG_0980	Rv0928	*pstS3*		1.06					periplasmic phosphate-binding lipoprotein pstS3
294	BCG_0993	Rv0939			-1.29					bifunctional enzyme: 2-hydroxyhepta-2,4-diene-1,7-dioate isomerase + cyclase/dehydrase
295	BCG_0995c	Rv0941c			-1.12					hypothetical protein
296	BCG_0998	Rv0944			-1.15					formamidopyrimidine-D glycosylase
297	BCG_0999	Rv0945			-1.09	229	Up	5.3E+01	1.4	short chain dehydrogenase
298	BCG_1001c	Rv0947c				29	Up	4.4E+01	1.4	mycolyl transferase
299	BCG_1002c	Rv0948c			1.47					hypothetical protein
300	BCG_1003	Rv0949	*uvrD1*	1.26						ATP dependent D helicase uvrD1
301	BCG_1004c	Rv0950c			-5.06					hypothetical protein
302	BCG_1015	Rv0961				18	Up	2.2E+01	1.3	integral membrane protein
303	BCG_1021	Rv0967			1.27					hypothetical protein
304	BCG_1024	Rv0970			1.09					integral membrane protein
305	BCG_1025c	Rv0971c	*echA7*	-1.22	-2.25					enoyl-CoA hydratase
306	BCG_1026c	Rv0972c	*fadE12*	-1.64	-2.74	266	Up	2.5E+01	1.3	acyl-CoA dehydrogenase fadE12
307	BCG_1027c	Rv0973c	*accA2*	-1.15	-2.06	483	Up	4.6E+01	1.4	acetyl-/propionyl-coenzyme A carboxylase alpha chain subunit alpha accA2
308	BCG_1028c	Rv0974c	*accD2*	-1.00	-2.47					acetyl-/propionyl-coa carboxylase subunit beta accD2
309	BCG_1029c	Rv0975c	*fadE13*		-1.51					acyl-coa dehydrogenase fadE13

	ORF			Gene expression		ChIP-seq peaks				
No.	feature	Rv_gene	gene	FC_OE	FC_INH	Distance	position	qvalue	fold enrichment	description
310	BCG_1030c	Rv0976c			-2.00					hypothetical protein
311	BCG_1032c	Rv0978c	*PE_PGRS17*		1.22					PE-PGRS family protein
312	BCG_1035c	Rv0980c	*PE_PGRS18*		1.28					PE-PGRS family protein
313	BCG_1045c	Rv0990c		2.10		153	Up	4.1E+02	2.3	hypothetical protein
314	BCG_1046c	Rv0991c		2.32						hypothetical protein
315	BCG_1049	Rv0994	*moeA1*		-1.25					molybdopterin biosynthesis protein moeA1
316	BCG_1050	Rv0995	*rimJ*		-1.64					ribosomal-protein-alanine acetyltransferase rimJ
317	BCG_1057c	Rv1000c			1.04					hypothetical protein
318	BCG_1061c	Rv1004c		-1.69	-2.74					hypothetical protein
319	BCG_1063	Rv1006			1.40	47	Up	8.1E+02	3.0	hypothetical protein
320	BCG_1066	Rv1009	*rpfB*		-1.06					resuscitation-promoting factor rpfB
321	BCG_1070	Rv1013	*pks16*		1.01					long-chain-fatty-acid--CoA ligase
322	BCG_1073c	Rv1016c	*lpqT*			47	Up	1.1E+02	1.7	lipoprotein lpqT
323	BCG_1079	Rv1022	*lpqU*			74	Up	1.2E+02	1.7	lipoprotein lpqU
324	BCG_1085c	Rv1028c	*kdpD*			435	Up	2.4E+02	2.0	sensor protein kdpD
325	BCG_1086	Rv1028A	*kdpF*			109	Up	2.4E+02	2.0	membrane protein kdpF
326	BCG_1087	Rv1029	*kdpA*			110	Up	2.4E+02	2.0	potassium-transporting ATPase subunit A
327	BCG_1095c	Rv3619c	*esxI*	-1.60						ESAT-6 like protein 1
328	BCG_1096c	Rv1038c	*esxJ*	-1.18						ESAT-6 like protein 2
329	BCG_1097c	Rv1039c	*PPE15*		-2.06					PPE family protein
330	BCG_1098c	Rv1040c	*PE8*		-1.69					PE family protein
331	BCG_1101c	Rv1043c		1.74						hypothetical protein
332	BCG_1102	Rv1044			1.25					hypothetical protein
333	BCG_1103	Rv1045			1.74					hypothetical protein
334	BCG_1110	Rv1052				136	Up	3.9E+01	1.4	hypothetical protein
335	BCG_1111c	Rv1053c				130	Up	6.5E+01	1.5	hypothetical protein
336	BCG_1113	Rv1055				165	Up	7.9E+01	1.5	integrase
337	BCG_1115	Rv1057		-1.79	2.51					hypothetical protein
338	BCG_1116	Rv1058	*fadD14*			55	Up	1.0E+02	1.6	long-chain-fatty-acid--CoA ligase
339	BCG_1120	Rv1062				200	Up	8.2E+01	1.5	hypothetical protein
340	BCG_1123	Rv1065			-1.06					hypothetical protein
341	BCG_1127c	Rv1069c		1.14						hypothetical protein
342	BCG_1128c	Rv1070c	*echA8*	1.41	1.28					enoyl-CoA hydratase
343	BCG_1129c	Rv1071c	*echA9*	1.04	1.30					3-hydroxyisobutyryl-CoA hydrolase
344	BCG_1130	Rv1072		1.48						hypothetical protein
345	BCG_1131	Rv1073		1.10	1.60					hypothetical protein
346	BCG_1132c	Rv1074c	*fadA3*			65	Up	1.2E+03	3.5	acetyl-CoA acetyltransferase
347	BCG_1147	Rv1088	*PE9*		-1.43					PE family protein
348	BCG_1158c	Rv1098c	*fumC*		1.10					fumarate hydratase
349	BCG_1159c	Rv1099c	*glpX*		1.54					fructose 1,6-bisphosphatase II
350	BCG_1161c	Rv1101c		1.08		50	Up	1.1E+03	3.4	hypothetical protein
351	BCG_1162c	Rv1102c			1.44	107	Up	1.1E+02	1.6	hypothetical protein
352	BCG_1172	Rv1112			1.45					GTP-dependent nucleic acid-binding protein EngD
353	BCG_1179c	Rv1118c				243	Up	2.4E+03	4.8	hypothetical protein
354	BCG_1180c	Rv1119c				275	Up	2.4E+03	4.8	hypothetical protein
355	BCG_1187c	Rv1126c				77	Up	1.0E+02	1.6	hypothetical protein
356	BCG_1190c	Rv1129c		-1.09						transcriptional regulator protein
357	BCG_1191	Rv1130		-6.64	-1.40					hypothetical protein
358	BCG_1192	Rv1131	*gltA1*	-5.64	-1.51					citrate synthase
359	BCG_1193	Rv1132		-2.00	-1.36					hypothetical protein
360	BCG_1194c	Rv1133c	*metE*		1.64					5-methyltetrahydropteroyltriglutamate/homocysteine S-methyltransferase
361	BCG_1195	Rv1134				72	Up	1.5E+03	3.8	hypothetical protein
362	BCG_1203c	Rv1141c	*echA11*			313	Up	6.8E+03	7.9	enoyl-CoA hydratase
363	BCG_1206	Rv1144			1.01					short-chain type dehydrogenase/reductase
364	BCG_1207	Rv1146	*mmpL13*		-1.06					transmembrane transport protein mmpL13
365	BCG_1208	Rv1147			-1.22					hypothetical protein
366	BCG_1214c	Rv1153c	*omt*	1.38	1.06					O-methyltransferase omt
367	BCG_1217	Rv1156		1.20						hypothetical protein
368	BCG_1219c	Rv1158c			1.49					hypothetical protein
369	BCG_1223	Rv1161	*narG*	-1.69	-2.32					respiratory nitrate reductase (alpha chain) narG
370	BCG_1224	Rv1162	*narH*	-1.12	-1.64	194	Up	1.4E+04	9.3	respiratory nitrate reductase subunit beta narH
371	BCG_1225	Rv1163	*narJ*	-1.06	-1.22					respiratory nitrate reductase (delta chain) narJ
372	BCG_1229c	Rv1167c		-1.09						transcriptional regulatory protein
373	BCG_1233	Rv1170	*mshB*	-1.06						N-Acetyl-1-D-myo-Inosityl-2-amino-2-deoxy-alpha- D-glucopyranoside deacetylase mshB
374	BCG_1234	Rv1171			-1.06					hypothetical protein
375	BCG_1235c	Rv1172c	*PE12*			168	Up	1.2E+02	1.7	PE family protein
376	BCG_1236	Rv1173	*fbiC*	1.18						FO synthase
377	BCG_1238c	Rv1175c	*fadH*			212	Up	3.7E+03	6.0	DPH dependent 2,4-dienoyl-CoA reductase fadH
378	BCG_1239c	Rv1176c			1.38					hypothetical protein
379	BCG_1240	Rv1177	*fdxC*	1.44	1.53					ferredoxin fdxC
380	BCG_1242c	Rv1179c			-1.29	32	Up	5.0E+01	1.4	hypothetical protein
381	BCG_1243	Rv1181	*pks3*			167	Up	5.0E+01	1.4	polyketide beta-ketoacyl synthase pks3
382	BCG_1245	Rv1183	*mmpL10*		-1.64	46	Up	2.3E+02	1.8	transmembrane transport protein mmpL10
383	BCG_1247c	Rv1185c	*fadD21*			1	Up	3.0E+01	1.3	acyl-CoA synthetase
384	BCG_1248c	Rv1186c		1.34						hypothetical protein
385	BCG_1249	Rv1187	*rocA*		-1.06					pyrroline-5-carboxylate dehydrogenase rocA
386	BCG_1250	Rv1188			-1.40	115	Up	8.0E+01	1.5	proline dehydrogenase

	ORF			Gene expression		ChIP-seq peaks				
No.	feature	Rv_gene	gene	FC_OE	FC_INH	Distance	position	qvalue	fold enrichment	description
387	BCG_1251	Rv1189	*sigI*	-2.06	-2.25					R polymerase sigma factor SigI
388	BCG_1257	Rv1197	*esxK*	-1.29	-1.00					ESAT-6 like protein 3
389	BCG_1265	Rv1205		-1.06						hypothetical protein
390	BCG_1273	Rv1213	*glgC*			114	Up	3.1E+04	13.8	glucose-1-phosphate adenylyltransferase
391	BCG_1274c	Rv1214c	*PE14*	-1.03	-1.56					PE family protein
392	BCG_1275c	Rv1215c			-1.18	256	Up	7.9E+03	5.8	hypothetical protein
393	BCG_1280c	Rv1220c			-1.43					methyltransferase
394	BCG_1281	Rv1221	*sigE*	-1.84						R polymerase sigma factor SigE
395	BCG_1290c	Rv1230c				169	Up	6.4E+01	1.5	hypothetical protein
396	BCG_1295	Rv1235	*lpqY*	1.28	1.17	105	Up	8.2E+01	1.5	sugar-binding lipoprotein lpqY
397	BCG_1296	Rv1236	*sugA*			49	Up	1.8E+02	1.8	sugar-transport integral membrane protein ABC transporter sugA
398	BCG_1297	Rv1237	*sugB*			203	Up	1.6E+01	1.2	sugar-transport integral membrane protein ABC transporter sugB
399	BCG_1300	Rv1240	*mdh*		1.14					malate dehydrogenase
400	BCG_1301	Rv1241		1.40	1.41					hypothetical protein
401	BCG_1302	Rv1242		1.10	1.59					hypothetical protein
402	BCG_1306c	Rv1246c			1.20					hypothetical protein
403	BCG_1314	Rv1254				138	Up	4.8E+01	1.4	acyltransferase
404	BCG_1315c	Rv1255c			1.17					hypothetical protein
405	BCG_1318	Rv1260				2	Up	9.8E+01	1.6	oxidoreductase
406	BCG_1319	Rv1260				18	Up	9.8E+01	1.6	oxidoreductase
407	BCG_1320c	Rv1261c				182	Up	2.6E+01	1.3	hypothetical protein
408	BCG_1326c	Rv1267c	*embR*			407	Up	7.3E+01	1.5	transcriptional regulatory protein embR
409	BCG_1329c	Rv1270c	*lprA*			27	Up	2.0E+01	1.3	lipoprotein lprA
410	BCG_1331c	Rv1272c				613	Up	2.8E+02	2.1	drugs-transport transmembrane ATP-binding protein ABC transporter
411	BCG_1335c	Rv1276c				10	Up	2.9E+01	1.3	hypothetical protein
412	BCG_1336	Rv1277			-1.25					hypothetical protein
413	BCG_1337	Rv1278			-1.47					hypothetical protein
414	BCG_1340c	Rv1281c	*oppD*			93	Up	4.5E+01	1.4	oligopeptide-transport ATP-binding protein ABC transporter oppD
415	BCG_1345	Rv1286	*cysN*			5	Up	2.7E+01	1.3	bifunctional sulfate adenylyltransferase subunit 1/adenylylsulfate kinase
416	BCG_1346	Rv1287				159	Up	3.4E+01	1.3	hypothetical protein
417	BCG_1347	Rv1288				462	Up	3.4E+01	1.3	hypothetical protein
418	BCG_1352	Rv1292	*argS*		1.61					arginyl-tR synthetase
419	BCG_1353	Rv1293	*lysA*		1.12	126	Up	5.8E+01	1.4	diaminopimelate decarboxylase lysA
420	BCG_1354	Rv1294	*thrA*		1.01	123	Up	5.8E+01	1.4	homoserine dehydrogenase
421	BCG_1355	Rv1295	*thrC*			48	Up	2.7E+01	1.3	threonine synthase
422	BCG_1356	Rv1296	*thrB*			45	Up	8.9E+01	1.6	homoserine kinase
423	BCG_1370	Rv1310	*atpD*	1.01						F0F1 ATP synthase subunit beta
424	BCG_1372	Rv1312		1.12						hypothetical protein
425	BCG_1375c	Rv1314c		-1.43						hypothetical protein
426	BCG_1387c	Rv1325c	*PE_PGRS24*		-1.06					PE-PGRS family protein
427	BCG_1388c	Rv1326c	*glgB*			103	Up	1.3E+02	1.7	glycogen branching protein
428	BCG_1392c	Rv1330c				24	Up	2.5E+01	1.3	nicotinate phosphoribosyltransferase
429	BCG_1401	Rv1339		-1.09						hypothetical protein
430	BCG_1413	Rv1351				314	Up	8.7E+02	2.8	hypothetical protein
431	BCG_1414	Rv1352			-1.18	112	Up	8.7E+02	2.8	hypothetical protein
432	BCG_1418c	Rv1356c			1.01					hypothetical protein
433	BCG_1419c	Rv1357c				32	Up	1.1E+01	1.2	hypothetical protein
434	BCG_1429c	Rv1367c				180	Up	5.4E+02	2.5	hypothetical protein
435	BCG_1430	Rv1368	*lprF*			209	Up	5.4E+02	2.5	lipoprotein lprF
436	BCG_1432	Rv1371				195	Up	6.8E+01	1.5	hypothetical protein
437	BCG_1434	Rv1373		1.08						glycolipid sulfotransferase
438	BCG_1441	Rv1380	*pyrB*	1.12						aspartate carbamoyltransferase
439	BCG_1442	Rv1381	*pyrC*		1.17	369	Up	1.4E+02	1.7	dihydroorotase
440	BCG_1446	Rv1385	*pyrF*			390	Up	9.2E+03	8.9	orotidine 5'-phosphate decarboxylase
441	BCG_1447	Rv1386	*PE15*	2.16						PE family protein
442	BCG_1448	Rv1387	*PPE20*	1.97	1.01					PPE family protein
443	BCG_1449	Rv1388	*mihF*		1.16					integration host factor mihF
444	BCG_1450	Rv1389	*gmk*	1.12	1.20					guanylate kinase
445	BCG_1451	Rv1390	*rpoZ*		1.16					D-directed R polymerase subunit omega
446	BCG_1454c	Rv1393c			-1.03					monoxygenase
447	BCG_1462	Rv1401		-1.15	-1.25	30	Up	7.3E+01	1.5	hypothetical protein
448	BCG_1463	Rv1402	*priA*	-1.51	-1.03	80	Up	7.3E+01	1.5	primosome assembly protein PriA
449	BCG_1477	Rv1416	*ribH*			429	Up	7.4E+02	2.9	6,7-dimethyl-8-ribityllumazine synthase
450	BCG_1478	Rv1417				32	Up	7.4E+02	2.9	hypothetical protein
451	BCG_1483	Rv1422				70	Up	1.2E+02	1.7	hypothetical protein
452	BCG_1487c	Rv1426c	*lipO*			52	Up	2.6E+01	1.3	esterase lipO
453	BCG_1496c	Rv1435c		-1.12						hypothetical protein
454	BCG_1500c	Rv1439c				126	Up	1.2E+03	3.4	hypothetical protein
455	BCG_1501	Rv1440	*secG*	-1.09						preprotein translocase subunit SecG
456	BCG_1502c	Rv1441c	*PE_PGR*	-1.22	-1.32					PE-PGRS family protein
457	BCG_1505c	Rv1444c			-1.22					hypothetical protein
458	BCG_1508c	Rv1447c	*zwf2*			331	Up	7.4E+01	1.5	glucose-6-phosphate 1-dehydrogenase
459	BCG_1512	Rv1451	*ctaB*	1.14						protoheme IX farnesyltransferase
460	BCG_1523	Rv1462			1.36					hypothetical protein

	ORF			Gene expression		ChIP-seq peaks				
No.	feature	Rv_gene	gene	FC_OE	FC_INH	Distance	position	qvalue	fold enrichment	description
461	BCG_1530	Rv1469	*ctpD*		1.24					cation transporter P-type atpase D ctpD
462	BCG_1531	Rv1470	*trxA*		1.21					thioredoxin trxA
463	BCG_1532	Rv1471	*trxB1*		1.08					thioredoxin trxB1
464	BCG_1536c	Rv1474c		1.08						transcriptional regulatory protein
465	BCG_1539	Rv1477		-1.18						invasion protein
466	BCG_1540	Rv1478		-1.03						invasion protein
467	BCG_1543	Rv1481				141	Up	1.9E+02	1.9	hypothetical protein
468	BCG_1545	Rv1483	*fabG1*		1.01					3-oxoacyl-ACP reductase
469	BCG_1547	Rv1485	*hemH*		1.37					ferrochelatase
470	BCG_1548c	Rv1486c				92	Up	8.2E+01	1.5	hypothetical protein
471	BCG_1549	Rv1487				35	Up	8.2E+01	1.5	hypothetical protein
472	BCG_1556	Rv1493	*mutB*			38	Up	2.6E+01	1.3	methylmalonyl-CoA mutase
473	BCG_1560	Rv1497	*lipL*		1.06					esterase lipL
474	BCG_1562c	Rv1498A		-1.15						hypothetical protein
475	BCG_1565	Rv1501				128	Up	1.6E+02	1.8	hypothetical protein
476	BCG_1569	Rv1517		-1.12						hypothetical protein
477	BCG_1570	Rv1518				168	Up	1.1E+02	1.6	hypothetical protein
478	BCG_1571	Rv1519			1.17					hypothetical protein
479	BCG_1573	Rv1521	*fadD25*		1.23					acyl-CoA synthetase
480	BCG_1574c	Rv1522c	*mmpL12*			84	Up	1.5E+02	1.7	transmembrane transport protein mmpL12
481	BCG_1575	Rv1523				124	Up	1.5E+02	1.7	methyltransferase
482	BCG_1577	Rv1525	*wbbL2*		-1.06					rhamnosyl transferase wbbL2
483	BCG_1582	Rv1530	*adh*		-1.51	551	Up	2.1E+02	1.9	alcohol dehydrogenase adh
484	BCG_1583	Rv1531				12	Up	2.1E+02	1.9	hypothetical protein
485	BCG_1585	Rv1533		-1.94						hypothetical protein
486	BCG_1586	Rv1534		-1.56						transcriptional regulator
487	BCG_1592	Rv1540		1.33						hypothetical protein
488	BCG_1593c	Rv1541c	*lprI*	-1.56	-1.74					lipoprotein lprI
489	BCG_1594c	Rv1542c	*glbN*	-3.64	-3.84					hemoglobin glbN
490	BCG_1598	Rv1545			-1.25					hypothetical protein
491	BCG_1602	Rv1550	*fadD11*	1.13						fatty-acid-CoA ligase FadD11
492	BCG_1603	Rv1551	*plsB1*	1.23						glycerol-3-phosphate acyltransferase
493	BCG_1604	Rv1552	*frdA*	1.24	-1.69					fumarate reductase flavoprotein subunit
494	BCG_1606	Rv1555	*frdD*		-1.40					fumarate reductase subunit D
495	BCG_1612	Rv1560			1.01					hypothetical protein
496	BCG_1616c	Rv1563c	*treY*			174	Up	1.5E+02	1.7	maltooligosyltrehalose synthase TreY
497	BCG_1618c	Rv1565c			1.45					hypothetical protein
498	BCG_1619c	Rv1566c			1.68					inv protein
499	BCG_1625c	Rv3467			1.05					hypothetical protein
500	BCG_1630c	Rv1592c			1.69					hypothetical protein
501	BCG_1631c	Rv1593c			1.24					hypothetical protein
502	BCG_1632	Rv1594	*nadA*	2.31	1.13					quinolinate synthetase
503	BCG_1633	Rv1595	*nadB*	1.89						L-aspartate oxidase
504	BCG_1637	Rv1599	*hisD*		1.09					histidinol dehydrogenase
505	BCG_1638	Rv1600	*hisC1*			77	Up	1.2E+02	1.7	histidinol-phosphate aminotransferase
506	BCG_1639	Rv1601	*hisB*		1.40					imidazoleglycerol-phosphate dehydratase
507	BCG_1640	Rv1602	*hisH*			215	Up	1.5E+02	1.8	imidazole glycerol phosphate synthase subunit HisH
508	BCG_1645	Rv1607	*chaA*		2.27					ionic transporter integral membrane protein chaA
509	BCG_1647	Rv1609	*trpE*		1.08					anthranilate synthase component I
510	BCG_1650	Rv1612	*trpB*		1.09					tryptophan synthase subunit beta
511	BCG_1659c	Rv1621c	*cydD*			553	Up	1.4E+02	1.7	cytochrome assembly ABC transporter ATP-binding protein CydD
512	BCG_1667	Rv1629	*polA*	1.37						D polymerase I
513	BCG_1669	Rv1631	*coaE*	1.50	1.73					dephospho-CoA kinase/protein folding accessory domain-containing protein
514	BCG_1670c	Rv1632c			-1.36					hypothetical protein
515	BCG_1673c	Rv1635c			1.08					hypothetical protein
516	BCG_1679c	Rv1640c	*lysS*		1.41					lysyl-tR synthetase
517	BCG_1690c	Rv1651c	*PE_PGR*	-1.18	-2.40					PE-PGRS family protein
518	BCG_1692	Rv1653	*argJ*	2.18						bifunctional ornithine acetyltransferase/N-acetylglutamate synthase
519	BCG_1693	Rv1654	*argB*	2.34						acetylglutamate kinase
520	BCG_1694	Rv1655	*argD*	2.34						acetylornithine aminotransferase
521	BCG_1695	Rv1656	*argF*	1.32						ornithine carbamoyltransferase
522	BCG_1696	Rv1657	*argR*	1.13						arginine repressor
523	BCG_1697	Rv1658	*argG*		-1.06					argininosuccinate synthase
524	BCG_1700	Rv1661	*pks7*	-1.51	-1.94					polyketide synthase pks7
525	BCG_1701	Rv1662	*pks8*		-1.94					polyketide synthase
526	BCG_1705c	Rv1666c	*cyp139*			1	Up	1.9E+04	12.0	cytochrome P450 139 CYP139
527	BCG_1708	Rv1670			-1.43					hypothetical protein
528	BCG_1710c	Rv1672c			-1.15					integral membrane transport protein
529	BCG_1713c	Rv1675c				45	Up	4.1E+01	1.3	transcriptional regulatory protein
530	BCG_1715	Rv1677	*dsbF*			209	Up	9.8E+01	1.5	lipoprotein dsbF
531	BCG_1716	Rv1678			-1.29					hypothetical protein
532	BCG_1717	Rv1679	*fadE16*			111	Up	4.4E+01	1.4	acyl-CoA dehydrogenase fadE16
533	BCG_1721	Rv1683		1.13						acyl-CoA synthetase
534	BCG_1723c	Rv1685c				29	Up	7.2E+03	7.4	hypothetical protein
535	BCG_1724c	Rv1686c			1.58					integral membrane protein ABC transporter
536	BCG_1728	Rv1690	*lprJ*		1.65					lipoprotein lprJ

	ORF			Gene expression		ChIP-seq peaks				
No.	feature	Rv_gene	gene	FC_OE	FC_INH	Distance	position	qvalue	fold enrichment	description
537	BCG_1729	Rv1691			1.24					hypothetical protein
538	BCG_1734	Rv1696	*recN*			289	Up	9.1E+01	1.6	D repair protein recN
539	BCG_1739	Rv1701	*xerD*			213	Up	8.1E+01	1.5	site-specific tyrosine recombinase XerD
540	BCG_1744c	Rv1706c	*PPE23*	-1.51	-1.40					PPE family protein
541	BCG_1745c	Rv1706A				76	Up	4.5E+01	1.4	hypothetical protein
542	BCG_1746	Rv1707			1.22					hypothetical protein
543	BCG_1751	Rv1712	*cmk*			18	Up	1.6E+04	12.2	cytidylate kinase
544	BCG_1752	Rv1713	*engA*			22	Up	1.6E+04	12.2	GTP-binding protein EngA
545	BCG_1758	Rv1719		1.40						transcriptional regulatory protein
546	BCG_1767c	Rv1728c				345	Up	3.0E+01	1.3	hypothetical protein
547	BCG_1769c	Rv1730c				317	Up	1.1E+02	1.6	penicillin-binding protein
548	BCG_1771c	Rv1732c		-1.25						hypothetical protein
549	BCG_1772c	Rv1733c		-2.84	-2.00					hypothetical protein
550	BCG_1774c	Rv1735c				35	Up	3.3E+02	2.1	hypothetical protein
551	BCG_1777	Rv1738		-1.25	-1.09					hypothetical protein
552	BCG_1782	Rv1743	*pknE*		1.16					transmembrane serine/threonine-protein kinase E pknE
553	BCG_1791	Rv1752				104	Up	1.6E+02	1.8	hypothetical protein
554	BCG_1793c	Rv1754c			-1.47					hypothetical protein
555	BCG_1817c	Rv1785c	*cyp143*	-1.69						cytochrome P450 143 cyp143
556	BCG_1818	Rv1786		-1.03						ferredoxin
557	BCG_1819	Rv1787	*PPE25*	-1.74	-1.32					PPE family protein
558	BCG_1820	Rv1788	*PE18*	-1.43						PE family protein
559	BCG_1822	Rv1790	*PPE27*	-1.79	-1.32					PPE family protein
560	BCG_1823	Rv1791	*PE19*	-1.74	-2.32					PE family protein
561	BCG_1825	Rv1793	*esxN*		-1.25					ESAT-6 like protein 5
562	BCG_1826	Rv1794		-1.15	-1.84					hypothetical protein
563	BCG_1833	Rv1801	*PPE29*	-1.94	-2.47					PPE family protein
564	BCG_1834	Rv1802	*PPE30*	-1.56	-1.74					PPE family protein
565	BCG_1836c	Rv1803c	*PE_PGR.*	-1.74	-2.25					PE-PGRS family protein
566	BCG_1837c	Rv1804c		-1.32	-1.74					hypothetical protein
567	BCG_1838c	Rv1805c				23	Up	7.2E+01	1.5	hypothetical protein
568	BCG_1839	Rv1806	*PE20*			60	Up	7.2E+01	1.5	PE family protein
569	BCG_1840	Rv1807	*PPE31*			386	Up	7.2E+01	1.5	PPE family protein
570	BCG_1844	Rv1810				52	Up	1.5E+01	1.2	hypothetical protein
571	BCG_1846c	Rv1812c		-1.89	-1.43	134	Up	1.2E+03	3.5	dehydrogenase
572	BCG_1847c	Rv1813c		-2.18						hypothetical protein
573	BCG_1849	Rv1815		-1.03	-2.64					hypothetical protein
574	BCG_1850	Rv1816			-1.94					transcriptional regulatory protein
575	BCG_1859	Rv1824				23	Up	6.6E+01	1.5	hypothetical protein
576	BCG_1869	Rv1834			-1.15					hydrolase
577	BCG_1871c	Rv1836c		1.14	1.33					hypothetical protein
578	BCG_1884	Rv1848	*ureA*	-1.00						urease subunit gamma
579	BCG_1888	Rv1852	*ureG*			158	Up	6.2E+01	1.5	urease accessory protein ureG
580	BCG_1889	Rv1853	*ureD*		-1.12					urease accessory protein ureD
581	BCG_1905c	Rv1869c				94	Up	2.1E+04	15.3	reductase
582	BCG_1919c	Rv1882c			-1.43					short chain dehydrogenase
583	BCG_1921c	Rv1884c	*rpfC*		-2.40					resuscitation-promoting factor rpfC
584	BCG_1922c	Rv1885c			-2.94					chorismate mutase
585	BCG_1923c	Rv1886c	*fbpB*		-2.94					hypothetical protein
586	BCG_1943	Rv1904		-1.60	-2.12					hypothetical protein
587	BCG_1949c	Rv1910c				95	Up	3.2E+01	1.3	hypothetical protein
588	BCG_1951c	Rv1912c	*fadB5*	1.04						oxidoreductase fadB5
589	BCG_1957c	Rv1918c	*PPE35a*			241	Up	3.2E+03	5.5	PPE family protein
590	BCG_1961	Rv1922				141	Up	3.2E+01	1.3	lipoprotein
591	BCG_1972c	Rv1933c	*fadE18*	1.14	1.28					acyl-CoA dehydrogenase fadE18
592	BCG_1977	Rv1938	*ephB*			51	Up	4.8E+01	1.4	epoxide hydrolase ephB
593	BCG_1985c	Rv1946c	*lppG*			69	Up	2.8E+01	1.3	lipoprotein
594	BCG_1986	Rv1947				132	Up	2.8E+01	1.3	hypothetical protein
595	BCG_1994	Rv1955				82	Up	7.2E+03	8.7	hypothetical protein
596	BCG_1997c	Rv1958c			1.16					hypothetical protein
597	BCG_2013	Rv1996		-1.47		99	Up	1.3E+04	11.5	hypothetical protein
598	BCG_2014	Rv1997	*ctpF*	-2.47	-1.64					metal cation transporter P-type atpase A ctpF
599	BCG_2020c	Rv2003c		-1.51	-1.25	265	Up	5.6E+02	2.5	hypothetical protein
600	BCG_2021c	Rv2004c		-1.25	-1.29					hypothetical protein
601	BCG_2022c	Rv2005c		-2.00	-1.22					hypothetical protein
602	BCG_2024c	Rv2007c	*fdxA*	-1.12						ferredoxin fdxA
603	BCG_2038c	Rv2021c		-1.12						hypothetical protein
604	BCG_2043c	Rv2024c				174	Up	8.7E+04	18.7	hypothetical protein
605	BCG_2044c	Rv2025c			1.33					hypothetical protein
606	BCG_2047c	Rv2028c		-1.94	-2.94					hypothetical protein
607	BCG_2048c	Rv2029c	*pfkB*	-1.84	-2.64					phosphofructokinase pfkB
608	BCG_2049c	Rv2030c		-1.18	-2.18					hypothetical protein
609	BCG_2050c	Rv2031c	*hspX*	-1.84	-1.94					heat shock protein hspX
610	BCG_2051	Rv2032		-1.74	-1.29					hypothetical protein
611	BCG_2052c	Rv2033c			-1.40					hypothetical protein
612	BCG_2055	Rv2036				105	Up	4.3E+01	1.4	hypothetical protein
613	BCG_2062c	Rv2043c	*pncA*		-1.03					pyrazinamidase/nicotinamidas pncA
614	BCG_2067c	Rv2048c	*pks12*		1.24					polyketide synthase
615	BCG_2068c	Rv2049c			-1.32					hypothetical protein

	ORF			Gene expression		ChIP-seq peaks				
No.	feature	Rv_gene	gene	FC_OE	FC_INH	Distance	position	qvalue	fold enrichment	description
616	BCG_2071c	Rv2052c			1.34					hypothetical protein
617	BCG_2074c	Rv2055c	*rpsR2*		1.21					30S ribosomal protein S18
618	BCG_2076c	Rv2057c	*rpmG*		1.27	60	Up	8.6E+02	3.0	50S ribosomal protein L33
619	BCG_2077c	Rv2058c	*rpmB*		1.93	59	Up	8.6E+02	3.0	50S ribosomal protein L28
620	BCG_2078	Rv2059			1.20	66	Up	8.6E+02	3.0	hypothetical protein
621	BCG_2083	Rv2064	*cobG*			119	Up	1.3E+02	1.7	cobalamin biosynthesis protein CobG
622	BCG_2095c	Rv2077c				57	Up	1.4E+02	1.7	hypothetical protein
623	BCG_2096c	Rv2077A				57	Up	1.4E+02	1.7	hypothetical protein
624	BCG_2097	Rv2078				821	Up	1.4E+02	1.7	hypothetical protein
625	BCG_2098	Rv2079				911	Up	1.4E+02	1.7	hypothetical protein
626	BCG_2107	Rv2087				118	Up	3.1E+01	1.3	hypothetical protein
627	BCG_2109c	Rv2089c	*pepE*			111	Up	8.4E+01	1.6	dipeptidase PepE
628	BCG_2110	Rv2090				213	Up	8.4E+01	1.6	5'-3' exonuclease
629	BCG_2126c	Rv2109c	*prcA*			61	Up	1.2E+03	3.5	proteasome subunit alpha PrcA
630	BCG_2132c	Rv2115c				62	Up	1.9E+02	1.9	ATPase
631	BCG_2154c	Rv2137c			1.58					hypothetical protein
632	BCG_2155	Rv2138	*lppL*	1.01	1.28					lipoprotein LppL
633	BCG_2159c	Rv2142c			1.36					hypothetical protein
634	BCG_2166c	Rv2149c	*yfiH*		-1.25	157	Up	7.3E+02	2.9	hypothetical protein
635	BCG_2167c	Rv2150c	*ftsZ*		-1.60					cell division protein FtsZ
636	BCG_2179c	Rv2162c	*PE_PGR*	-1.06		218	Up	3.1E+05	28.2	PE-PGRS family protein
637	BCG_2184c	Rv2169c				90	Up	1.0E+02	1.6	hypothetical protein
638	BCG_2193c	Rv2178c	*aroG*	-1.32						3-deoxy-D-arabinoheptulosonate-7-phosphate synthase
639	BCG_2202	Rv2187		-1.12						hypothetical protein
640	BCG_2204c	Rv2188c				60	Up	2.0E+02	1.9	hypothetical protein
641	BCG_2210	Rv2194	*qcrC*	1.26						ubiquinol-cytochrome C reductase QcrC (cytochrome C subunit)
642	BCG_2211	Rv2195	*qcrA*	1.21	1.01					Rieske iron-sulfur protein QcrA
643	BCG_2216c	Rv2200c	*ctaC*	-1.06						transmembrane cytochrome C oxidase (subunit II) ctaC
644	BCG_2219	Rv2203			1.06					hypothetical protein
645	BCG_2227c	Rv2211c	*gcvT*		1.17					glycine cleavage system aminomethyltransferase T
646	BCG_2231	Rv2215	*dlaT*		1.03					dihydrolipoamide acetyltransferase
647	BCG_2232	Rv2216		1.14						hypothetical protein
648	BCG_2237	Rv2220	*glnA1*		-1.03					glutamine synthetase glnA1
649	BCG_2243	Rv2226		1.16	1.12					hypothetical protein
650	BCG_2245	Rv2227				18	Up	1.9E+02	1.9	hypothetical protein
651	BCG_2250	Rv2232				48	Up	2.9E+02	2.1	hypothetical protein
652	BCG_2257c	Rv2240c			1.32					hypothetical protein
653	BCG_2260	Rv2243	*fabD*		1.97					malonyl CoA-ACP transacylase
654	BCG_2261	Rv2244	*acpP*		2.46					acyl carrier protein
655	BCG_2262	Rv2245	*kasA*		1.66					3-oxoacyl-ACP synthase
656	BCG_2263	Rv2246	*kasB*		2.47					3-oxoacyl-ACP synthase
657	BCG_2264	Rv2247	*accD6*	1.04	1.94					acetyl/propionyl CoA carboxylase subunit beta
658	BCG_2265	Rv2248		1.71	2.98					hypothetical protein
659	BCG_2266	Rv2248		1.79	3.04					hypothetical protein
660	BCG_2267c	Rv2249c	*glpD1*	2.28						glycerol-3-phosphate dehydrogenase glpd1
661	BCG_2268c	Rv2250c		2.11						transcriptional regulatory protein
662	BCG_2269	Rv2251		2.16						flavoprotein
663	BCG_2270	Rv2252		2.20						diacylglycerol kinase
664	BCG_2271	Rv2253		-1.36	-1.47					hypothetical protein
665	BCG_2281c	Rv2264c				136	Up	4.6E+02	2.4	hypothetical protein
666	BCG_2283	Rv2266	*cyp124*	-1.47	-1.79					cytochrome P450 124 CYP124
667	BCG_2285c	Rv2268c	*cyp128*		-1.03					cytochrome P450 128 cyp128
668	BCG_2286c	Rv2269c		-1.15						hypothetical protein
669	BCG_2288	Rv2271			1.14					hypothetical protein
670	BCG_2291c	Rv2274c				60	Up	1.1E+01	1.2	hypothetical protein
671	BCG_2292	Rv2275				335	Up	1.1E+01	1.2	hypothetical protein
672	BCG_2295	Rv2280		1.01						dehydrogenase
673	BCG_2308	Rv2291	*sseB*			87	Up	3.6E+02	2.2	thiosulfate sulfurtransferase SseB
674	BCG_2309c	Rv2293c		-1.09						hypothetical protein
675	BCG_2316c	Rv2300c		-1.40						hypothetical protein
676	BCG_2335c	Rv2314c			-1.03	111	Up	2.6E+04	13.8	hypothetical protein
677	BCG_2338	Rv2317	*uspB*		-1.09					sugar-transport integral membrane protein ABC transporter uspB
678	BCG_2339	Rv2318	*uspC*		-1.36					periplasmic sugar-binding lipoprotein uspC
679	BCG_2341c	Rv2320c	*rocE*		-1.18					cationic amino acid transport integral membrane protein
680	BCG_2342c	Rv2321c	*rocD2*		-1.40					ornithine aminotransferase (C-terminus part) rocD2
681	BCG_2343c	Rv2322c	*rocD1*		-1.74	53	Up	4.1E+01	1.4	ornithine aminotransferase (N-terminus part) rocD1
682	BCG_2345	Rv2324			-1.32					AsnC family transcriptional regulator
683	BCG_2350c	Rv2329c	*narK1*			736	Up	1.5E+02	1.7	nitrite extrusion protein 1 narK1
684	BCG_2351c	Rv2330c	*lppP*			644	Up	1.5E+02	1.7	lipoprotein lppP
685	BCG_2352	Rv2331				183	Up	1.5E+02	1.7	hypothetical protein
686	BCG_2353	Rv2331A				127	Up	1.5E+02	1.7	hypothetical protein
687	BCG_2365	Rv2342		-1.12						hypothetical protein
688	BCG_2366c	Rv2343c	*dnaG*			28	Up	5.3E+02	2.4	D primase
689	BCG_2369c	Rv2346c	*esxO*	-1.06	-1.29					ESAT-6 like protein 6
690	BCG_2375c	Rv2361c				144	Up	2.3E+01	1.3	UDP pyrophosphate synthase
691	BCG_2381c	Rv2367c			1.18					metalloprotease
692	BCG_2387c	Rv2373c	*dnaJ2*		1.01					chaperone protein DnaJ

	ORF			Gene expression		ChIP-seq peaks				
No.	feature	Rv_gene	gene	FC_OE	FC_INH	Distance	position	qvalue	fold enrichment	description
693	BCG_2394c	Rv2380c	*mbtE*			67	Up	1.5E+01	1.2	peptide synthetase mbtE
694	BCG_2395c	Rv2381c	*mbtD*		1.49	25	Up	9.2E+01	1.5	polyketide synthase
695	BCG_2396c	Rv2382c	*mbtC*		2.28					polyketide synthase
696	BCG_2397c	Rv2383c	*mbtB*		1.85					phenyloxazoline synthase mbtB
697	BCG_2400c	Rv2386c	*mbtI*		2.66					salicylate synthase MbtI
698	BCG_2405	Rv2391	*nirA*	1.15	1.50					ferredoxin-dependent nitrite reductase NirA
699	BCG_2406	Rv2392	*cysH*		1.37					phosphoadenosine phosphosulfate reductase
700	BCG_2407	Rv2393			1.09					hypothetical protein
701	BCG_2408	Rv2394	*ggtB*		1.08					gamma-glutamyltranspeptidase precursor ggtB
702	BCG_2417c	Rv2401A		-1.18						hypothetical protein
703	BCG_2420c	Rv2404c	*lepA*		1.39					GTP-binding protein LepA
704	BCG_2424	Rv2408	*PE24*		1.58					PE family-like protein
705	BCG_2425c	Rv2409c			1.14					hypothetical protein
706	BCG_2434c	Rv2418c			1.06	74	Up	6.5E+01	1.5	hypothetical protein
707	BCG_2450c	Rv2431c	*PE25*		1.02					PE family protein
708	BCG_2451c	Rv2432c				10	Up	7.8E+01	1.5	hypothetical protein
709	BCG_2452c	Rv2433c				397	Up	7.8E+01	1.5	hypothetical protein
710	BCG_2453c	Rv2434c				291	Up	2.3E+01	1.3	hypothetical protein
711	BCG_2457c	Rv2438c	*nadE*	1.06						D synthetase
712	BCG_2470c	Rv2450c	*rpfE*	-1.47						resuscitation-promoting factor rpfE
713	BCG_2480c	Rv2460c	*clpP2*		1.23	133	Up	5.6E+01	1.4	ATP-dependent Clp protease proteolytic subunit
714	BCG_2482c	Rv2462c	*tig*		1.01					trigger factor
715	BCG_2497c	Rv2477c			1.29					ABC transporter ATP-binding protein
716	BCG_2503c	Rv2485c	*lipQ*		-1.40					carboxylesterase lipQ
717	BCG_2506c	Rv2487c	*PE_PGRS42ab*		-1.47					PE-PGRS family protein
718	BCG_2515c	Rv2495c	*pdhC*			115	Up	2.3E+03	4.7	branched-chain alpha-keto acid dehydrogenase subunit
719	BCG_2519c	Rv2499c				222	Up	2.0E+01	1.2	oxidase regulatory-related protein
720	BCG_2521c	Rv2501c	*accA1*			197	Up	6.8E+01	1.5	acetyl-/propionyl-coenzyme A carboxylase alpha chain subunit alpha accA1
721	BCG_2534	Rv2513				2	Up	2.1E+04	13.3	hypothetical protein
722	BCG_2536c	Rv2515c			-1.40					hypothetical protein
723	BCG_2545c	Rv2524c	*fas*		1.59					fatty acid synthase fas
724	BCG_2549c	Rv2528c	*mrr*		1.09					restriction system protein mrr
725	BCG_2550	Rv2529			1.05					hypothetical protein
726	BCG_2565	Rv2543	*lppA*		1.13					lipoprotein lppA
727	BCG_2579c	Rv2556c				165	Up	2.3E+02	2.0	hypothetical protein
728	BCG_2580	Rv2557			-1.12	152	Up	2.3E+02	2.0	hypothetical protein
729	BCG_2581	Rv2558			-1.18					hypothetical protein
730	BCG_2583	Rv2560			-1.15					proline and glycine rich transmembrane protein
731	BCG_2590	Rv2567			-1.03					hypothetical protein
732	BCG_2591c	Rv2568c				86	Up	7.5E+01	1.5	hypothetical protein
733	BCG_2600	Rv2577		-1.12						hypothetical protein
734	BCG_2604c	Rv2581c			1.06	158	Up	1.3E+02	1.7	glyoxalase II
735	BCG_2605	Rv2582	*ppiB*	1.29	1.79	108	Up	1.3E+02	1.7	peptidyl-prolyl cis-trans isomerase B ppiB
736	BCG_2611c	Rv2588c	*yajC*			62	Up	5.5E+02	2.2	preprotein translocase subunit YajC
737	BCG_2612	Rv2589	*gabT*			152	Up	5.5E+02	2.2	4-aminobutyrate aminotransferase
738	BCG_2614	Rv2591	*PE_PGRS44*		-1.06					PE-PGRS family protein
739	BCG_2618	Rv2595				39	Up	3.6E+03	6.0	hypothetical protein
740	BCG_2619	Rv2596				159	Up	3.6E+03	6.0	hypothetical protein
741	BCG_2650	Rv2623	*TB31.7*	-1.47	-1.56					hypothetical protein
742	BCG_2651c	Rv2624c		-1.69	-2.18					hypothetical protein
743	BCG_2652c	Rv2625c		-2.00	-2.32					transmembrane alanine and leucine rich protein
744	BCG_2653c	Rv2626c		-1.84		7	Up	7.1E+02	2.7	hypothetical protein
745	BCG_2654c	Rv2627c		-1.84	-1.03					hypothetical protein
746	BCG_2655	Rv2628		-2.40	-1.36	136	Up	3.8E+01	1.4	hypothetical protein
747	BCG_2656	Rv2629		-1.29	-1.56	14	Up	3.8E+01	1.4	hypothetical protein
748	BCG_2657	Rv2630		-1.22	-1.74	91	Up	1.9E+01	1.3	hypothetical protein
749	BCG_2658	Rv2631		-1.15	-1.36					hypothetical protein
750	BCG_2659c	Rv2632c			-1.22					hypothetical protein
751	BCG_2660c	Rv2633c		-1.60	-1.56					hypothetical protein
752	BCG_2661c	Rv2634c	*PE_PGRS46*		-1.60					PE-PGRS family protein
753	BCG_2665	Rv2638			-1.94					hypothetical protein
754	BCG_2670	Rv2643	*arsC*	1.21						arsenic-transport integral membrane protein arsC
755	BCG_2694	Rv2681			-1.22					hypothetical protein
756	BCG_2699c	Rv2686c				62	Up	2.3E+01	1.3	antibiotic ABC transporter integral membrane protein
757	BCG_2711	Rv2698			1.10					alanine rich transmembrane protein
758	BCG_2717	Rv2704			1.04					hypothetical protein
759	BCG_2718c	Rv2705c		-1.51						hypothetical protein
760	BCG_2724	Rv2711	*ideR*	1.28	1.39					Iron-dependent repressor and activator ideR
761	BCG_2725c	Rv2712c			1.01					hypothetical protein
762	BCG_2734c	Rv2721c		-1.18		31	Up	2.2E+02	1.9	transmembrane alanine and glycine rich protein
763	BCG_2735	Rv2722				46	Up	2.2E+02	1.9	hypothetical protein
764	BCG_2736	Rv2723				54	Up	2.2E+02	1.9	integral membrane protein
765	BCG_2737c	Rv2724c	*fadE20*	-1.09						acyl-CoA dehydrogenase fadE20
766	BCG_2746c	Rv2733c			1.23					(dimethylallyl)adenosine tR methylthiotransferase
767	BCG_2749c	Rv2736c	*recX*		-1.09					recombination regulator RecX
768	BCG_2758c	Rv2742c		1.01						hypothetical protein
769	BCG_2761c	Rv2745c				69	Up	6.6E+03	8.3	transcriptional regulatory protein
770	BCG_2762c	Rv2746c	*pgsA3*		-1.22					pgp synthase pgsA3

	ORF			Gene expression		ChIP-seq peaks				
No.	feature	Rv_gene	gene	FC_OE	FC_INH	Distance	position	qvalue	fold enrichment	description
771	BCG_2767	Rv2751				159	Up	1.5E+01	1.2	hypothetical protein
772	BCG_2768c	Rv2752c				173	Up	7.5E+01	1.5	hypothetical protein
773	BCG_2770c	Rv2754c	*thyX*		1.54					FAD-dependent thymidylate synthase
774	BCG_2771c	Rv2755c	*hsdS'*			123	Up	6.1E+01	1.5	type I restriction/modification system specificity determinant hsdS'
775	BCG_2772c	Rv2756c	*hsdM*			149	Up	8.7E+01	1.6	type I restriction/modification system D methylase hsdM
776	BCG_2773c	Rv2757c				147	Up	1.3E+01	1.2	hypothetical protein
777	BCG_2777c	Rv2761c	*hsdS*			62	Up	1.2E+02	1.7	type I restriction/modification system specificity determinant hsdS
778	BCG_2781c	Rv2764c	*thyA*	1.02						thymidylate synthase
779	BCG_2782	Rv2765				72	Up	1.5E+02	1.8	alanine rich hydrolase
780	BCG_2784c	Rv2767c		-1.18	-2.00					hypothetical protein
781	BCG_2785c	Rv2768c	*PPE43*	-1.43	-2.12					PPE family protein
782	BCG_2786c	Rv2769c	*PE27*		-1.51					PE family protein
783	BCG_2788c	Rv2771c			-1.60					hypothetical protein
784	BCG_2791c	Rv2774c				181	Up	1.3E+03	3.6	hypothetical protein
785	BCG_2792	Rv2775				219	Up	1.3E+03	3.6	hypothetical protein
786	BCG_2802c	Rv2784c	*lppU*	1.82	2.21					hypothetical protein
787	BCG_2807c	Rv2789c	*fadE21*			316	Up	6.3E+01	1.5	acyl-CoA dehydrogenase fadE21
788	BCG_2810c	Rv2792c				145	Up	8.5E+00	1.2	resolvase
789	BCG_2812c	Rv2794c				10	Up	2.4E+02	2.0	hypothetical protein
790	BCG_2830	Rv2812				172	Up	9.2E+01	1.5	transposase
791	BCG_2831	Rv2813				84	Up	6.7E+01	1.4	hypothetical protein
792	BCG_2832c	Rv3475				80	Up	1.7E+02	1.7	transposase
793	BCG_2837c	Rv2818c				246	Up	3.8E+01	1.4	hypothetical protein
794	BCG_2852c	Rv2832c	*ugpC*		-1.32					sn-glycerol-3-phosphate transport ATP-binding protein ABC transporter ugpC
795	BCG_2853c	Rv2833c	*ugpBa*		-1.47					sn-glycerol-3-phosphate-binding lipoprotein ugpB
796	BCG_2854c	Rv2834c	*ugpE*		-1.18	110	Up	3.4E+01	1.3	sn-glycerol-3-phosphate transport integral membrane protein ABC transporter ugpE
797	BCG_2856c	Rv2836c	*dinF*	1.08	1.01					D-damage-inducible protein F dinF
798	BCG_2865c	Rv2845c	*proS*		1.76					prolyl-tR synthetase
799	BCG_2866c	Rv2846c	*efpA*		2.46					integral membrane efflux protein efpA
800	BCG_2868c	Rv2848c	*cobB*			74	Up	5.7E+01	1.4	cobyrinic acid a,c-diamide synthase
801	BCG_2869c	Rv2849c	*cobO*			31	Up	5.7E+01	1.4	cob(I)yrinic acid a,c-diamide adenosyltransferase
802	BCG_2873	Rv2853	*PE_PGRS48*		-1.32					PE-PGRS family protein
803	BCG_2875	Rv2855	*mtr*			148	Up	8.1E+01	1.5	mycothione reductase
804	BCG_2876	Rv2856	*nicT*			90	Up	4.4E+01	1.4	nickel-transport integral membrane protein nicT
805	BCG_2879c	Rv2857c				607	Up	1.2E+02	1.7	short chain dehydrogenase
806	BCG_2886c	Rv2864c			-1.12					penicillin-binding lipoprotein
807	BCG_2889c	Rv2867c			-1.09					hypothetical protein
808	BCG_2897	Rv2875	*mpb70*	-1.79	-1.74					hypothetical protein
809	BCG_2898	Rv2876		-1.60	-1.60					hypothetical protein
810	BCG_2905	Rv2884		-1.18	-1.94					transcriptional regulatory protein
811	BCG_2920c	Rv2899c	*fdhD*			2	Up	8.5E+00	1.2	formate dehydrogenase accessory protein
812	BCG_2925c	Rv2904c	*rplS*		1.10					50S ribosomal protein L19
813	BCG_2934c	Rv2913c			2.40					D-amino acid aminohydrolase
814	BCG_2939c	Rv2918c	*glnD*		-1.60	205	Up	1.3E+03	3.6	PII uridylyl-transferase
815	BCG_2940c	Rv2919c	*glnB*		-1.25					nitrogen regulatory protein P-II glnB
816	BCG_2941c	Rv2920c	*amt*	-1.43	-2.40					ammonium-transport integral membrane protein amt
817	BCG_2942c	Rv2921c	*ftsY*	1.07		95	Up	1.5E+02	1.8	cell division protein ftsY
818	BCG_2943c	Rv2922c	*smc*			133	Up	5.7E+01	1.4	chromosome partition protein smc
819	BCG_2950	Rv2928	*tesA*	1.34	1.40					thioesterase tesA
820	BCG_2953	Rv2931	*ppsA*		2.48					phenolpthiocerol synthesis type-I polyketide synthase
821	BCG_2954	Rv2932	*ppsB*		2.42					phenolpthiocerol synthesis type-I polyketide synthase
822	BCG_2955	Rv2933	*ppsC*		1.86					phenolpthiocerol synthesis type-I polyketide synthase
823	BCG_2956	Rv2934	*ppsD*			65	Up	2.9E+01	1.3	phenolpthiocerol synthesis type-I polyketide synthase
824	BCG_2961	Rv2939	*papA5*		1.26					acyltransferase
825	BCG_2962c	Rv2940c	*mas*	1.30	2.08					multifunctional mycocerosic acid synthase membrane-associated mas
826	BCG_2963	Rv2941	*fadD28*		1.82					acyl-CoA synthetase
827	BCG_2964	Rv2942	*mmpL7*		1.64					transmembrane transport protein mmpL7
828	BCG_2966	Rv2943A				49	Up	2.9E+02	2.1	transposase
829	BCG_2967c	Rv2945c	*lppX*	1.24	1.91					lipoprotein lppX
830	BCG_2968c	Rv2946c	*pks1*	1.49	2.08					polyketide synthase pks1
831	BCG_2969c	Rv2948c	*fadD22*		1.24					acyl-CoA synthetase
832	BCG_2970c	Rv2949c		-1.00						hypothetical protein
833	BCG_2979c	Rv2958c			1.20					glycosyl transferase family protein
834	BCG_2983c	Rv2962c			1.08					glycosyl transferase family protein
835	BCG_2992	Rv2970A				97	Up	4.9E+02	2.5	hypothetical protein
836	BCG_2993	Rv2971			1.23	170	Up	4.9E+02	2.5	oxidoreductase
837	BCG_2994c	Rv2972c			1.14	24	Up	5.9E+01	1.5	hypothetical protein
838	BCG_2995c	Rv2973c	*recG*		1.10					ATP-dependent D helicase RecG
839	BCG_2996c	Rv2974c			1.28					hypothetical protein
840	BCG_3004	Rv2983		-1.22	-1.40					hypothetical protein
841	BCG_3005	Rv2984	*ppk*			433	Up	2.3E+02	2.0	polyphosphate kinase
842	BCG_3009c	Rv2988c	*leuC*	-1.29						isopropylmalate isomerase large subunit
843	BCG_3010	Rv2989		-1.40						transcriptional regulatory protein
844	BCG_3011c	Rv2990c			2.57					hypothetical protein

	ORF			Gene expression		ChIP-seq peaks				
No.	feature	Rv_gene	gene	FC_OE	FC_INH	Distance	position	qvalue	fold enrichment	description
845	BCG_3020c	Rv2998A				67	Up	3.5E+01	1.3	hypothetical protein
846	BCG_3021	Rv2999	*lppY*			193	Up	3.5E+01	1.3	lipoprotein lppY
847	BCG_3022	Rv3000				34	Up	4.6E+01	1.4	hypothetical protein
848	BCG_3023c	Rv3001c	*ilvC*	1.00		38	Up	4.2E+01	1.4	ketol-acid reductoisomerase
849	BCG_3025c	Rv3003c	*ilvB1*		1.01					acetolactate synthase 1 catalytic subunit
850	BCG_3031c	Rv3009c	*gatB*	1.07						aspartyl/glutamyl-tR amidotransferase subunit B
851	BCG_3034c	Rv3012c	*gatC*		1.18					aspartyl/glutamyl-tR amidotransferase subunit C
852	BCG_3035	Rv3013		-1.06	-1.60					hypothetical protein
853	BCG_3042c	Rv3019c	*esxR*		1.32	22	Up	1.6E+01	1.2	ESAT-6 like protein 9 esxR
854	BCG_3047c	Rv3024c	*mnmA*			142	Up	1.5E+02	1.8	tR-specific 2-thiouridylase MnmA
855	BCG_3051c	Rv3028c	*fixB*	1.43	1.50					electron transfer flavoprotein (alpha-subunit) fixB
856	BCG_3068	Rv3044	*fecB*		1.16					FeIII-dicitrate-binding periplasmic lipoprotein fecB
857	BCG_3072c	Rv3048c	*nrdF*	-1.09						ribonucleotide-diphosphate reductase subunit beta
858	BCG_3073c	Rv3049c				48	Up	1.5E+04	12.5	monooxygenase
859	BCG_3076c	Rv3052c	*nrdI*		-1.51					ribonucleotide reductase stimulatory protein
860	BCG_3079c	Rv3054c			-1.40	193	Up	1.7E+02	1.8	hypothetical protein
861	BCG_3080	Rv3055				116	Up	1.7E+02	1.8	TetR family transcriptional regulator
862	BCG_3083c	Rv3058c		-1.06						TetR family transcriptional regulator
863	BCG_3086c	Rv3061c	*fadE22*		-1.15					acyl-CoA dehydrogenase fadE22
864	BCG_3095	Rv3070				172	Up	1.0E+02	1.6	camphor resistance protein CrcB
865	BCG_3096	Rv3071			-1.06					hypothetical protein
866	BCG_3100c	Rv3075c		1.28						hypothetical protein
867	BCG_3104c	Rv3079c			-1.06					hypothetical protein
868	BCG_3105c	Rv3080c	*pknK*	1.22						serine/threonine-protein kinase transcriptional regulatory protein pknK
869	BCG_3111	Rv3086	*adhD*		-1.06					zinc-type alcohol dehydrogenase adhD
870	BCG_3112	Rv3087			-1.36					hypothetical protein
871	BCG_3114	Rv3089	*fadD13*		-1.36					chain-fatty-acid-CoA ligase fadD13
872	BCG_3119c	Rv3094c		-1.36						hypothetical protein
873	BCG_3120	Rv3095		-1.25						transcriptional regulator
874	BCG_3130c	Rv3105c	*prfB*		1.09					peptide chain release factor 2
875	BCG_3132c	Rv3107c	*agpS*			373	Up	3.7E+01	1.4	alkyldihydroxyacetonephosphate synthase agpS
876	BCG_3133	Rv3108				166	Up	3.7E+01	1.4	hypothetical protein
877	BCG_3139	Rv3114				61	Up	6.4E+01	1.5	hypothetical protein
878	BCG_3147c	Rv3125c	*PPE49a*		-1.00					PPE family protein
879	BCG_3148c	Rv3126c				19	Up	8.9E+01	1.6	hypothetical protein
880	BCG_3149	Rv3127		-1.60	-1.40	125	Up	8.9E+01	1.6	hypothetical protein
881	BCG_3150c	Rv3128c		-1.74		13	Up	4.3E+02	2.3	hypothetical protein
882	BCG_3151c	Rv3128c			-1.12	94	Up	4.3E+02	2.3	hypothetical protein
883	BCG_3153c	Rv3130c		-1.15						hypothetical protein
884	BCG_3154	Rv3131		-1.40						hypothetical protein
885	BCG_3155c	Rv3132c	*devS*	-1.69	-2.18	136	Up	8.5E+01	1.5	two component sensor histidine kinase devS
886	BCG_3156c	Rv3133c	*devR*	-1.56	-1.94					two component transcriptional regulatory protein DevR
887	BCG_3157c	Rv3134c		-1.84	-2.32					hypothetical protein
888	BCG_3158	Rv3135	*PPE50*	-1.15						PPE family protein
889	BCG_3159	Rv3136	*PPE51*			169	Up	1.0E+02	1.6	PPE family protein
890	BCG_3162	Rv3139	*fadE24*		1.94					acyl-CoA dehydrogenase fadE24
891	BCG_3163	Rv3140	*fadE23*		1.77					acyl-CoA dehydrogenase fadE23
892	BCG_3165c	Rv3142c		-1.43	-1.12					hypothetical protein
893	BCG_3167c	Rv3144c	*PPE52*		-1.00					PPE family protein
894	BCG_3171	Rv3148	*nuoD*			62	Up	8.2E+00	1.2	DH dehydrogenase subunit D
895	BCG_3176	Rv3153	*nuoI*			135	Up	1.8E+01	1.2	DH dehydrogenase subunit I
896	BCG_3184c	Rv3160c			1.31					TetR family transcriptional regulator
897	BCG_3185c	Rv3161c			2.13					dioxygenase
898	BCG_3186c	Rv3162c			-1.32					integral membrane protein
899	BCG_3187c	Rv3163c			-1.29	198	Up	9.2E+01	1.5	hypothetical protein
900	BCG_3193	Rv3169			-1.22					hypothetical protein
901	BCG_3194	Rv3170	*aofH*		-1.09					flavin-containing monoamine oxidase AofH
902	BCG_3195c	Rv3171c	*hpx*		-1.15	206	Up	5.2E+01	1.4	non-heme haloperoxidase hpx
903	BCG_3208	Rv3182				4	Up	3.6E+03	5.9	hypothetical protein
904	BCG_3209	Rv3183				118	Up	3.6E+03	5.9	transcriptional regulatory protein
905	BCG_3226c	Rv3201c			-1.03					ATP-dependent D helicase
906	BCG_3231c	Rv3205c				255	Up	2.7E+02	2.0	hypothetical protein
907	BCG_3236	Rv3209			1.58					hypothetical protein
908	BCG_3240c	Rv3213c				127	Up	2.9E+02	2.1	soj/parA-related protein
909	BCG_3242	Rv3215	*entC_1*			81	Up	1.7E+02	1.8	isochorismate synthase entC
910	BCG_3243	Rv3216				10	Up	1.7E+02	1.8	acetyltransferase
911	BCG_3246	Rv3219	*whiB1_1*	-1.69		90	Up	1.1E+02	1.6	transcriptional regulatory protein whiB-like whiB1
912	BCG_3251c	Rv3223c	*sigH_1*	1.04						R polymerase sigma factor RpoE
913	BCG_3266c	Rv3237c		-1.09	-1.74					hypothetical protein
914	BCG_3267c	Rv3238c				216	Up	5.6E+02	2.6	integral membrane protein
915	BCG_3270c	Rv3241c		-1.06						hypothetical protein
916	BCG_3271c	Rv3242c				193	Up	1.9E+05	26.9	hypothetical protein
917	BCG_3273c	Rv3244c	*lpqB*		-1.74					lipoprotein LpqB
918	BCG_3274c	Rv3245c	*mtrB*		-1.56					two component sensory transduction histidine kinase
919	BCG_3275c	Rv3246c	*mtrA*		-1.32					two component sensory transduction transcriptional regulatory protein mtrA
920	BCG_3288	Rv3259			1.91					hypothetical protein
921	BCG_3292	Rv3263			-1.36					D methylase

	ORF			Gene expression		ChIP-seq peaks				
No.	feature	Rv_gene	gene	FC_OE	FC_INH	Distance	position	qvalue	fold enrichment	description
922	BCG_3302	Rv3273				134	Up	4.8E+02	2.4	transmembrane carbonic anhydrase
923	BCG_3306	Rv3277			1.03					hypothetical protein
924	BCG_3311	Rv3282		1.38	1.72	65	Up	1.6E+02	1.8	Maf-like protein
925	BCG_3314	Rv3285	*accA3*		1.14					bifunctional protein acetyl-/propionyl-coenzyme A carboxylase (alpha chain) accA3: biotin carboxylase + biotin carboxyl carrier protein
926	BCG_3315c	Rv3286c	*sigF*		-1.06					R polymerase sigma factor SigF
927	BCG_3316c	Rv3287c	*rsbW*		-1.79					anti-sigma factor rsbW
928	BCG_3317c	Rv3288c	*usfY*		-1.09					protein usfY
929	BCG_3318c	Rv3289c			-1.56					hypothetical protein
930	BCG_3319c	Rv3290c	*lat*	-2.56	-1.09					L-lysine aminotransferase
931	BCG_3321	Rv3292				11	Up	4.4E+02	2.4	hypothetical protein
932	BCG_3327c	Rv3298c	*lpqC_1*		-1.06					esterase lipoprotein lpqC
933	BCG_3328c	Rv3299c	*atsB*		-1.64	43	Up	2.0E+02	1.8	arylsulfatase atsB
934	BCG_3329c	Rv3300c				20	Up	3.8E+01	1.3	hypothetical protein
935	BCG_3333c	Rv3213c				84	Up	1.9E+02	1.8	soj/parA-related protein
936	BCG_3335	Rv3215	*entC_2*			61	Up	1.0E+02	1.6	isochorismate synthase entC
937	BCG_3336	Rv3216				155	Up	7.4E+01	1.5	acetyltransferase
938	BCG_3339	Rv3219	*whiB1_2*			80	Up	1.2E+02	1.7	transcriptional regulatory protein whiB-like whiB1
939	BCG_3354c	Rv3290c	*lat'*	-1.79						L-lysine-epsilon aminotransferase lat'
940	BCG_3364c	Rv3299c	*atsB_2*			64	Up	1.9E+02	1.8	arylsulfatase atsB
941	BCG_3365c	Rv3300c				25	Up	4.1E+01	1.4	hypothetical protein
942	BCG_3370c	Rv3305c	*amiA1*			347	Up	2.0E+02	1.9	N-acyl-L-amino acid amidohydrolase amiA1
943	BCG_3375	Rv3310	*sapM*	-1.18	1.15					acid phosphatase
944	BCG_3376	Rv3311			1.11					hypothetical protein
945	BCG_3379c	Rv3313c	*add*	-1.18						adenosine deaminase
946	BCG_3388c	Rv3322c			-1.74					methyltransferase
947	BCG_3390c	Rv3324c	*moaC*		-1.18					molybdenum cofactor biosynthesis protein MoaC
948	BCG_3391c	Rv3324A	*moaB3*		-1.22					pterin-4-alpha-carbinolamine dehydratase moaB3
949	BCG_3398c	Rv3328c	*sigJ*	-1.09						R polymerase sigma factor SigJ
950	BCG_3403c	Rv3333c				24	Up	3.4E+03	5.8	hypothetical protein
951	BCG_3405	Rv3334				118	Up	3.4E+03	5.8	MerR family transcriptional regulator
952	BCG_3408	Rv3338		-1.36						hypothetical protein
953	BCG_3412	Rv3342				15	Up	5.6E+01	1.4	methyltransferase
954	BCG_3414c	Rv3345c	*PE_PGRS50*		-1.74					hypothetical protein
955	BCG_3418	Rv3844			-1.06					transposase
956	BCG_3424c	Rv3352c		-1.47						oxidoreductase
957	BCG_3426	Rv3354		-2.25						hypothetical protein
958	BCG_3433c	Rv3361c				121	Up	1.4E+02	1.7	hypothetical protein
959	BCG_3439	Rv3367	*PE_PGRS51*		-1.36					PE-PGRS family protein
960	BCG_3443	Rv3371		-2.00	-2.64					hypothetical protein
961	BCG_3450c	Rv3379c	*dxs2*			571	Up	1.4E+02	1.7	1-deoxy-D-xylulose-5-phosphate synthase
962	BCG_3460	Rv3391	*acrA1*	-2.06						short chain dehydrogenase
963	BCG_3461c	Rv3392c	*cmaA1*			204	Up	1.1E+02	1.6	cyclopropane-fatty-acyl-phospholipid synthase 1 cmaA1
964	BCG_3462	Rv3393	*iunH*		-1.15	227	Up	1.1E+02	1.6	nucleoside hydrolase iunH
965	BCG_3466c	Rv3396c	*guaA*			35	Up	1.5E+02	1.7	GMP synthase
966	BCG_3467c	Rv3397c	*phyA*			46	Up	1.5E+02	1.7	phytoene synthase phyA
967	BCG_3468c	Rv3398c	*idsA1*			339	Up	1.5E+02	1.7	multifunctional geranylgeranyl pyrophosphate synthetase idsA1
968	BCG_3469	Rv3399				317	Up	1.5E+02	1.7	hypothetical protein
969	BCG_3472c	Rv3402c			1.42					hypothetical protein
970	BCG_3476	Rv3406		1.36						dioxygenase
971	BCG_3486	Rv3416	*whiB3*	-2.18						transcriptional regulatory protein whiB-like whib3
972	BCG_3487c	Rv3417c	*groEL*	4.80	1.22					chaperonin GroEL
973	BCG_3488c	Rv3418c	*groES*	4.68	1.93					co-chaperonin GroES
974	BCG_3491c	Rv3421c				229	Up	2.1E+02	1.9	hypothetical protein
975	BCG_3492c	Rv3422c				225	Up	2.1E+02	1.9	hypothetical protein
976	BCG_3494c	Rv3424c		1.30						hypothetical protein
977	BCG_3497c	Rv3431c			-1.47					transposase
978	BCG_3504	Rv3438				10	Up	5.4E+02	2.6	hypothetical protein
979	BCG_3505c	Rv3439c			-1.47					hypothetical protein
980	BCG_3506c	Rv3440c				82	Up	1.3E+03	3.6	hypothetical protein
981	BCG_3510c	Rv3444c	*esxT*	-1.03	-2.40					hypothetical protein
982	BCG_3511c	Rv3445c	*esxU*		-2.25					hypothetical protein
983	BCG_3513c	Rv3447c				987	Up	2.7E+02	1.9	hypothetical protein
984	BCG_3514	Rv3448				530	Up	2.7E+02	1.9	integral membrane protein
985	BCG_3515	Rv3449				40	Up	1.3E+01	1.2	serine protease
986	BCG_3520c	Rv3455c	*truA*	2.08	1.96	319	Up	2.7E+01	1.3	tR pseudouridine synthase A
987	BCG_3521c	Rv3456c	*rplQ*	1.19						50S ribosomal protein L17
988	BCG_3522c	Rv3457c	*rpoA*	1.44	1.53					D-directed R polymerase subunit alpha
989	BCG_3530	Rv3465	*rmlC*			115	Up	1.6E+02	1.7	dTDP-4-dehydrorhamnose 3,5-epimerase rmlC
990	BCG_3533c	Rv3468c	*rmlB2*			183	Up	1.8E+02	1.8	dTDP-glucose 4,6-dehydratase rmlB2
991	BCG_3538c	Rv3473c	*bpoA*			75	Up	8.1E+01	1.5	peroxidase bpoA
992	BCG_3543	Rv3479				28	Up	2.9E+02	2.1	hypothetical protein
993	BCG_3549c	Rv3485c				14	Up	3.1E+05	27.9	short chain dehydrogenase
994	BCG_3550	Rv3486				60	Up	3.1E+05	27.9	hypothetical protein
995	BCG_3551c	Rv3487c	*lipF*	-1.22						esterase/lipase lipF
996	BCG_3557c	Rv3493c		-1.40	-1.29					Mce-associated protein
997	BCG_3558c	Rv3494c	*mce4F*	-1.18	-1.36					Mce-family protein mce4F

	ORF			Gene expression		ChIP-seq peaks				
No.	feature	Rv_gene	gene	FC_OE	FC_INH	Distance	position	qvalue	fold enrichment	description
998	BCG_3572	Rv3508	*PE_PGRS54*		-2.06	255	Up	1.6E+04	14.1	PE-PGRS family protein
999	BCG_3576c	Rv3513c	*fadD18*			291	Up	9.8E+04	25.9	fatty-acid-CoA ligase fadD18
1000	BCG_3577	Rv3514	*PE_PGRS57*		-1.40	246	Up	9.8E+04	25.9	PE-PGRS family protein
1001	BCG_3581c	Rv3518c	*cyp142b*			86	Up	4.4E+01	1.4	cytochrome P450 monooxygenase 142 cyp142b
1002	BCG_3586	Rv3522				13	Up	1.9E+01	1.3	lipid-transfer protein
1003	BCG_3587	Rv3523				269	Up	9.2E+01	1.6	acetyl-CoA acetyltransferase
1004	BCG_3589c	Rv3525c			-1.69					siderophore-binding protein
1005	BCG_3591	Rv3527				30	Up	8.5E+01	1.5	hypothetical protein
1006	BCG_3594c	Rv3530c				211	Up	1.2E+02	1.6	short chain dehydrogenase
1007	BCG_3595c	Rv3531c				4	Up	1.3E+02	1.7	hypothetical protein
1008	BCG_3598c	Rv3534c			-1.51					4-hydroxy-2-ketovalerate aldolase
1009	BCG_3602	Rv3538				177	Up	1.2E+02	1.6	dehydrogenase
1010	BCG_3603	Rv3539	*PPE63*			66	Up	1.2E+02	1.6	PPE family protein
1011	BCG_3604c	Rv3540c	*ltp2*		-1.25					lipid-transfer protein
1012	BCG_3606c	Rv3542c			-1.25	247	Up	5.5E+01	1.4	hypothetical protein
1013	BCG_3607c	Rv3543c	*fadE29*			245	Up	5.5E+01	1.4	acyl-CoA dehydrogenase fadE29
1014	BCG_3612c	Rv3548c				120	Up	1.0E+02	1.6	short chain dehydrogenase
1015	BCG_3616	Rv3552			-1.18	347	Up	5.8E+01	1.4	CoA-transferase subunit beta
1016	BCG_3617	Rv3553			-1.40					oxidoreductase
1017	BCG_3622	Rv3558	*PPE64*	-1.12	-1.18					PPE family protein
1018	BCG_3626	Rv3562	*fadE31*		-1.43					acyl-CoA dehydrogenase fadE31
1019	BCG_3627	Rv3563	*fadE32*		-1.22					acyl-CoA dehydrogenase fadE32
1020	BCG_3629	Rv3565	*aspB*		-1.22	38	Up	5.7E+01	1.4	aspartate aminotransferase
1021	BCG_3633c	Rv3568c	*bphC*		-1.09					biphenyl-2,3-diol 1,2-dioxygenase bphC
1022	BCG_3634c	Rv3569c	*bphD*			24	Up	9.5E+02	3.1	2-hydroxy-6-oxo-6-phenylhexa-2,4-dienoate hydrolase
1023	BCG_3642	Rv3577			-1.22	43	Up	4.5E+01	1.4	hypothetical protein
1024	BCG_3646c	Rv3581c	*ispF*		-1.25					2-C-methyl-D-erythritol 2,4-cyclodiphosphate synthase
1025	BCG_3652c	Rv3587c			-1.29					hypothetical protein
1026	BCG_3655c	Rv3590c	*PE_PGRS58*		-1.47					PE-PGRS family protein
1027	BCG_3656c	Rv3591c			-1.00					hydrolase
1028	BCG_3663c	Rv3598c	*lysS*		1.61					lysyl-tR synthetase
1029	BCG_3668c	Rv3604c			-1.22					transmembrane protein rich in alanine and arginine and
1030	BCG_3676c	Rv3612c			-1.25					hypothetical protein
1031	BCG_3680c	Rv3616c		-1.60	-1.40					hypothetical protein
1032	BCG_3686	Rv3628	*ppa*	1.02						inorganic pyrophosphatase ppa
1033	BCG_3691	Rv3633			-1.12					hypothetical protein
1034	BCG_3694	Rv3636			-1.25					transposase
1035	BCG_3705c	Rv3647c			-1.43					hypothetical protein
1036	BCG_3708	Rv3650	*PE33*			349	Up	2.2E+02	1.9	PE family protein
1037	BCG_3709	Rv3651				26	Up	2.2E+02	1.9	hypothetical protein
1038	BCG_3711	Rv3653	*PE_PGRS61*		-1.03					PE-PGRS family protein
1039	BCG_3712c	Rv3654c			-1.36					hypothetical protein
1040	BCG_3713c	Rv3655c				65	Up	3.3E+01	1.3	hypothetical protein
1041	BCG_3718c	Rv3660c		1.05						hypothetical protein
1042	BCG_3719	Rv3661				227	Up	7.0E+02	2.8	hypothetical protein
1043	BCG_3720c	Rv3662c			-1.56					hypothetical protein
1044	BCG_3723c	Rv3665c	*dppB*			389	Up	3.3E+02	2.1	dipeptide-transport integral membrane protein ABC transporter dppB
1045	BCG_3739	Rv3680			1.14	89	Up	4.0E+01	1.4	anion transporter atpase
1046	BCG_3740c	Rv3681c	*whiB4*		-3.06					transcriptional regulatory protein whiB-like whiB4
1047	BCG_3745c	Rv3686c		-2.18						hypothetical protein
1048	BCG_3748	Rv3689			-1.18					hypothetical protein
1049	BCG_3750	Rv3691			-1.06					hypothetical protein
1050	BCG_3752	Rv3693			-1.47	37	Up	2.6E+01	1.3	hypothetical protein
1051	BCG_3754	Rv3695		-1.09						hypothetical protein
1052	BCG_3756c	Rv3697c			-1.09	188	Up	8.9E+01	1.6	hypothetical protein
1053	BCG_3760c	Rv3701c				377	Up	4.0E+01	1.4	hypothetical protein
1054	BCG_3763c	Rv3704c	*gshA*			40	Up	5.0E+02	2.5	glutamate--cysteine ligase
1055	BCG_3767c	Rv3707c		1.08						hypothetical protein
1056	BCG_3770	Rv3710	*leuA*			157	Up	5.1E+02	2.5	2-isopropylmalate synthase
1057	BCG_3771c	Rv3711c	*dnaQ*		-1.32					D polymerase III subunit epsilon
1058	BCG_3775c	Rv3715c	*recR*			102	Up	1.2E+04	10.2	recombination protein RecR
1059	BCG_3777	Rv3717			1.18					hypothetical protein
1060	BCG_3780	Rv3720				176	Up	1.7E+03	3.5	fatty acid synthase
1061	BCG_3784	Rv3724B	*cut5*			164	Up	9.0E+02	3.0	cutinase precursor cut5
1062	BCG_3788	Rv3728				143	Up	1.6E+02	1.8	hypothetical protein
1063	BCG_3793c	Rv3733c			-1.15					hypothetical protein
1064	BCG_3812c	Rv3753c		-1.09						hypothetical protein
1065	BCG_3814c	Rv3755c			-1.00	119	Up	4.0E+01	1.4	hypothetical protein
1066	BCG_3815c	Rv3756c	*proZ*	-1.18	-2.18					osmoprotectant (glycine betaine/carnitine/choline/l-proline) transport integral membrane protein ABC
1067	BCG_3816c	Rv3757c	*proW*		-1.47					osmoprotectant (glycine betaine/carnitine/choline/l-proline) transport integral membrane protein ABC
1068	BCG_3817c	Rv3758c	*proV*		-1.32					osmoprotectant (glycine betaine/carnitine/choline/l-proline) transport ATP-binding protein ABC transporter
1069	BCG_3818c	Rv3759c	*proX*		-1.47					osmoprotectant (glycine betaine/carnitine/choline/l-proline) binding lipoprotein proX
1070	BCG_3819	Rv3760			-1.84					hypothetical protein
1071	BCG_3821c	Rv3762c			-1.09	209	Up	2.7E+04	13.5	hydrolase

	ORF			Gene expression		ChIP-seq peaks				
**No.**	**feature**	**Rv_gene**	**gene**	**FC_OE**	**FC_INH**	**Distance**	**position**	**qvalue**	**fold enrichment**	**description**
1072	BCG_3822	Rv3763	*lpqH*	-1.32	-2.18	140	Up	2.7E+04	13.5	19 kDa lipoprotein antigen precursor lpqH
1073	BCG_3824c	Rv3765c			1.11					two component transcriptional regulator
1074	BCG_3839	Rv3777				33	Up	9.1E+01	1.6	oxidoreductase
1075	BCG_3851	Rv3789			1.02					hypothetical protein
1076	BCG_3854	Rv3792			1.18					hypothetical protein
1077	BCG_3855	Rv3793	*embC*	1.06						integral membrane indolylacetylinositol arabinosyltransferase embC
1078	BCG_3859	Rv3797	*fadE35*	-1.43	-2.12					acyl-CoA dehydrogenase fadE35
1079	BCG_3860	Rv3798			1.26					transposase
1080	BCG_3861c	Rv3799c	*accD4*		1.86					propionyl-CoA carboxylase beta chain 4 accD4
1081	BCG_3862c	Rv3800c	*pks13*		1.79					polyketide synthase
1082	BCG_3863c	Rv3801c	*fadD32*		2.37					long-chain-fatty-acid--CoA ligase
1083	BCG_3864c	Rv3802c			1.04					hypothetical protein
1084	BCG_3865c	Rv3803c	*fbpD*			131	Up	4.1E+02	2.3	hypothetical protein
1085	BCG_3866c	Rv3804c	*fbpA*		1.06					hypothetical protein
1086	BCG_3867c	Rv3805c			1.09					hypothetical protein
1087	BCG_3871c	Rv3809c	*glf*		1.66					UDP-galactopyranose mutase
1088	BCG_3872	Rv3810	*pirG*		2.24					hypothetical protein
1089	BCG_3875c	Rv3813c				15	Up	7.2E+01	1.5	hypothetical protein
1090	BCG_3883	Rv3821			-1.03					integral membrane protein
1091	BCG_3884	Rv3822		-1.47	-1.32					hypothetical protein
1092	BCG_3886c	Rv3823c	*mmpL8*		-1.06					integral membrane transport protein mmpL8
1093	BCG_3896	Rv3833				40	Up	6.1E+02	2.7	AraC family transcriptional regulator
1094	BCG_3909	Rv3846	*sodA*		1.07					superoxide dismutase [Fe] sodA
1095	BCG_3912	Rv3849				65	Up	4.0E+05	28.9	hypothetical protein
1096	BCG_3919c	Rv3856c		1.01						hypothetical protein
1097	BCG_3920c	Rv3857c		1.23						hypothetical protein
1098	BCG_3921c	Rv3858c	*gltD*			397	Up	1.4E+02	1.7	glutamate synthase subunit beta
1099	BCG_3926	Rv3863		1.19	1.06					hypothetical protein
1100	BCG_3927	Rv3864		1.80	1.20					hypothetical protein
1101	BCG_3928	Rv3865		1.30						hypothetical protein
1102	BCG_3930	Rv3867		1.28						hypothetical protein
1103	BCG_3938c	Rv3883c				309	Up	2.1E+01	1.3	protease
1104	BCG_3939c	Rv3884c				112	Up	1.3E+01	1.2	hypothetical protein
1105	BCG_3941c	Rv3886c			-1.15	293	Up	7.1E+01	1.5	hypothetical protein
1106	BCG_3949c	Rv3893c	*PE36*	-1.03						PE family protein
1107	BCG_3950c	Rv3894c				83	Up	3.1E+01	1.3	hypothetical protein
1108	BCG_3951c	Rv3894c				83	Up	1.3E+01	1.2	hypothetical protein
1109	BCG_3953c	Rv3896c				46	Up	2.6E+01	1.3	hypothetical protein
1110	BCG_3960c	Rv3903c				123	Up	3.8E+01	1.4	hypothetical protein
1111	BCG_3962c	Rv3905c	*esxF*		-1.32					ESAT-6 like protein 13 esxF
1112	BCG_3963c	Rv3906c		-1.09	-2.00	121	Up	8.4E+01	1.6	hypothetical protein
1113	BCG_3964c	Rv3907c	*pcnA*	1.03	1.06	149	Up	4.6E+02	2.4	poly(A) polymerase
No.	feature	Rv_gene	*gene*	FC_OE	FC_INH	Distance	position	qvalue	fold enrichment	description

FC_OE, log2 transformed expression value in inbR-overexpressed strain; FC_INH, log2 transformed expression values in INH-treated strain; Distance, the shortest distance from ORF to ChIP-seq peaks; position, relative position of peak to the corresponding ORF; qvalue, peak qvalue reported by peak calling software MACS; fold enrichment, as reported by MACS. Up, upstream.
